# Act or Wait-and-See? Adversity, Agility, and Entrepreneur Wellbeing across Countries during the COVID-19 Pandemic

**DOI:** 10.1177/10422587221104820

**Published:** 2022-06-09

**Authors:** Ute Stephan, Przemysław Zbierowski, Ana Pérez-Luño, Dominika Wach, Johan Wiklund, Marisleidy Alba Cabañas, Edgard Barki, Alexandre Benzari, Claudia Bernhard-Oettel, Janet A. Boekhorst, Arobindu Dash, Adnan Efendic, Constanze Eib, Pierre-Jean Hanard, Tatiana Iakovleva, Satoshi Kawakatsu, Saddam Khalid, Michael Leatherbee, Jun Li, Sharon K. Parker, Jingjing Qu, Francesco Rosati, Sreevas Sahasranamam, Marcus A. Y. Salusse, Tomoki Sekiguchi, Nicola Thomas, Olivier Torrès, Mi Hoang Tran, M.K. Ward, Amanda Jasmine Williamson, Muhammad Mohsin Zahid

**Affiliations:** 1 King’s Business School, 4616 King’s College London, London, UK; 2 Work and Organisational Psychology, 9169 Technische Universität Dresden, Dresden, Germany; 3 Department of Human Resource Management, 256187 University of Economics in Katowice, Katowice, Poland; 4 Business Organization and Marketing Department, 16772 Pablo de Olavide University, Seville, Spain; 5 Department of Work and Organisational Psychology, 9169 Technische Universität Dresden, Dresden, Germany; 6 Whitman School of Management, 2029 Syracuse University, Syracuse, NY, USA; 7 Business School, 27961 Konrad Lorenz University, Bogota, Colombia; 8 Administraçao de Empresas, FGV EAESP (Fundação Getulio Vargas – Escola de Administração de Empresas de Săo Paulo), São Paulo, Brazil; 9 Economics, Human Resources and Diversity Department, 105384 Montpellier Business School, Montpellier, France; 10 Department of Psychology, 7675 Stockholm University, Stockholm, Sweden; 11 Conrad School of Entrepreneurship and Business, 8430 University of Waterloo, Waterloo, ON, Canada; 12 Institute for Management and Organization, 27720 Leuphana University of Lüneburg, Lüneburg, Germany; 13 School of Economics and Business, 166277 University of Sarajevo, Sarajevo, Bosnia and Herzegovina; 14 Department of Psychology, Uppsala University, Uppsala, Sweden; 15 Department of Innovation, Management and Marketing, UiS Business School, University of Stavanger, Stavanger, Norway; 16 Graduate School of Economics, 12918 Kyoto University, Kyoto, Japan; 17 School of Economics and Management, 201138 University of Hyogo, Nishi-ku Kobe, Japan; 18 Departamento de Ingeniería Industrial y de Sistemas, 28033 Pontificia Universidad Católica de Chile, Santiago, Chile; 19 Department of Management, 4013 University of Huddersfield, Huddersfield, UK; 20 Centre for Transformative Work Design, 1649 Curtin University, Perth, WA, Australia; 21 610249 Shanghai Artificial Intelligence Laboratory, Shanghai, China; 22 Centre for Technology Entrepreneurship, 5205 Technical University of Denmark, Kongens Lyngby, Denmark; 23 Hunter Centre for Entrepreneurship, 70446 University of Strathclyde, Glasgow, UK; 24 Center for Entrepreneurship, 154618 Insper Institute of Education and Research, São Paulo, Brazil; 25 Graduate School of Management, Kyoto University, Kyoto, Japan; 26 Brett Centre for Entrepreneurship, 4591 University of Liverpool, Liverpool, UK; 27 Montpellier Management Institute, 27037 University of Montpellier, Montpellier, France; 28 Entrepreneurship, Strategy, Technology, Innovation, Management Department, 105384 Montpellier Business School, Montpellier, France; 29 Zipline.io, Shenton Park, WA, Australia; 30 Waikato Management School, 3717 University of Waikato, Hamilton, New Zealand; 31 5205 Neuron Business and Development Solutions, Islamabad, Pakistan

**Keywords:** entrepreneurship, wellbeing, agility, crisis, life satisfaction, subjective vitality, stress, COVID-19 pandemic, adversity, resilience

## Abstract

How can entrepreneurs protect their wellbeing during a crisis? Does engaging agility (namely, opportunity agility and planning agility) in response to adversity help entrepreneurs safeguard their wellbeing? Activated by adversity, agility may function as a specific resilience mechanism enabling positive adaption to crisis. We studied 3162 entrepreneurs from 20 countries during the COVID-19 pandemic and found that more severe national lockdowns enhanced firm-level adversity for entrepreneurs and diminished their wellbeing. Moreover, entrepreneurs who combined opportunity agility with planning agility experienced higher wellbeing but planning agility alone lowered wellbeing. Entrepreneur agility offers a new agentic perspective to research on entrepreneur wellbeing.

## Introduction

*“Having found myself stuck in Perth, I had to pivot my business and turn to advisory consulting services. However, having started an Engineering company in Thailand and having cemented myself in another culture and business environment, I have found the pandemic has provided the best environment to dig deep and create something from nothing once again.”- Entrepreneur in Australia*

*“[The goal] this year is to stay alive. No business can think of profits and expansions at this time. We don’t know when it will end and what factors will decide the market. It’s really hard to predict the profits, but one thing is sure, as per my experience, every business if continued at all times eventually makes profits. So, hang on and wait for this to pass.”- Entrepreneur in India*

The COVID-19 pandemic is an example of a crisis, that is, a low-probability high-impact situation that creates uncertainty and adversity as it unfolds (Pearson & Clair, 1998; Williams et al., 2017). The pandemic impacted the health, economic, and social life of most individuals and led to the deaths of an estimated 6–19.9 million people.^1^ Governments across the world have implemented “lockdown” measures to varying degrees to curb virus transmission, which has led to the contraction of economies and threatened the success of many businesses and entrepreneurs^2^ (e.g., Bartik et al., 2020; International Trade Centre, 2020).

The wellbeing of entrepreneurs, in particular, might have been threatened during the pandemic because their businesses are closely connected to their identities, and business failure can cause negative emotions (Cardon et al., 2009; Jenkins et al., 2014). Yet, entrepreneur wellbeing is also an important resource for the survival and success of businesses (Stephan, 2018). If entrepreneurs give up, the jobs that their businesses directly and indirectly support disappear. The typically entrepreneur-led small business sector is estimated to provide 70% of global employment (International Labour Organization, 2019). In addition, the societal costs of rebuilding damaged wellbeing are substantial (The Lancet Global Health, 2020). In summary, safeguarding entrepreneur wellbeing during a crisis can help mitigate downstream negative impacts on businesses and society, which is especially important when crises persist over a long period of time. From health pandemics, global recessions to climate change, such crises are predicted to occur more frequently (Mithani, 2020; Phan & Wood, 2020). Investigating entrepreneur wellbeing during the COVID-19 pandemic thus has the potential to offer insights that could be valuable in future crises.

Although research on entrepreneur wellbeing is growing (Wiklund et al., 2019), little is known about how entrepreneur wellbeing is impacted by crises (Doern et al., 2019; Stephan, 2018). Research on entrepreneur wellbeing has identified which stressors and resources impact entrepreneur wellbeing (Stephan, 2018), often applying theories developed for employees (e.g., Hessels et al., 2017); and it has compared the wellbeing of entrepreneurs and employees (Stephan et al., 2022). Existing studies in the context of crises show that crises diminish entrepreneur wellbeing (more than employee wellbeing), mainly because they trigger financial difficulties and threaten the survival of entrepreneurs’ businesses (Backman et al., 2023; Cohidon et al., 2009; de Mel et al., 2008; Doern, 2016; 2017; 2021; Patel & Rietveld, 2020; Torrès et al., 2021; Wolfe & Patel, 2021; Yue & Cowling, 2021).^3^

Research on entrepreneur wellbeing, however, has paid little attention to how the actions entrepreneurs’ take in their business affect their wellbeing—arguably the hallmark of *entrepreneurial* wellbeing (e.g., Rauch et al., 2018; Wiklund et al., 2019).^4^ Such research would recognize the agency of entrepreneurs. This lack of attention to entrepreneur agency in research on wellbeing is particularly surprising considering that, in parallel, rapid response research emphasizes agency while assessing “entrepreneurial” responses to crisis. Focused on predicting business survival and success, this line of research has depicted entrepreneurs as proactively adjusting to crises by pivoting to new opportunities (e.g., Kuckertz et al., 2020; Manolova et al., 2020; Shepherd, 2020) and flexibly adapting planning (e.g., Giones et al., 2020; also Reymen et al., 2015).

Building on insights from strategic management, we suggest that these types of responses can be summarized as *entrepreneur agility* (i.e., flexible and adaptive actions made in response to adversity) (Weber & Tarba, 2014). Entrepreneur agility can be understood as a specific resilience mechanism (Fisher et al., 2019) of in-crisis response and adjustment (Williams et al., 2017) and, thus, positive adaptation to adversity (Fisher et al., 2019; Sutcliffe & Vogus, 2003; Williams et al., 2017).^5^ Agility helps entrepreneurs to navigate environmental changes. It is not a personality trait; instead, it refers to volitional decisions and actions that entrepreneurs, as strategic leaders (Miller, 1983; Yu et al., 2021), take for their businesses.

Strategic management research suggests that agility benefits the survival and performance of businesses (Doz & Kosonen, 2008; Weber & Tarba, 2014). In this study, we ask: *Does engaging entrepreneur agility in response to adversity help entrepreneurs safeguard their wellbeing*? And, will an “act” rather than a “wait-and-see” approach be triggered by crisis adversity and, in turn, help protect entrepreneur wellbeing? Being agile implies that an individual takes charge of a situation, which may lead them to feel more agentic and in control in face of the uncertainty that a crisis brings. Engaging agility may also restore an entrepreneur’s sense of purpose because they are taking meaningful action to protect their business rather than “waiting for this to pass” (as the entrepreneur in our second opening quote put it) and letting the crisis dictate the course of their business. High levels of agility can thus help mitigate the impact of crises on entrepreneur wellbeing.

Entrepreneur agility as a resilience mechanism is not the only possible crisis response (Klyver & Nielsen, 2021; Wenzel et al., 2020). Specifically, threat-rigidity (Staw et al., 1981) has been compared with a resilient approach (Sutcliffe & Vogus, 2003). Threat-rigidity theory suggests that in crises entrepreneurs would become more internally focused on the business, conservative about resources, and may only consider a narrow set of actions. Rather than adapt, entrepreneurs would show more rigidity and “wait-and-see”. The contrast between such a low agility or “wait-and-see” approach and an agile approach can be seen in the opening quotes, where the first entrepreneur shows high agility and the second low agility. Embracing a low agility approach might allow entrepreneurs to conserve precious mental, emotional, and physical energy during an adverse situation that is already straining their wellbeing. Low agility can also mitigate the impact of a crisis on entrepreneur wellbeing by preventing entrepreneurs from trying to adapt to an uncertain and ever-changing situation and experiencing ongoing stress.

In this multi-level study, we investigated 3162 entrepreneurs across 20 countries. Taken together these countries represent three quarters of the global economic output (GDP) and over half of the world’s population. Our study took place during the first wave of the COVID-19 pandemic. We examined how government lockdowns impacted the wellbeing of entrepreneurs in different countries by shaping the adversity that entrepreneurs experienced in their business. Moreover, we integrated research on entrepreneurs’ crisis response in their businesses—which we summarize under the label of entrepreneur agility—with research on entrepreneur wellbeing to explore whether engaging entrepreneur agility in response to adversity helps entrepreneurs to safeguard their wellbeing during the pandemic. Thus, do high agility entrepreneurs who “act” (i.e., start exploring new courses of action when the pandemic adversely impacts the business) or low agility entrepreneurs who “wait-and-see” (i.e., maintain the current way of working until the worst uncertainty is over) experience greater wellbeing?

We found that stringent government lockdowns adversely impacted entrepreneurs’ businesses and reduced their (hedonic and eudaimonic) wellbeing and enhanced their distress. We examined two elements of entrepreneur agility that entrepreneurs engage to deal with this adversity: opportunity agility and planning agility. Opportunity agility (alone and in combination with planning agility) helped entrepreneurs to safeguard their wellbeing, consistent with the resilient “act” approach. However, when not paired with opportunity agility, planning agility alone diminished entrepreneur wellbeing and increased distress, consistent with the “wait-and-see” approach. Consequently, entrepreneurs in crisis benefit from low planning agility when they cannot identify new opportunities (and there is therefore no new goal for which to plan). Finally, adversity activated entrepreneur agility in different ways; it positively influenced planning agility but suppressed opportunity agility.

Our research develops the entrepreneur agility perspective and demonstrates its usefulness in exploring entrepreneur wellbeing during crises. In doing so, it makes several key contributions. First, the entrepreneur agility perspective complements and extends existing research on entrepreneur wellbeing (e.g., Nikolaev et al., 2020; Stephan, 2018; Wiklund et al., 2019) and entrepreneur wellbeing in the context of crisis (e.g., Doern, 2016; Patel & Rietveld, 2020; Torrès et al., 2021; Yue & Cowling, 2021) by offering an agentic perspective that highlights agile “entrepreneurial” action as a driver of entrepreneur wellbeing. Our work theorizes how crises represent challenges to entrepreneurs’ sense of agency (i.e., their perceived ability to engage in self-determined autonomous and purposeful actions) (Deci et al., 2017) and that entrepreneur agility can help safeguard wellbeing through re-asserting agency. In examining the role of entrepreneur agility as a source of agency, this study responds to calls to consider the unique connection between an entrepreneur’s actions and their business for their wellbeing (Wiklund et al., 2019) and shifts attention to a generative perspective considering what entrepreneurs can do to enhance their wellbeing (Williamson et al., 2021).

Second, previous studies have identified different ways that entrepreneurs respond to crises and their effects on business performance and survival (e.g., Giones et al., 2020; Kuckertz et al., 2020; Manolova et al., 2020; Shepherd, 2020). We contribute to this literature by conceptualizing these responses as aspects of entrepreneur agility and by proposing entrepreneur wellbeing as an important microlevel outcome. Moreover, by investigating how entrepreneur agility is activated by adversity and impacts wellbeing across different countries, we shed new light on resilient responses to crisis (Fisher et al., 2019; Williams et al., 2017). This includes demonstrating distinct relationships of adversity and wellbeing with opportunity agility and planning agility indicating support for both “act” and “wait-and-see” approaches; whereby the severity of adversity imparted by the Covid-19 pandemic likely crystallized certain benefits of the “wait-and-see” approach. These findings contribute new insight to research on environmental changes as sources of business opportunities (Kimjeon & Davidsson, 2021) and on the antecedents and consequences of adaptive planning (e.g., Brinckmann et al., 2010; Giones et al., 2020; Sarasvathy, 2001).

Third, because most existing studies do not consider country context (Stephan, 2018; Wiklund et al., 2019), our work contributes a new contextualized perspective to research on entrepreneur wellbeing; and complements conceptual and qualitative work on agility across contexts (Gomes et al., 2020; Shams et al., 2020). Our unique international comparison also strengthens the generalizability of our findings and offers new insights into how new regulations (here, for lockdowns) can impact entrepreneur agility and wellbeing.

## Theory Background and Hypotheses

**Figure 1.**
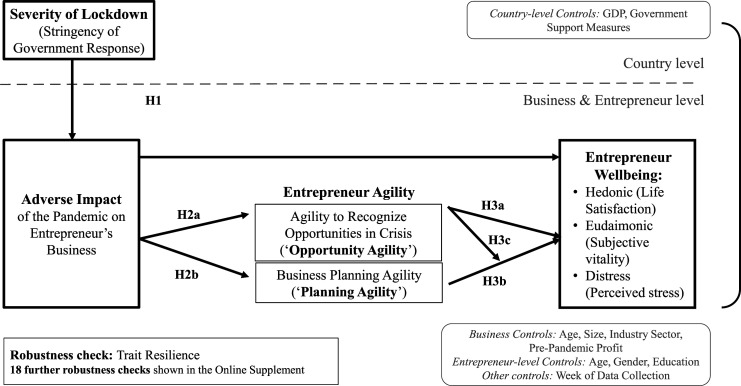
Research framework.

Our research proposes that country-level context (i.e., the severity of lockdown measures adopted in response to the COVID-19 pandemic) diminishes entrepreneur wellbeing by adversely impacting their businesses (H1). We investigated three types of wellbeing: hedonic wellbeing (life satisfaction), eudaimonic wellbeing (subjective vitality), and distress (an indicator of negative wellbeing). We further examined entrepreneur agility as a crisis response strategy that may allow positive adaptation to the COVID-19 pandemic and hypothesized that entrepreneur agility may be activated by the adverse impact of the crisis on entrepreneurs’ businesses (H2). We also predicted that entrepreneurs who respond in an agile manner may enhance their wellbeing and minimize distress (H3). Thus, entrepreneur agility is proposed to mediate the adverse impact of pandemic lockdowns on the wellbeing of entrepreneurs (H4). This framework is depicted in Figure 1.

### Entrepreneur Wellbeing

Wellbeing implies positive experiences and living in a good state (Warr, 2013, p. 77). To fully understand entrepreneur wellbeing, we considered three forms. First, we investigated life satisfaction, which is a widely used indicator of *hedonic wellbeing* (Diener et al., 2018). Second, we examined subjective vitality as an *eudaimonic wellbeing* outcome that comes from engaging in self-regulated action that authentically expresses the self (Deci & Ryan, 2012; Ryan & Frederick, 1997). Subjective vitality centers on positive and energetic feelings that reflect organismic wellbeing (Ryan & Frederick, 1997).^6^ Third, we examine *distress* as a common indicator of negative wellbeing that captures “feeling stressed” (Cohen et al., 1983; Patel & Rietveld, 2020). Distress involves a subjective sense of feeling overwhelmed and occurs when individuals who view their life situation as threatening do not have the required resources to cope, which makes a situation seem unpredictable and uncontrollable (Cohen et al., 1983; Lazarus & Folkman, 1984). Distress thus combines a cognitive component (appraisal) with negative emotions (e.g., feeling upset) (Cohen et al., 1983).

The body of research that has been rapidly growing around entrepreneur wellbeing (for recent reviews: Stephan, 2018; Torrès & Thurik, 2019, and Wiklund et al., 2019) mainly focuses on comparing the wellbeing of entrepreneurs and employees (e.g., Nikolova et al., 2022; Stephan et al., 2022), relating mental health issues to entrepreneurship (e.g., Wiklund et al., 2018; Yu et al., 2021), documenting the effects of wellbeing on entrepreneurial performance (e.g., Gorgievski et al., 2010; Wincent et al., 2008), and charting the wellbeing resources and stressors of entrepreneurs (e.g., Hessels et al., 2017; Lerman et al., 2021; Shir et al., 2019; Wach et al., 2021). The impact of crises on entrepreneur wellbeing was also rarely studied before the COVID-19 pandemic (Doern et al., 2019; Stephan, 2018). Existing research does not, however, develop and test theorizing dedicated to “entrepreneurial” wellbeing that examines how entrepreneurs’ agentic actions in their business are linked to their wellbeing (as called for by Wiklund et al., 2019, also Rauch et al., 2018; Stephan, 2018).

### Crisis, Adversity, and Entrepreneur Wellbeing: The COVID-19 Pandemic

Emerging research on crises and entrepreneur wellbeing portrays crises as stressors (Doern, 2017; Wolfe & Patel, 2021) or “collective stress situation[s]” (Doern, 2016). Stressors are demands that require an exertion of effort and are associated with physiological and/or psychological costs (Bakker & Demerouti, 2007). It is unlikely that a crisis like the COVID-19 pandemic would impact all entrepreneurs in the same way. To understand the impact of crises, it is important to assess both the severity of the crisis (as an objective stressor) as well as the more proximal impact of the crisis on entrepreneurs and their businesses as the experienced stressor. Existing studies that examine COVID-19 and entrepreneur wellbeing focus on single countries and typically use the time of data collection to indicate the existence of the crisis (e.g., Patel & Rietveld, 2020; Torrès et al., 2021), which means that “crisis” is the context of their research. In contrast, our cross-country comparison allows us to determine whether a crisis is present *and* to assess its severity. We exploit the fact that government responses to the pandemic have varied across countries, ranging from stringent legally binding lockdowns to issuing recommendations (Hale et al., 2021). Assessing the stringency of government restrictions or “lockdowns” thus allows us to incorporate the severity of the crisis as an objective stressor. Restrictions on economic activity are arguably the aspect of the crisis that is most relevant to entrepreneurs (Bartik et al., 2020).^7^ In countries with more severe lockdowns, we may expect that entrepreneurs *on average* are more likely to report adverse impacts and may even perceive threats to the survival of their businesses.^8^ In other words, lockdowns (as objective stressors) affect entrepreneurs’ experienced adverse impact on their business (as a subjective stressor).

Because the wellbeing of entrepreneurs is linked to the wellbeing of their businesses (Rauch et al., 2018; Wiklund et al., 2019), the adverse impact of the pandemic on businesses is an important mechanism through which lockdowns diminish entrepreneur wellbeing. This relationship can be understood through self-determination theory (Deci et al., 2017). Entrepreneurship is an agentic activity that offers entrepreneurs a sense of autonomy and purpose that are central to their wellbeing (Shir et al., 2019; Stephan et al., 2020). The pandemic via its adverse impact on their businesses can undermine entrepreneurs’ sense of agency by constraining their possibilities for self-determination.

The pandemic imposed constraints and limited entrepreneurs’ choices, which diminished the sense of agency that is crucial to their wellbeing. Constraints ranged from having fewer customers to reduced financial resources (Bartik et al., 2020; Wolfe & Patel, 2021). Qualitative research has shown that financial constraints limit entrepreneurs’ sense of autonomy in a non-crisis context (van Gelderen, 2016) and that obtaining financial resources can help them re-establish their sense of autonomy in a crisis context (Van Gelderen et al., 2019). Financial constraints can also threaten entrepreneurs’ sense of autonomy and identity (Doern, 2016) because their livelihoods and the livelihoods of their families and employees are dependent on the business (Berrill et al., 2020; Gorgievski et al., 2010). During the pandemic, entrepreneurs have faced enhanced uncertainty about their future and the future of the business (Doern, 2021). Uncertainty is stressful because it undermines agency by diminishing what is perceived to be controllable by the individual (Rauch et al., 2018; Suls & Mullen, 1981; Wincent & Ortqvist, 2009). It is difficult to exert agency and choice when the choice-options themselves are uncertain. Considering this, we propose the following hypothesis:

H1 *Country-level lockdowns impact entrepreneur wellbeing via their adverse impact on entrepreneurs’ businesses. More severe lockdowns lead to more adverse impacts on entrepreneurs’ businesses, which in turn diminish entrepreneurs’ wellbeing (i.e., lower entrepreneurs’ hedonic and eudaimonic wellbeing [life satisfaction and subjective vitality] and enhance their level of distress)*.

### Entrepreneur Agility, Adversity, and Wellbeing

Aside from documenting the negative impact of crises on entrepreneur wellbeing, previous research has not paid attention to how entrepreneurs’ agentic actions may help safeguard wellbeing, despite the fact that conceptual arguments have been made for the close relationship between entrepreneur wellbeing and the actions that they take in business (e.g., Rauch et al., 2018; Wiklund et al., 2019). We thus seek to integrate literature on “entrepreneurial” responses to crisis with research on entrepreneurial wellbeing. Though not concerned with wellbeing, this rapid response and conceptual research highlights examples of how entrepreneurs have made adaptive responses to ensure that their businesses survive and thrive (e.g., Kuckertz et al., 2020; Manolova et al., 2020; Shepherd, 2020) and engaged in flexible adaptive planning (e.g., Giones et al., 2020). Building on insights from the strategic management perspective, we summarize these types of “entrepreneurial” responses as *entrepreneur agility* (flexible and adaptive action in response to adversity) (Weber & Tarba, 2014).

Agility refers to a state of being agile, to readiness and nimbleness, while ‘agile’ is being quick moving, nimble, active (Oxford Dictionary, n.d.). In strategic management, agility is a firm-level concept that helps explain how firms can maintain competitive advantage and high performance by being flexible, adaptable, and responding rapidly to dynamic changes, unexpected challenges, and threats in external business environments (e.g., Ahammad et al., 2020; Doz & Kosonen, 2008). Agile firms are sensitive to changes that occur in their environment and are quick to adapt their strategy and actions accordingly. Agility thus implies two elements: an outward-oriented sensitivity to external opportunities and inward-oriented firm-internal changes in strategy to adapt organizational configurations (Weber & Tarba, 2014). Their relative emphasis can vary; some researchers emphasize recognizing opportunities and others flexible adaptation of strategic planning as opposed to rigid execution of pre-existing plans (e.g., Prange & Hennig, 2019; Shin et al., 2015). Research on agility in strategic management and related fields is either conceptual or qualitative case-based and often nuances these two elements or adds further elements (Brueller et al., 2014; Carmeli et al., 2017; Cunha et al., 2020; Gomes et al., 2020; Lewis et al., 2014; Pereira et al., 2021; Shams et al., 2020).^9^

Translating insights from strategic management research on agility, we can describe *entrepreneur agility* as the flexible and adaptive actions in their business that entrepreneurs take in response to adversity, which involves two elements: outward-oriented recognizing of new opportunities (*opportunity agility*) and inward-oriented adaptation of business planning (*planning agility*). Agility can range from high (the propensity to “act” and engage in flexible and adaptive action) to low (a propensity for inaction and “wait-and-see” approaches), as illustrated by entrepreneurs in the first and second opening quotes, respectively. Entrepreneur agility refers to individual actions that have direct consequences on a business and aligns with strategic leadership theory (Finkelstein & Hambrick, 1996) and the notion that “In an entrepreneurial organization, the entrepreneur is the most important individual, having a disproportional influence on firm strategy and outcomes (Miller, 1983)” (Yu et al., 2021, p.95). Agility emphasizes flexibility and is thus similar to concepts such as effectuation, improvisation, and bricolage (Fisher, 2012; Hmieleski & Corbett, 2008). However, agility gives greater weight to being sensitive to external changes in the environment, such as rapidly noticing and responding to a crisis by exploring new opportunities and adapting plans.

This emphasis on responding to external challenges means that agility is linked to resilience, which describes positive adaptation to adversity or challenging conditions (Fisher et al., 2019; Sutcliffe & Vogus, 2003; Williams et al., 2017). Among the different elements of resilience (Fisher et al., 2019 and Williams et al., 2017 for reviews), entrepreneur agility reflects a specific resilience mechanism (in-crisis responding and adjustment), while the severity of adversity (e.g., the severity of lockdowns) can be understood as the resilience “trigger.”

In response to COVID-19, authors have outlined different strategic crisis responses (e.g., Wenzel et al., 2020; Klyver & Nielsen, 2021), while the literature on resilience has suggested that the key distinction in reactions to adversity lies in the difference between “resilient” (“act”) and “rigid” (“wait-and-see”) responses (Sutcliffe & Vogus, 2003). By and large, the findings of research on resilience and agility are consistent with the “act” approach, as is emerging conceptual and rapid response research in entrepreneurship (e.g., Giones et al., 2020; Kuckertz et al., 2020; Shepherd, 2020). However, both large and small businesses have also been found to adopt a “wait-and-see” approach that delays action in the face of crises, which is a more conservative tactic that helps preserve resources and maintain control in light of crisis-induced uncertainty (Chattopadhyay et al., 2001; Staw et al., 1981; Thorgren & Williams, 2020; Laskovaia et al., 2019).^10^ How will the pandemic impact entrepreneur agility?

We suggest that entrepreneurs typically engage (high) agility as a crisis response strategy triggered by adversity, which aligns with the view that entrepreneurs and small businesses are adaptable (Shepherd, 2020; Smallbone et al., 2012). Unlike larger firms, these types of businesses are unlikely to be able to “afford” to “wait-and-see” (Wenzel et al., 2020) because they have more resource constraints and are less prepared for a crisis (Doern et al., 2019). Consequently, they are more likely to act with agility, especially when their business is adversely impacted and its existence threatened. We thus propose the following hypothesis:

H2 *There is a positive relationship between the adverse impact of the pandemic on entrepreneurs’ businesses and their agility. The more adversely an entrepreneur’s business is impacted by the pandemic, the higher an entrepreneur’s opportunity agility (H2a) and planning agility (H2b) will be*.

We argue that by engaging both opportunity agility and planning agility, entrepreneurs can safeguard their wellbeing because agility helps them re-establish a sense of agency in the face of adversity. When entrepreneurs act with agility, they engage in self-determined actions that help them feel “in control” (similar to problem-focused coping, Lazarus & Folkman, 1984; Patzelt & Shepherd, 2011). Acting with agility may also indirectly enhance entrepreneur wellbeing by removing constraints on agency, especially by addressing financial constraints and alleviating uncertainty. Agile action can help entrepreneurs find new revenue sources by identifying business opportunities and adapting strategy accordingly. Acting with agility likely also helps alleviate perceptions of uncertainty and unpredictability. By seeking out new opportunities and making changes, entrepreneurs focus their attention on the aspects of their current situation that they can change, thereby regaining a sense of control that benefits their wellbeing (Williams & Shepherd, 2016b). We thus propose the following hypothesis:

H3 *Opportunity agility (H3a) and planning agility (H3b) relate positively to entrepreneurs’ hedonic and eudaimonic wellbeing (life satisfaction and vitality) and relate negatively to their distress*.

We further propose that positive effects of engaging agility will be more pronounced when entrepreneurs mobilize both elements of agility. This is because adaptive planning on its own may only provide a small boost to entrepreneurs’ sense of agency when goals are unclear. On the other hand, opportunity agility helps entrepreneurs identify new goals that they can use to plan and thus reduces perceived uncertainty; however, identifying opportunities may only offer limited wellbeing benefits if the opportunities remain abstract. Entrepreneurs’ sense of agency is likely benefited the most when they adapt their business planning to capitalize and commercialize new opportunities. In other words, combining planning agility and opportunity agility will have synergistic positive effects on entrepreneur wellbeing and bolster their sense of agency. We thus propose the following hypothesis:

H3c *Opportunity agility and planning agility have synergistic effects. Opportunity agility and planning agility interact in such a way that entrepreneurs’ hedonic and eudaimonic wellbeing is higher and their distress lower when they engage both elements of agility*.

In summary, our research framework postulates that the more adversely an entrepreneur’s business is impacted by a crisis, the more their wellbeing will suffer and that these adverse impacts differ depending on the severity of the crisis. We also propose that entrepreneur agility is activated by crisis (specifically, by adverse impacts on the business) and functions as a specific resilience mechanism that can help entrepreneurs re-establish their sense of agency and protect their wellbeing. Combining H1 and H3, we propose the following hypothesis:

H4 *Opportunity agility and planning agility mediate the adverse impacts of the pandemic on entrepreneurs’ businesses as well as the effects of the severity of lockdowns on entrepreneurs’ hedonic and eudaimonic wellbeing (life satisfaction and vitality) and distress*.

## Methods

### Sample

We surveyed entrepreneurs (i.e., self-employed, start-up entrepreneurs, and owner-managers of small and medium-sized businesses) in 20 countries across North and South America, Asia, Europe, and Oceania^11^ from mid-April to early September 2020. Our sample includes countries that were among the worst affected (measured by deaths per capita:^12^ Italy, Spain, UK, and the USA) and those with low fatalities (Australia, Japan, and New Zealand) in the first wave of the pandemic. These countries represent 73% of the world’s GDP and are home to 56% of the world’s population.^13^

As we were collecting data during the pandemic, we recruited entrepreneurs through the distribution of an online survey via entrepreneur associations, entrepreneur networks, incubators, banks, and social media. Typically, the local country co-investigator reached out to these actors explaining the purpose of the study, the global nature of the project, and the value of participation^14^. We excluded non-entrepreneurs^15^ from the 5499 participants we obtained, which left *N* = 3755. Another 593 participants were excluded due to missing values on study variables, which left us with a final sample of *N* = 3162 entrepreneurs.^16^ Our analyses apply a range of control variables to account for variations in sample composition across countries (see below). Sample descriptive statistics are included in Table 1. Because entrepreneurs were difficult to reach during the pandemic and we had a broad recruitment strategy, we assessed how representative our sample of entrepreneurs was by comparing it to existing population-representative samples of entrepreneurs from the Global Entrepreneurship Monitor (GEM). As we focused on entrepreneurs running operating businesses, we used new and established entrepreneurs from GEM as a comparison sample; that is, GEM entrepreneurs who were running an operating business and excluding nascent entrepreneurs who were in the process of starting a business. Details about these analyses are included in the online supplement (Tables A23–A28). They indicate that compared with the GEM sample, the entrepreneurs in our sample run slightly younger businesses (0.76 years)^17^, are themselves slightly younger (0.02 years), and are more often men (by 2.6%). They are also better educated (41.8% more university educated) and lead larger businesses (7.55 more employees) that operate more frequently in service industries (by 19.6%). These differences are due to the fact that our survey targeted businesses and their owner-managers, while GEM obtains representative samples of individuals via *households*, which is more likely to pick up self-employment.

**Table 1.** Descriptive Statistics and Correlations on the Individual Level.

		Mean	SD	Min	Max	1	2	3	4	5	6	7	8
1	Life satisfaction	6.76	1.93	1.00	10.00								
2	Subjective vitality	3.40	0.78	1.00	5.00	0.53							
3	Distress	2.71	0.67	1.00	5.00	−0.54	−0.56						
4	Adverse impact on business	1.38	0.96	−1.00	2.00	−0.16	−0.09	0.16					
5	Opportunity agility	0.39	0.49	0.00	1.00	0.13	0.14	−0.09	−0.24				
6	Planning agility	4.35	3.20	0.00	10.00	−0.06	0.00	0.09	0.19	0.18			
7	Firm age	9.98	11.19	0.00	81.00	−0.01	−0.08	−0.01	0.00	−0.08	−0.06		
8	Firm size (log of number employees)	1.64	1.30	0.00	6.32	0.09	0.15	−0.13	0.02	0.08	0.11	0.10	
9	Industry: Business services	0.48	0.50	0.00	1.00	0.04	−0.04	−0.07	−0.16	0.18	−0.01	−0.04	−0.08
10	Industry: Retail and gastronomy	0.20	0.40	0.00	1.00	−0.04	0.03	0.03	0.14	−0.11	0.01	−0.02	0.04
11	Industry: Manufacturing and extractive	0.15	0.36	0.00	1.00	0.03	0.07	−0.03	0.01	−0.07	0.02	0.05	0.20
12	Industry: Human-oriented services	0.12	0.32	0.00	1.00	−0.01	−0.05	0.06	0.06	−0.03	0.01	0.01	−0.12
13	Industry: Other	0.06	0.24	0.00	1.00	−0.05	0.00	0.04	0.02	−0.06	−0.06	0.03	−0.04
14	Firm profit last year	0.72	0.45	0.00	1.00	0.07	0.02	−0.10	0.03	−0.08	−0.04	0.15	0.11
15	Entrepreneur gender	0.33	0.47	0.00	1.00	−0.04	−0.10	0.12	0.04	−0.01	0.01	0.02	−0.15
16	Entrepreneur age	43.48	12.00	18.00	87.00	0.04	−0.05	−0.10	−0.08	−0.01	−0.07	0.38	−0.13
17	Secondary education	0.96	0.18	0.00	1.00	0.02	0.01	0.00	−0.02	0.03	0.03	−0.06	0.08
18	University education	0.73	0.44	0.00	1.00	0.06	0.03	−0.02	−0.04	0.11	0.06	−0.13	0.09
19	Week of data collection	26.12	5.01	16.00	37.00	0.05	0.07	−0.07	−0.05	0.08	−0.01	−0.07	0.21
20	Trait resilience	4.55	0.71	1.00	6.00	0.28	0.33	−0.40	−0.14	0.14	0.04	−0.04	0.01
		9	10	11	12	13	14	15	16	17	18	19	
10	Industry: Retail and gastronomy	−0.48											
11	Industry: Manufacturing and extractive	−0.40	−0.21										
12	Industry: Human-oriented services	−0.35	−0.18	−0.15									
13	Industry: Other	−0.24	−0.12	−0.11	−0.09								
14	Firm profit last year	−0.02	0.04	0.01	−0.01	−0.03							
15	Entrepreneur gender	−0.09	0.09	−0.08	0.12	−0.01	0.02						
16	Entrepreneur age	0.19	−0.19	−0.06	0.02	−0.03	−0.01	0.01					
17	Secondary education	0.05	−0.04	0.01	−0.02	−0.03	0.00	−0.02	−0.03				
18	University education	0.16	−0.10	−0.02	−0.01	−0.12	−0.03	0.03	−0.05	0.31			
19	Week of data collection	0.03	0.00	0.07	−0.16	0.04	−0.05	−0.12	−0.14	0.09	0.10		
20	Trait resilience	0.16	−0.13	−0.04	0.00	−0.07	0.00	−0.01	0.17	−0.01	0.05	0.01	

*Note. N =* 3,162, except for subjective vitality, distress and trait resilience: *N* for correlations with subjective vitality=2,767, for distress *N =* 2,974, for trait resilience *N =* 2,935, *N* between subjective vitality and distress=2,761, *N* between subjective vitality and resilience=2,723, *N* between distress and resilience=2,931. Correlations of *r* ≥0.036 significant at *p < .*05 level, correlations of *r*>=0.047 at *p < .*01. Industry: business services = reference category in regressions.

### Measures

#### Dependent variables: Three types of wellbeing

We measured *hedonic well-being* as *life satisfaction* with a single item 10-point scale from the World Values Survey (WVS) program, which is widely used and validated in research on wellbeing (Diener et al., 2013) and entrepreneurship (e.g., Fritsch et al., 2019; Wolfe & Patel, 2018). We asked, “All things considered, how satisfied are you with your life as a whole these days?” from 1 = completely dissatisfied to 10 = completely satisfied. The measurement reliability of this item was estimated in longitudinal research to range between 0.68 and 0.72 (Lucas & Donnellan, 2012). The average correlation of the single item measure with the well-established 5-item Satisfaction with Life Scale (Diener et al., 1985) was *r* = 0.67, *p < .*001, *N =* 426 across the four countries where we collected data on both (Australia, Poland, Spain, and USA; ranging from *r* = 0.65 for Spain to *r* = 0.80 in Poland). To get a sense of whether entrepreneurs’ life satisfaction was negatively impacted by the pandemic, we compared the life satisfaction in our sample of entrepreneurs to existing pre-pandemic life satisfaction data of population representative samples of the self-employed (*N =* 3941) from the WVS.^18^ An analysis of variance controlling for age, gender, education, and country showed that the entrepreneurs in our sample have statistically significant lower life satisfaction (12% lower) with *F* = 149.47, *p < .*001, partial eta-square = 0.02.

We measured *eudaimonic well-being* as *subjective vitality* with the 7-item scale developed by Ryan and Frederick (1997). An example item is “I feel alive and vital.” Answers were recorded on a 5-point scale ranging from 1 = strongly disagree to 5 = strongly agree. The scale had good reliability (Cronbach Alpha 0.88; Composite Reliability 0.87), and confirmatory factor analysis showed good fit (Satorra–Bentler’s χ^2^ = 161.55, df = 14; CFI = 0.98; GFI = 0.98; AGFI = 0.96; SRMR = 0.02).

We assessed *distress* as a measure of negative wellbeing (or illbeing) with the 10-item Perceived Stress Scale (Cohen et al., 1983). An example item is “In the past month, how often have you been upset because of something that happened unexpectedly?” (answered on a 5-point scale ranging from 1 = never to 5 = very often). The scale had good reliability (Cronbach Alpha 0.86; Composite Reliability 0.84), and confirmatory factor analyses showed good fit (Satorra–Bentler’s χ^2^ = 1049.17, df = 35; CFI = 0.87; GFI = 0.90; AGFI= 0.84; SRMR = 0.07).

### Predictor variables

*Severity of lockdown*. To assess the severity of country-level lockdowns, we used information about the *stringency of government response to the pandemic* collected by the Oxford-COVID-19 Government Response Tracker (Hale et al., 2021). The stringency of government response is based on nine individual indicators that are aggregated to form an index ranging from 1 = low to 100 = maximum stringency. Eight of the nine indicators refer to containment and closure policies^19^. The ninth indicator assesses public information campaigns about the pandemic. The indicators are weighted for geographical scope (i.e., whether a lockdown affected a local area or the entire country). We used the average stringency for 8 months (from January to August 2020).

*Adverse impact of the pandemic on entrepreneurs’ businesses.* We combined information from two questions to assess the degree of adverse impact (higher values represent more adversity, while lower values indicate that the pandemic had a positive impact on the business). The first asked about the impact of the pandemic on trading: “In what way has the COVID-19 pandemic affected your business or your trading conditions?” Entrepreneurs chose one of five options. Some options were rarely selected, which is why we collapsed the first three options (“I had to suspend trading and close my business”; “I had to suspend trading but my business still exists”; “The volume of trading has decreased”) into “decreased trading.” The fourth option reflected “no change” (“Trading continues unchanged”), and the fifth option reflected an “increase” in trading (“The volume of trading has increased”). In the second question, we asked, “Is the existence of your business threatened by the COVID-19 pandemic?” (yes = 1; no = 0). There are different ways to combine these questions into an index. In the main analyses, we used an index that ranges from “2” (the most negative adverse impact on the business) to “-1” (a positive impact on the business). The value of “2” reflects decreased trading and threats to the existence of the business, while the value of “1” reflects decreased trading with no threat to the existence of the business. No change in trading was coded as “0,” and an increase in trading was coded as “-1.” The online supplement (Tables A9–A11) provides robustness checks for slightly different versions of this index, including one that weights the questions equally (Cronbach Alpha 0.69). Our results replicate for all versions of the index indicating robustness.

*Opportunity agility.* We assessed opportunity agility by asking, “Has the current situation opened-up any new business opportunities for you?” (yes = 1; no = 0). The “current situation” was defined as the COVID-19 pandemic earlier in the questionnaire. This question builds on similar questions about opportunity perception for business creation successfully employed in large-scale cross-country comparative studies (e.g., Kwon & Arenius, 2010) and about opportunity identification in a study about supply chain agility (Gligor et al., 2015). Moreover, we analyzed answers to an open-ended follow-up question that inquired about the nature of opportunities. Entrepreneurs reported diverse opportunities relating to accelerated digitalization (e.g., online trade, products/services, and payments), opportunities to enhance environmental and social sustainability (e.g., waste reduction, circular economy, and social inclusion), moving to more local production due to the disruption of global supply chains, and opportunities related to wellbeing and health (e.g., new medical and wellbeing products/services and business expansion due to enhanced awareness about mental health). Thus, entrepreneurs’ answers indicated that they understood our question about business opportunities in the intended manner.

*Planning agility*. We measured entrepreneurs’ planning agility through two questions: “Did the COVID-19 pandemic lead you to change your plans for your business?” (yes = 1; no = 0) and “How far into the COVID-19 pandemic did you start changing your plans or develop alternative plans?” Response categories ranged from 1 = “before January 2020” to 10 = “later,” and categories 2 to 9 represented January 2020, February 2020, early-mid March 2020, mid-to-late March 2020, early-mid April 2020, mid-late April 2020, early-mid May 2020, and mid-late May, respectively. Our measure translates the notion that agile firms adapt their strategy when encountering unexpected difficulties from research on strategic and supply chain agility (Brueller et al., 2014; Gligor et al., 2015) to entrepreneurship. We integrated the two questions into one scaled variable: “planning agility.” We coded the response of “no” to the first question about changed plans as 11 and then substituted the “yes” response with the 10 values from the follow-up question, which we reverse-scored for this purpose. The resulting variable ranges from 0 = no planning agility (no change in plans, which can be viewed as representing a “wait-and-see” approach) to 10 = highest planning agility (i.e., entrepreneurs changed plans before January 2020), with values 1 to 9 representing the months in between.

To strengthen confidence in our measures of opportunity agility and planning agility, we explored evidence for convergent and discriminant validity. In terms of convergent validity, agility as a form of adaptive action should overlap with creativity (i.e., the generation of useful ideas to solve problems) (Amabile, 1988) and with effectuation (Sarasvathy, 2001). For discriminant validity, we would expect no correlation with causation (which is focused on planning and execution rather than flexibility adapting to changing circumstances) (Sarasvathy, 2001). We found evidence for convergent correlations with creativity for opportunity agility and planning agility (*r* = 0.32, *p* < .001 and *r*=0.21, *p* < .001, *N =* 2754; 18 countries except Germany and USA). Data on effectuation and causation comes from the Indian entrepreneurs who participated in our study. Opportunity agility correlates with the opportunity-oriented flexibility facet of effectuation at *r* = 0.21, *p < .*05, *N =* 98 and planning agility with the experimentation facet of effectuation at *r* = 0.26, *p < .*01, *N =* 98. In line with expectations about discriminant validity, there were no significant correlations with causation (*r* = 0.11, n.s. for opportunity agility and *r* = 0.135, n.s. for planning agility, *N =* 98). We used published measures of creativity and effectuation-causation (Chandler et al., 2011; Janssen, 2000; Laskovaia et al., 2017; Weinberger et al., 2018). Details are included in Supplemental Table A30 in the online supplement.

*Control variables*. We included country-level control variables and control variables for the characteristics of the entrepreneurs and their businesses. These account for country differences in the compositions of our samples and help rule out alternative explanations for our findings (e.g., larger businesses and those that were profitable before the pandemic might have more resources to survive longer). *At the country level,* we first controlled for *GDP* per capita in purchase power standards (PPS) in 2019 (US dollars, from the World Bank). Second, to account for differences in *government support* offered to the economy and businesses during the pandemic, we used the “Economic policy response” index from the Oxford-COVID-19 Government Response Tracker (Hale et al., 2021), which indicates if a government is providing direct cash payments to employees who lose their jobs or cannot work, enabling firms to put employees on paid leave and remain afloat by reducing fixed costs. In this index, 0 = no income support provided, 1 = government is replacing less than 50% of lost salary/income, and 2 = government is replacing 50% or more of lost salary/income. As with the index for severity of lockdown, we used the average government support provided from January to August 2020.

In terms of *business characteristics*, we controlled for *firm age* (in years), *firm size* (measured as the log of number of employees, logged due to skewness), and *industry* sector: (1) business services, (2) retail and gastronomy, (3) manufacturing and extractive industries, (4) human-oriented services, and (5) other. These were dummy coded with business services used as the reference category in analyses. We also controlled for whether the business returned a *profit* in the last financial year (i.e., pre-pandemic, dummy coded 0 = no, 1 = yes) as a proxy for resource-availability, which has been linked to crisis-preparedness (Williams et al., 2017). In terms of *entrepreneur characteristics*, we include *gender* (women = 1, men = 0) and the respondent’s *age* (in years). *Education* is measured as the highest level of education completed. We included two dummy variables: “secondary education,” coded 1 = secondary (high-school/vocational) and 0 = less than secondary education and “university education,” coded 1 = at least a bachelor’s degree or equivalent and 0 = no university education. Finally, we controlled for the *week* of data collection (i.e., the week in 2020 that the entrepreneurs responded to the survey) to account for dynamics related to the pandemic and government response. Data collection extended over several weeks as we wanted to make sure that entrepreneurs who were harder to reach also had a chance to respond.

#### Robustness check: Trait resilience as an alternative explanation

While our theoretical framework emphasizes agility as a positive adaption to crisis that protects entrepreneur wellbeing, wellbeing in crisis might also be shaped by entrepreneurs’ personality as a resilience promoting capability (Fisher et al., 2019; Williams et al., 2017). As a personality trait, resilience describes “positive psychological capacity to rebound, to ‘bounce back’ from adversity, uncertainty, conflict, failure or even positive change, progress and increased responsibility” (Luthans, 2002, p. 702). Past research finds that entrepreneurs’ trait resilience helps them to navigate the business creation process (Chadwick & Raver, 2020), underpins organizational resilience and firm performance during the COVID-19 pandemic (Anwar et al., 2023), and relates to entrepreneurial intentions in challenging contexts (e.g., war torn countries, Bullough et al., 2014; Renko et al., 2021). Hence in a robustness check, we used *trait resilience* measured with the 6-item resilience scale from the Psychological Capital Questionnaire (Luthans et al., 2006) as an additional control variable.^20^ An example item is “I usually manage difficulties one way or another at work,” which was answered on a 6-point scale ranging from 1 = strongly disagree to 6 = strongly agree. The scale had good reliability (Cronbach Alpha 0.71; Composite Reliability 0.70) and confirmatory factor analyses showed good fit (Satorra–Bentler’s χ^2^ = 64.73, df = 9; CFI = 0.98, GFI = 0.99; AGFI = 0.98; SRMR = 0.03). We replicated our findings controlling for trait resilience.

## Results

Tables 1 and 2 show the descriptive statistics for all variables in our study, including their means, standard deviations, minimum, maximum, and correlations at the individual- and country-level, respectively. We present means and standard deviations based on the raw data. Following Fornell Larcker (1981), we test the convergent and discriminant validity of our measures. The average variance extracted (AVE) from our independent and dependent variables is close or over 0.5, supporting convergent validity. Discriminant validity is also supported because the AVEs are higher than the squared correlations among these constructs (online supplement Table A29). Variance Inflation Factors for the three country-level variables were below 5, indicating no concerns about multicollinearity. The specific VIFs range from 4.0 to 4.4 for GDP, from 3.6 to 3.9 for economic policy response to 2.1 to 2.2 for severity of lockdown.^21^ In one robustness check, we only controlled for GDP and replicated our results (online supplement Table A5).

**Table 2.** Descriptive statistics and correlations on the country level (including country means of individual level variables).

		Mean	SD	Min	Max	1	2	3	4	5	6	7	8	9
1	Severity of lockdown	47.96	7.55	30.00	66.25									
2	GDP ppp pc 2019	34865	19755	4884	65281	−0.53								
3	Government support	0.83	0.36	0.13	1.25	−0.33	0.77							
4	Life satisfaction	6.70	0.59	5.25	7.54	0.16	0.10	0.18						
5	Subjective vitality	3.41	0.25	2.85	3.78	0.66	−0.58	−0.46	0.47					
6	Distress	2.73	0.18	2.33	3.04	−0.13	−0.06	−0.06	−0.52	−0.52				
7	Adverse impact on business	1.25	0.29	0.89	1.90	0.47	−0.43	−0.26	−0.26	0.26	0.05			
8	Opportunity agility	0.40	0.16	0.14	0.68	−0.10	0.27	0.11	0.12	−0.13	0.01	−0.82		
9	Planning agility	4.20	0.70	3.00	5.26	0.39	−0.02	−0.02	0.06	0.24	−0.16	−0.09	0.49	
10	Firm age	10.37	5.57	3.38	28.39	−0.69	0.29	0.17	−0.41	−0.80	0.35	−0.03	−0.24	−0.57
11	Firm size (log of number employees)	1.59	0.50	0.71	2.69	0.53	−0.62	−0.54	0.10	0.43	−0.17	0.24	0.08	0.16
12	Industry: Business services	0.47	0.22	0.06	0.79	−0.24	0.54	0.55	0.38	−0.10	−0.16	−0.77	0.76	0.29
13	Industry: Retail and gastronomy	0.18	0.15	0.02	0.55	0.48	−0.41	−0.31	−0.21	0.13	0.09	0.73	−0.61	−0.03
14	Industry: Manufacturing and extractive	0.16	0.09	0.03	0.43	0.33	−0.76	−0.65	−0.15	0.55	−0.30	0.55	−0.53	−0.18
15	Industry: Human-oriented services	0.12	0.07	0.02	0.31	−0.39	0.48	0.44	−0.07	−0.46	0.50	−0.15	−0.03	−0.08
16	Industry: Other	0.07	0.08	0.00	0.31	−0.24	−0.27	−0.58	−0.43	−0.20	0.12	0.27	−0.32	−0.44
17	Firm profit last year	0.68	0.14	0.42	0.92	0.20	−0.13	0.01	0.51	0.28	−0.16	0.45	−0.50	−0.11
18	Entrepreneur gender	0.31	0.13	0.09	0.62	−0.19	0.34	0.37	0.00	−0.40	0.33	−0.10	−0.01	0.17
19	Entrepreneur age	43.51	6.23	29.15	53.60	−0.61	0.79	0.79	0.17	−0.62	0.05	−0.46	0.20	−0.21
20	Secondary education	0.97	0.03	0.89	1.00	0.42	−0.09	−0.19	0.46	0.47	−0.19	−0.22	0.38	0.27
21	University education	0.73	0.14	0.49	0.94	0.32	−0.12	0.00	0.44	0.53	−0.29	−0.50	0.63	0.49
22	Week of data collection	26.37	4.52	16.92	33.94	0.35	−0.53	−0.40	0.16	0.43	−0.29	−0.10	0.23	0.03
23	Trait resilience	4.51	0.33	3.72	4.93	0.13	0.42	0.51	0.36	0.16	−0.34	−0.41	0.44	0.53
		10	11	12	13	14	15	16	17	18	19	20	21	22
11	Firm size (log of number employees)	−0.37												
12	Industry: Business services	−0.22	−0.12											
13	Industry: Retail and gastronomy	0.06	0.15	−0.85										
14	Industry: Manufacturing and extractive	−0.15	0.47	−0.65	0.41									
15	Industry: Human-oriented services	0.24	−0.75	0.10	−0.09	−0.62								
16	Industry: Other	0.46	0.19	−0.52	0.10	0.43	−0.30							
17	Firm profit last year	0.01	0.14	−0.21	0.29	0.07	−0.02	−0.01						
18	Entrepreneur gender	0.29	−0.59	−0.03	0.25	−0.59	0.71	−0.35	0.15					
19	Entrepreneur age	0.52	−0.59	0.49	−0.37	−0.68	0.47	−0.31	0.02	0.39				
20	Secondary education	−0.46	0.37	0.27	−0.09	−0.01	−0.26	−0.31	−0.06	−0.15	−0.11			
21	University education	−0.60	0.17	0.57	−0.39	−0.18	−0.12	−0.53	−0.20	−0.01	−0.13	0.62		
22	Week of data collection	−0.21	0.58	0.06	−0.08	0.47	−0.68	0.07	−0.04	−0.49	−0.31	0.45	0.39	
23	Trait resilience	−0.52	−0.19	0.64	−0.37	−0.41	0.17	−0.70	−0.13	0.15	0.25	0.22	0.50	−0.06

*Note. N =* 20 countries, except for correlations with subjective vitality (*N =* 18), distress (*N =* 19) and resilience (*N =* 19). Values for variables 4-23 are based on means of country means (thereby giving each country equal weight). They therefore can differ from values in Table 1. Correlations of *r*=0.45 and higher are statistically significant at *p < .*05 level, correlations of *r*=0.60 and higher at *p < .*01.

Table A1 in the online supplement shows descriptive statistics by country. In our sample, the least severe lockdowns were imposed by Japan (score of 30 on the severity of lockdown measure), New Zealand (37), and Poland (41), whereas the most severe lockdown was imposed by China (66). Entrepreneurs’ businesses in China and Bangladesh were the most adversely affected (1.8 and above), whereas entrepreneurs in Poland and Italy reported the least adverse impacts on their business (0.9 and below). Entrepreneurs in Australia and the USA were most agile in recognizing opportunities (64% and above), while those in Bangladesh, France, and Germany were the least agile (19% and below). Entrepreneurs in Chile, Australia, and Brazil were most agile in planning (5.2 and above), and those in Pakistan and Japan were the least agile (3.0 and 3.2). In terms of wellbeing, entrepreneurs in Japan and France experienced the lowest average life satisfaction (5.3 and 5.7 on a 10-point scale), while entrepreneurs in Poland, New Zealand, Bosnia and Herzegovina, Colombia, and Denmark experienced highest average life satisfaction, scoring more than two scale points higher (7.2 and higher). Entrepreneurs in Japan and France also experienced low vitality (2.8 and 3.0, respectively, on a 5-point scale), while entrepreneurs in Colombia and Bangladesh experienced high vitality (3.7 and 3.8). Entrepreneurs in Pakistan and France experienced the highest distress (3.0 on a 5-point scale), while entrepreneurs in Denmark encountered the lowest (2.3).

The entrepreneurs in our study are nested within countries, and we propose indirect effects. Therefore, we test our hypotheses using multi-level generalized structural equation modelling (GSEM using Stata 14). Applying multi-level modelling helps avoid biased standard errors and incorrect estimates (Peterson et al., 2012). The ICCs for the entrepreneur-level variables in our model indicate statically significant country-level variation (all ICCs were significant at *p < .*001 and were 0.075 for life satisfaction, 0.082 for subjective vitality, 0.062 for distress, 0.073 for adverse impact, 0.093 for opportunity agility, and 0.036 for planning agility), supporting the appropriateness of multi-level modelling. All variables except the dummy variables were z-standardized to ease interpretation in multi-level modelling (Hox, 2010).^22^ Because opportunity agility functions as a dependent variable within the GSEM estimation, we specified that part of the GSEM model as a logit regression.

**Figure 2.**
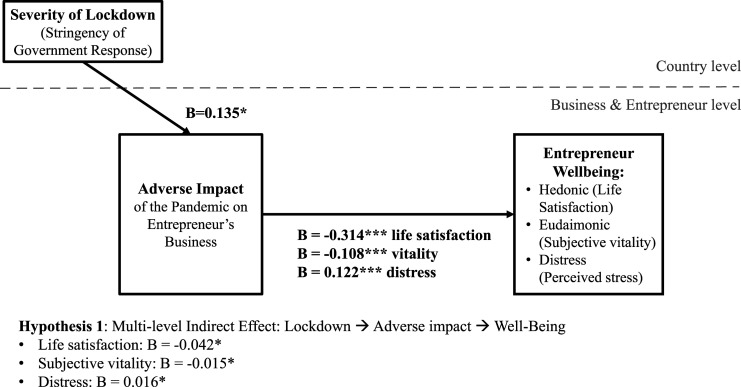
Summary of findings for H1.

**Figure 3.**
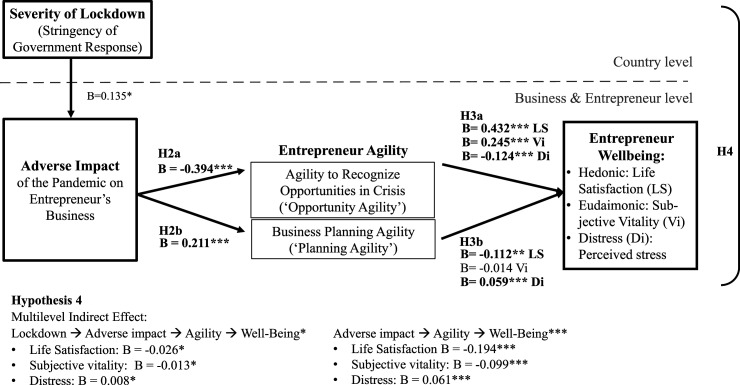
Summary of findings for H2a/b, H3a/b, and H4.

**Figure 4.**
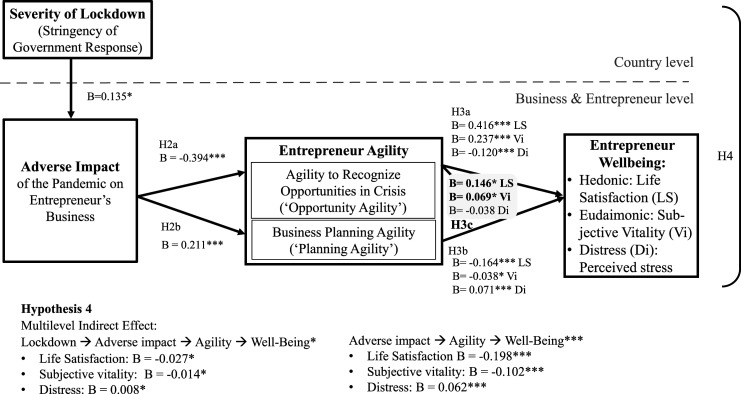
Summary of findings including interaction between opportunity and planning agility H3c.

The results are presented in Tables 3–6. As the GSEM results are lengthy, with one model extending over several columns in each of the tables, we visually summarize the findings in Figures 2–4. Due to space constraints, we do not present control variable-only models.^23^ At the bottom of each table, we report *Pseudo*-*R*^*2*^ (henceforth *R*^*2*^ for short) estimates of explained variance at the individual and country levels and model fit statistics.

**Table 3.** Multi-level estimates for models with life satisfaction as dependent variable.

	Models 1, 2 and 3			Model 1	Model 2	Model 3
	Adverse impact	Opportunity agility (H2a)	Planning agility (H2b)	Life Sat (H1)	Life Sat (H3a, 3b)	Life Sat (H3c)
Column	1	2	3	4	5	6
Severity of Lockdown (H1)	0.135* (0.059)	−0.047 (0.132)	0.059 (0.057)	0.148 (0.132)	0.158 (0.135)	0.159 (0.135)
Adverse impact on business (H1)		−0.394*** (0.041)	0.211*** (0.018)	−0.314*** (0.035)	−0.253*** (0.036)	−0.254*** (0.036)
Opportunity agility (H3a)					0.432*** (0.074)	0.416*** (0.074)
Planning agility (H3b)					−0.112** (0.035)	−0.164*** (0.043)
Planning agility* Opp. agility (H3c)						0.146* (0.070)
Controls						
GDP ppp pc 2019	−0.131 (0.089)	0.363+ (0.197)	0.077 (0.086)	0.094 (0.196)	0.068 (0.201)	0.066 (0.201)
Government support	0.014 (0.079)	−0.164 (0.177)	−0.005 (0.077)	0.090 (0.175)	0.106 (0.179)	0.110 (0.180)
Firm age	0.039+(0.020)	−0.164*** (0.049)	−0.024 (0.020)	−0.031 (0.039)	−0.018 (0.039)	−0.021 (0.039)
Firm size (log employees)	−0.051** (0.020)	0.315*** (0.047)	0.113*** (0.020)	0.136*** (0.039)	0.121** (0.039)	0.121** (0.039)
Industry: Retail and gastronomy	0.119* (0.053)	−0.392** (0.126)	0.011 (0.054)	−0.027 (0.103)	0.010 (0.103)	0.012 (0.103)
Industry: Manufacturing	0.005 (0.054)	−0.554*** (0.126)	0.046 (0.054)	0.105 (0.105)	0.161 (0.104)	0.166 (0.104)
Industry: Human oriented services	0.255*** (0.057)	−0.260+(0.134)	0.016 (0.058)	0.104 (0.112)	0.130 (0.111)	0.127 (0.111)
Industry: Other	0.015 (0.078)	−0.570** (0.192)	−0.118 (0.078)	−0.217 (0.151)	−0.180 (0.151)	−0.186 (0.151)
Firm profit last year	−0.089* (0.040)	−0.203* (0.093)	−0.107** (0.040)	0.176* (0.078)	0.181* (0.078)	0.186* (0.078)
Entrepreneur gender (1=women)	0.043 (0.037)	0.136 (0.087)	0.001 (0.038)	−0.122+(0.073)	−0.134+(0.072)	−0.137+(0.072)
Entrepreneur age	0.035+(0.021)	−0.117* (0.049)	−0.062** (0.021)	0.091* (0.041)	0.095* (0.041)	0.094* (0.041)
Secondary education	−0.070 (0.097)	−0.132 (0.239)	0.048 (0.098)	−0.139 (0.190)	−0.122 (0.189)	−0.104 (0.189)
University education	0.030 (0.042)	0.188+(0.102)	0.062 (0.042)	0.186* (0.082)	0.178* (0.081)	0.175* (0.081)
Week of data collection	−0.073* (0.029)	0.090 (0.066)	−0.070* (0.029)	0.110+(0.057)	0.094+(0.057)	0.099+(0.057)
Constant	−0.017 (0.109)	−0.146 (0.261)	−0.045 (0.109)	6.594*** (0.219)	6.386*** (0.222)	6.361*** (0.222)
N Individuals/Countries	3162/20	3162/20	3162/20	3162/20	3162/20	3162/20
Overall *R*^2^				0.060	0.068	0.069
Individuals/Country *R*^2^				0.040/0.310	0.052/0.270	0.053/0.268
−2 log likelihood				−10758.391	−16987.681	−16985.513

Standard errors in parentheses, *** *p < .*001, ** *p < .*01, * *p < .*05, + *p < .*1, see methods for variable definitions.

**Table 4.** Multi-level estimates for models with subjective vitality as dependent variable.

	Models 4, 5 and 6			Model 4	Model 5	Model 6
	Adverse impact	Opportunity agility (H2a)	Planning agility (H2b)	Vitality (H1)	Vitality (H3a, 3b)	Vitality (H3c)
Column	1	2	3	4	5	6
Severity of lockdown (H1)	0.135* (0.059)	−0.047 (0.132)	0.059 (0.057)	0.122** (0.043)	0.126** (0.046)	0.126** (0.046)
Adverse impact on business (H1)		−0.394*** (0.041)	0.211*** (0.018)	−0.108*** (0.015)	−0.084*** (0.015)	−0.085*** (0.015)
Opportunity agility (H3a)					0.245*** (0.031)	0.237*** (0.031)
Planning agility (H3b)					−0.014 (0.015)	−0.038* (0.018)
Planning agility* Opp. agility (H3c)						0.069* (0.030)
Controls						
GDP ppp pc 2019	−0.131 (0.089)	0.363+(0.197)	0.077 (0.086)	−0.046 (0.067)	−0.065 (0.071)	−0.068 (0.071)
Government support	0.014 (0.079)	−0.164 (0.177)	−0.005 (0.077)	−0.063 (0.057)	−0.053 (0.060)	−0.050 (0.060)
Firm age	0.039+(0.020)	−0.164*** (0.049)	−0.024 (0.020)	−0.036* (0.016)	−0.027+(0.016)	−0.029+(0.016)
Firm size (log employees)	−0.051** (0.020)	0.315*** (0.047)	0.113*** (0.020)	0.078*** (0.016)	0.064*** (0.016)	0.064*** (0.016)
Industry: Retail and gastronomy	0.119* (0.053)	−0.392** (0.126)	0.011 (0.054)	−0.025 (0.043)	−0.007 (0.043)	−0.006 (0.043)
Industry: Manufacturing	0.005 (0.054)	−0.554*** (0.126)	0.046 (0.054)	0.035 (0.043)	0.062 (0.043)	0.065 (0.043)
Industry: Human oriented services	0.255*** (0.057)	−0.260+(0.134)	0.016 (0.058)	−0.029 (0.050)	−0.016 (0.050)	−0.017 (0.050)
Industry: Other	0.015 (0.078)	−0.570** (0.192)	−0.118 (0.078)	−0.017 (0.063)	0.009 (0.062)	0.006 (0.062)
Firm profit last year	−0.089* (0.040)	−0.203* (0.093)	−0.107** (0.040)	−0.017 (0.033)	−0.013 (0.033)	−0.011 (0.033)
Entrepreneur gender (1=women)	0.043 (0.037)	0.136 (0.087)	0.001 (0.038)	−0.117*** (0.031)	−0.122*** (0.031)	−0.124*** (0.031)
Entrepreneur age	0.035+(0.021)	−0.117* (0.049)	−0.062** (0.021)	0.076*** (0.017)	0.080*** (0.017)	0.079*** (0.017)
Secondary education	−0.070 (0.097)	−0.132 (0.239)	0.048 (0.098)	−0.102 (0.083)	−0.090 (0.082)	−0.080 (0.082)
University education	0.030 (0.042)	0.188+(0.102)	0.062 (0.042)	0.023 (0.035)	0.011 (0.035)	0.009 (0.035)
Week of data collection	−0.073* (0.029)	0.090 (0.066)	−0.070* (0.029)	−0.007 (0.023)	−0.011 (0.023)	−0.009 (0.023)
Constant	−0.017 (0.109)	−0.146 (0.261)	−0.045 (0.109)	3.522*** (0.090)	3.406*** (0.091)	3.392*** (0.092)
N Individuals/Countries	3162/20	3162/20	3162/20	2767/18	2767/18	2767/18
Overall *R*^2^				0.093	0.108	0.110
Individuals/Country *R*^2^				0.044/0.644	0.066/0.588	0.068/0.586
−2 log likelihood				−7406.5189	−13623.79	−13621.169

Standard errors in parentheses, *** *p < .*001, ** *p < .*01, * *p < .*05, + *p < .*1, see methods for variable definitions.

**Table 5.** Multi-level estimates for models with distress as dependent variable.

	Models 7, 8 and 9			Model 7	Model 8	Model 9
	Adverse impact	Opportunity agility (H2a)	Planning agility (H2b)	Distress (H1)	Distress (H3a, 3b)	Distress (H3c)
Column	1	2	3	4	5	6
Severity of lockdown (H1)	0.135* (0.059)	−0.047 (0.132)	0.059 (0.057)	−0.065 (0.048)	−0.070 (0.049)	−0.070 (0.049)
Adverse impact on business (H1)		−0.394*** (0.041)	0.211*** (0.018)	0.122*** (0.012)	0.100*** (0.013)	0.100*** (0.013)
Opportunity agility (H3a)					−0.124*** (0.026)	−0.120*** (0.026)
Planning agility (H3b)					0.059*** (0.012)	0.071*** (0.015)
Planning agility* Opp. agility (H3c)						−0.038 (0.025)
Controls						
GDP ppp pc 2019	−0.131 (0.089)	0.363+(0.197)	0.077 (0.086)	−0.039 (0.074)	−0.033 (0.076)	−0.032 (0.076)
Government support	0.014 (0.079)	−0.164 (0.177)	−0.005 (0.077)	0.048 (0.063)	0.042 (0.065)	0.041 (0.064)
Firm age	0.039+(0.020)	−0.164*** (0.049)	−0.024 (0.020)	0.012 (0.014)	0.009 (0.013)	0.010 (0.013)
Firm size (log employees)	−0.051** (0.020)	0.315*** (0.047)	0.113*** (0.020)	−0.051*** (0.013)	−0.049*** (0.014)	−0.049*** (0.014)
Industry: Retail and gastronomy	0.119* (0.053)	−0.392** (0.126)	0.011 (0.054)	0.094** (0.036)	0.084* (0.036)	0.083* (0.036)
Industry: Manufacturing	0.005 (0.054)	−0.554*** (0.126)	0.046 (0.054)	0.065+(0.036)	0.048 (0.036)	0.047 (0.036)
Industry: Human oriented services	0.255*** (0.057)	−0.260+(0.134)	0.016 (0.058)	0.026 (0.040)	0.018 (0.040)	0.019 (0.040)
Industry: Other	0.015 (0.078)	−0.570** (0.192)	−0.118 (0.078)	0.155** (0.052)	0.148** (0.052)	0.150** (0.052)
Firm profit last year	−0.089* (0.040)	−0.203* (0.093)	−0.107** (0.040)	−0.117*** (0.028)	−0.114*** (0.028)	−0.115*** (0.028)
Entrepreneur gender (1=women)	0.043 (0.037)	0.136 (0.087)	0.001 (0.038)	0.125*** (0.026)	0.128*** (0.025)	0.129*** (0.025)
Entrepreneur age	0.035+(0.021)	−0.117* (0.049)	−0.062** (0.021)	−0.115*** (0.015)	−0.114*** (0.014)	−0.114*** (0.014)
Secondary education	−0.070 (0.097)	−0.132 (0.239)	0.048 (0.098)	0.082 (0.065)	0.076 (0.065)	0.071 (0.065)
University education	0.030 (0.042)	0.188+(0.102)	0.062 (0.042)	−0.017 (0.029)	−0.016 (0.028)	−0.015 (0.028)
Week of data collection	−0.073* (0.029)	0.090 (0.066)	−0.070* (0.029)	0.001 (0.020)	0.007 (0.020)	0.006 (0.020)
Constant	−0.017 (0.109)	−0.146 (0.261)	−0.045 (0.109)	2.661*** (0.077)	2.721*** (0.078)	2.728*** (0.078)
N Individuals/Countries	3162/20	3162/20	3162/20	2974/19	2974/19	2974/19
Overall *R*^2^				0.084	0.092	0.093
Individuals/Country *R*^2^				0.082/0.118	0.094/0.055	0.095/0.061
−2 log likelihood				−7175.3641	−13405.096	−13403.943

Standard errors in parentheses, *** *p < .*001, ** *p < .*01, * *p < .*05, + *p < .*1, see methods for variable definitions.

**Table 6.** Summary of indirect effects (H1, H4).

Dependent Variable	Life satisfaction	Subjective vitality	Distress
H1 multi-level indirect effect: Lockdown → Adverse impact → Wellbeing (Models 1, 4 and 7)	*B* = −0.042, *p* = .028, CI: [−0.080, −0.005]	*B* = −0.015, *p* = .030, CI: [−0.028, −0.001]	*B* = 0.016, *p* = .027, CI: [0.002, 0.031]
H4 multi-level indirect effect: Lockdown → Adverse impact → Agility → Wellbeing (Models 3, 6 and 9)	*B* = −0.027, *p* = .036, CI: [−0.052, −0.002]	*B* = −0.014, *p* = .033, CI: [−0.026, −0.001]	*B* = 0.008, *p* = .038, CI: [0.0005, 0.016]
H4 lower-level relationships only: Adverse impact → Agility → Well-Being (Models 3, 6 and 9)	*B* = −0.198, *p* < .001, CI: [−0.269, −0.128]	*B* = −0.102, *p* < .001, CI: [−0.134, −0.069]	*B* = 0.062, *p* < .001, CI: [0.038, 0.086]

*H1* specifies an indirect effect of country-level lockdowns on entrepreneur wellbeing via their adverse impact on entrepreneurs’ businesses. We present information on the relationships underlying this indirect effect in Table 3 (Model 1) for life satisfaction, in Table 4 (Model 4) for subjective vitality, and in Table 5 (Model 7) for distress. Table 6 summarizes the statistical tests of the indirect effect and reports support for *H1*. We find a significant indirect effect for each of our three measures of wellbeing. Specifically, the indirect effect is *B*= −0.042, *p* < .05 for life satisfaction, *B*= −0.015, *p* < .05 for subjective vitality, and *B*= 0.016, *p* < .05 for distress (Table 6). We also find support for each of each of the individual relationships underlying the indirect effect. Entrepreneurs’ businesses are more adversely affected by stronger country-level lockdowns (*B*= 0.135, *p* < .05 in Table 3, Model 1/column 1, Table 4, Model 4/column 1, and Table 5, Model 7/column 1). This effect concerns the first part of the structural equation model, so it is the same for the three wellbeing dependent variables. In turn, when an entrepreneur’s business was more severely impacted, their hedonic and eudaimonic well-being was lower (life satisfaction, Table 3, Model 1/column 4, *B*= −0.314, *p* < .001; subjective vitality, Table 4, Model 4/column 4, *B*= −0.108, *p* < .001), and their distress was higher (Table 5, Model 7/column 4, *B*= 0.122, *p* < .001). Figure 2 summarizes these results.

Regarding the relationship between adverse impact and agility (*H2a* and *H2b*), we found that the adverse impact on the business relates negatively to entrepreneurs’ opportunity agility (*B*= −0.394, *p < .*001, Table 3, Model 2/column 2; Table 4, Model 5/column 2, Table 5, Model 8/column 2), counter to our prediction in *H2a.* However, adverse impact relates positively to planning agility (*B*= 0.211, *p < .*001, Table 3, Model 2/column 3; Table 4, Model 5/column 3, Table 5, Model 8/column 3), which supports H2b. Regarding the relationship between agility and wellbeing (*H3a* and *H3b*), we found that entrepreneurs’ opportunity agility relates positively to their life satisfaction (*B*= 0.432, *p < .*001, Table 3, Model 2/column 5) and their subjective vitality (*B*= 0.245, *p < .*001, Table 4, Model 5/column 5) and relates negatively to distress (*B*= −0.124, *p < .*001, Table 5, Model 8, column 5), supporting *H3a*. Unexpectedly, planning agility relates negatively to entrepreneurs’ life satisfaction (*B*= −0.112, *p < .*01, Table 3, Model 2/column 5), does not relate to subjective vitality (*B*= −0.014, *p* > .1, Table 4, Model 5/column 5), and relates positively to distress (*B*= 0.059, *p < .*001, Table 5, Model 8/column 5), which does not support *H3b*. Figure 3 summarizes these results.

**Figure 5.**
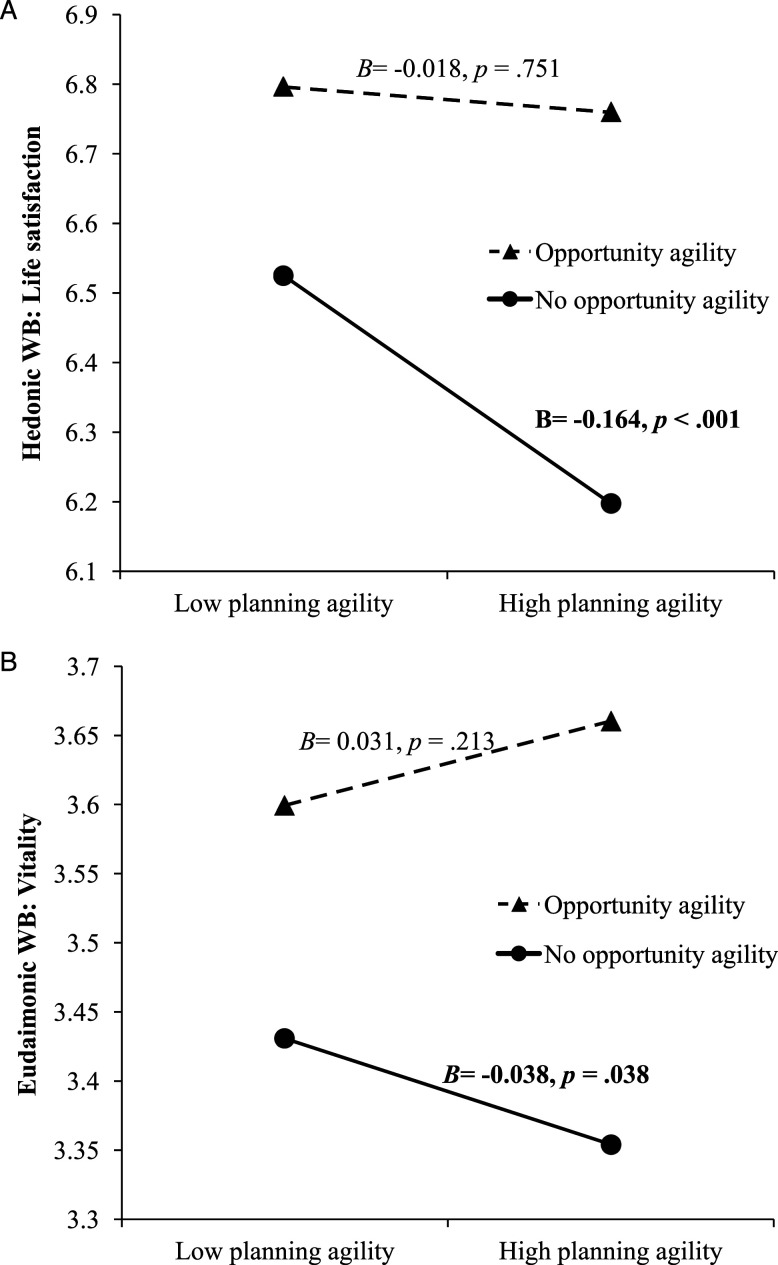
(a) Moderation of opportunity agility on planning agility-life satisfaction relationship; (b) Moderation of opportunity agility on planning agility-subjective vitality relationship.

We found evidence for a positive interactive effect of opportunity agility and planning agility (*H3c*), such that opportunity agility buffers the detrimental effect of planning agility. This interaction is positive and significant for life satisfaction (*B*= 0.146, *p < .*05, Table 3, Model 3/column 6) and subjective vitality (*B*= 0.069, *p < .*05, Table 4, Model 6/column 6). Figure 4 summarizes these results. The interaction effect and results of simple slope tests (Dawson, 2014) are visualized in Figure 5a for life satisfaction and Figure 5b for subjective vitality. When entrepreneurs do not recognize opportunities, engaging in adaptive planning diminishes their life satisfaction and subjective vitality (negative and statistically significant slope tests in Figure 5a and 5b, respectively). The interaction is not significant for distress (*B*= −0.038, *p* >0.1, Table 5, Model 9/column 6).

*H4* proposed that entrepreneurs’ agility mediates the adverse impact of the pandemic on entrepreneur wellbeing. The results depicted in Table 6 support *H4* for life satisfaction (*B*= −0.027, *p < .*05), subjective vitality (*B*= −0.014, *p < .*05) and distress (*B*= 0.008, *p < .*05). The relationships underlying this indirect effect are visualized in Figure 4.

### Robustness tests

**Figure 6.**
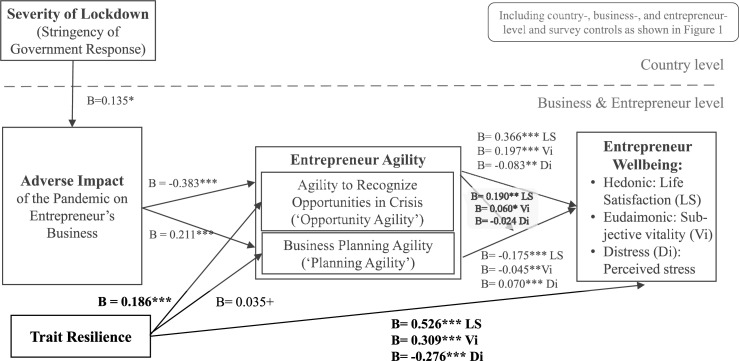
Summary of findings controlling for trait resilience.

We replicated all analyses controlling for entrepreneurs’ trait resilience (Table 7, Figure 6 for a summary). Trait resilience had additive effects and did not change our results. That is, in addition to the effects of adverse impact, trait resilience had a positive effect on opportunity agility and a weak positive effect (*p < .*10) on planning agility, while the effects of adverse impact on the two agility indicators remained significant. Trait resilience had a positive effect on hedonic and eudemonic wellbeing and a negative effect on distress, while the effects of opportunity agility and planning agility remained significant. In further analyses, we tested for interactive effects of adverse impact with trait resilience on agility and wellbeing, respectively. These interactions were not significant (online supplement Table A12).

**Table 7.** Robustness check with controlling for trait resilience.

	Models 10, 11 and 12			Model 10	Model 11	Model 12
	Adverse impact	Opportunity agility	Planning agility	Life satisfaction	Subjective vitality	Distress
Column	1	2	3	4	5	6
Severity of lockdown	0.135* (0.059)	−0.108 (0.141)	0.054 (0.058)	0.017 (0.152)	0.065 (0.044)	−0.011 (0.049)
Adverse impact on business		−0.383*** (0.043)	0.211*** (0.019)	−0.218*** (0.037)	−0.061*** (0.014)	0.079*** (0.012)
Opportunity agility				0.366*** (0.075)	0.197*** (0.029)	−0.083*** (0.024)
Planning agility				−0.175*** (0.042)	−0.045** (0.017)	0.070*** (0.014)
Planning agility* Opp. agility				0.190** (0.071)	0.060* (0.028)	−0.024 (0.023)
Trait resilience		0.186*** (0.046)	0.035+(0.019)	0.526*** (0.036)	0.309*** (0.014)	−0.276*** (0.012)
Controls						
GDP ppp pc 2019	−0.131 (0.089)	0.286 (0.219)	0.069 (0.090)	−0.115 (0.234)	−0.125+(0.068)	0.016 (0.076)
Government support	0.014 (0.079)	−0.227 (0.185)	−0.008 (0.077)	0.091 (0.199)	−0.096+(0.058)	0.079 (0.064)
Firm age	0.039+(0.020)	−0.151** (0.050)	−0.019 (0.021)	0.021 (0.038)	−0.007 (0.015)	−0.009 (0.012)
Firm size (log employees)	−0.051** (0.020)	0.310*** (0.048)	0.108*** (0.021)	0.075+(0.039)	0.044** (0.015)	−0.033** (0.013)
Industry: Retail and gastronomy	0.119* (0.053)	−0.353** (0.130)	0.020 (0.055)	0.059 (0.102)	0.017 (0.040)	0.062+(0.033)
Industry: Manufacturing	0.005 (0.054)	−0.512*** (0.129)	0.071 (0.055)	0.183+(0.103)	0.082* (0.040)	0.042 (0.033)
Industry: Human oriented services	0.255*** (0.057)	−0.171 (0.141)	0.028 (0.061)	0.038 (0.112)	−0.018 (0.047)	0.021 (0.036)
Industry: Other	0.015 (0.078)	−0.552** (0.195)	−0.099 (0.079)	−0.194 (0.146)	0.028 (0.058)	0.140** (0.048)
Firm profit last year	−0.089* (0.040)	−0.173+(0.098)	−0.116** (0.042)	0.109 (0.079)	−0.035 (0.030)	−0.093*** (0.026)
Entrepreneur gender (1=women)	0.043 (0.037)	0.121 (0.091)	−0.006 (0.039)	−0.164* (0.072)	−0.123*** (0.029)	0.133*** (0.023)
Entrepreneur age	0.035+(0.021)	−0.117* (0.052)	−0.067** (0.022)	0.032 (0.041)	0.036* (0.016)	−0.076*** (0.013)
Secondary education	−0.070 (0.097)	−0.078 (0.247)	0.051 (0.100)	−0.102 (0.185)	−0.062 (0.077)	0.043 (0.060)
University education	0.030 (0.042)	0.217* (0.105)	0.069 (0.043)	0.212** (0.080)	0.009 (0.032)	−0.020 (0.026)
Week of data collection	−0.073* (0.029)	0.080 (0.072)	−0.073* (0.030)	0.125* (0.058)	−0.009 (0.022)	0.004 (0.019)
Constant	−0.017 (0.109)	−0.271 (0.271)	−0.060 (0.111)	6.393*** (0.227)	3.406*** (0.086)	2.720*** (0.074)
N Individuals./Countries	3162/20	2935/19	2935/19	2935/19	2723/18	2931/19
Overall *R*^2^				0.120	0.236	0.228
Individuals./Country *R*^2^				0.122/0.097	0.202/0.610	0.240/0.039
- 2 log likelihood				−15941.354	−12886.261	−12634.036

Standard errors in parentheses, *** *p < .*001, ** *p < .*01, * *p* < .05, + *p < .*1, see methods for variable definitions.

We conducted further robustness checks, which are shown in the online supplement where Table A2 provides an overview. Supplemental Tables A3–A20 present results. In total, we conducted 18 different types of robustness checks and tested 350 coefficients that reflect our hypotheses (across three dependent variables). Our results replicate with *the same or stronger significance* levels for all but five of the 350 coefficients. For these five coefficients, significance levels change from *p < .*05 to *p = .*05 or *p = .*06.^24^ These tests relate to country-level variables, applications for government support, and methodological checks.

First, we considered shorter and longer time periods of lockdown stringency and government support (January to July, Supplemental Table A3, and January to September, Table A4). Second, we controlled only for GDP at the country level (Supplemental Table A5). Third, we considered additional effects of the pandemic by expanding the set of country-level controls to include the number of COVID-19 cases per million people and COVID-19 deaths per million people (Supplemental Tables A6 and A7). Fourth, we controlled for whether the entrepreneur applied for government support (Supplemental Table A8). Fifth, we used slightly adjusted measures of adverse impact on entrepreneurs’ businesses (Supplemental Tables A9–A11). Sixth, we considered different sample sizes because the samples for analyses on subjective vitality and distress are more limited. Hence, we limited the sample at all stages of the GSEM analyses and replicated our results (Supplemental Tables A13 and A14). Seventh, we replicated our findings with a limited set of control variables (Supplemental Table A15). Here, we applied country-level control variables for only the first stage of GSEM model (the relationship between country-level lockdowns and adverse impact), firm-level control variables for only the second stage (the effect of adverse impact on agility), and individual-level control variables for the third stage (the effect of agility on wellbeing). We controlled for week of data collection at all stages of the GSEM model. Eighth, we tested the effect of agility on wellbeing only and removed stringency of lockdown and adverse impact (Supplemental Table A16). Ninth, we treated life satisfaction as an ordinal variable and conducted the analysis as logit and probit regression (Supplemental Tables A17 and A18). Tenth, we added the square of the week of data collection to account for a possible non-linear effect (Supplemental Table A19), which was not significant. Finally, we adjusted the week of data collection for the time that had passed since the beginning of the lockdown in that country (Supplemental Table A20), which strengthened the findings supporting H1.

We also conducted longitudinal analyses on a subset of our sample from Poland and Spain to test for possible reverse causality of wellbeing on agility. We used data on pre-crisis wellbeing collected in Jan/Feb 2020 and included our control variables. In line with our theoretical model, adverse impact was a stronger and significant predictor of opportunity agility and planning agility compared to pre-crisis wellbeing (Table A21 of the online supplement).

## Discussion

In virtually all fields of science, COVID-19 has spurred international comparisons. These types of comparisons are difficult to make in research on entrepreneurship because secondary data is not readily available, and collecting data from entrepreneurs is notoriously difficult (cf. Davidsson, 2004). Despite such challenges we were able to collect data from 3162 entrepreneurs across 20 countries and provide a unique snapshot assessment of how these individuals are navigating the COVID-19 pandemic. We found that entrepreneurs experienced different degrees of adversity shaped by the severity of country-level lockdowns and that those hit harder experienced lower hedonic and eudaimonic wellbeing and more distress. By engaging agility (especially opportunity agility on its own and in combination with planning agility), entrepreneurs were able to protect their wellbeing, which supports our hypothesis that the “act” (high agility) approach benefits wellbeing; however, planning agility on its own tended to diminish entrepreneurs’ hedonic wellbeing and increase their distress. Entrepreneurs who adapted their business plans early in the pandemic had poor wellbeing, which indicates that a “wait-and-see” approach can also safeguard wellbeing when no opportunities can be identified. Finally, we found that adversity related positively to planning agility but was negatively related to opportunity agility. Our findings on entrepreneur agility could not be better explained by entrepreneurs’ trait resilience.

### Implications for Research on Entrepreneur Wellbeing

The entrepreneur agility perspective complements existing research on entrepreneur wellbeing (Nikolaev et al., 2020; Shir et al., 2019; Stephan, 2018; Torrès & Thurik, 2019; Wiklund et al., 2019) and research that examines entrepreneur wellbeing in the context of crisis (e.g., Doern, 2016; Patel & Rietveld, 2020; Torrès et al., 2021; Yue & Cowling, 2021) by offering an agentic perspective that highlights crisis as challenges to entrepreneurial agency and agile “entrepreneurial” action as a way to re-assert agency and protect entrepreneur wellbeing in the face of adversity. Integrating entrepreneur agility into research on the wellbeing of entrepreneurs thus starts to unpack what is “entrepreneurial” about entrepreneur wellbeing (e.g., as called for by Wiklund et al., 2019).

The entrepreneur agility perspective frames crises and adversity as challenges to entrepreneurs’ experience of agency and self-determination. Seeing crises this way helps us understand why they have stronger impacts on the wellbeing of entrepreneurs even when entrepreneurs and employees face that same severity of adversity (e.g, Cohidon et al., 2009; Torp et al., 2017), because self-determination is more important for the wellbeing of entrepreneurs than for employees (Shir et al., 2019; Stephan et al., 2020). Viewing crises as challenges to entrepreneurs’ agency thus complements and extends the dominant view of crises as general stressors (e.g., Patel & Rietveld, 2020; Torrès et al., 2021; Wolfe & Patel, 2021; Yue & Cowling, 2021), that is, as demands that require exerting effort and incur suffering. In turn, this implies that successfully navigating crises does not just require working harder (mobilizing effort to deal with the adversity), but also requires entrepreneurs to find ways to reassert agency.

For entrepreneurs, their business is a source of agency and the entrepreneur agility perspective newly links entrepreneurs' actions in business with their wellbeing. In doing so, it introduces a new generative perspective to research on entrepreneur wellbeing in the context of crisis that focuses on actions that entrepreneurs can take to protect their wellbeing (e.g., as called for by Williamson et al., 2021). This generative perspective complements past research which documents how the wellbeing of entrepreneurs suffers in the context of crisis and thus takes a more passive stance (Patel & Rietveld, 2020; Torrès et al., 2021; Wolfe & Patel, 2021; Yue & Cowling, 2021). The agility perspective also expands research on the antecedents of entrepreneur wellbeing in general. These antecedents are typically conceptualized through theories developed for employees or focused on financial stressors (e.g., Gorgievski et al., 2010; Stephan, 2018). The agility perspective expands this by conceptualizing the generative role of entrepreneur’s *actions* in their business for their wellbeing. This responds to calls by Wiklund et al. (2019) and Rauch et al. (2018). It also extends the ideas developed by Williams and Shepherd (2016a, 2016b), who show that starting businesses during a crisis can help victims of disasters cope, to the wellbeing of entrepreneurs leading businesses through crisis.

Regarding the different elements of agility, our findings highlight specifically entrepreneur opportunity agility as the type of agile action that helps entrepreneurs to reassert agency during crisis. Identifying new business opportunities during a crisis gives entrepreneurs new goals to work towards to and thereby a new purpose at a time when uncertainty is high as it was during the COVID-19 pandemic. At such a time, adjusting plans for the business on its own may lead to action without direction and purpose that is insufficient in re-establishing entrepreneurs’ sense of control and agency. Our robustness checks also indicate that the effects of agility cannot be better explained by personality, specifically trait resilience. This result is encouraging because it means that even entrepreneurs who do not have a resilient personality can protect their wellbeing during a crisis by engaging (opportunity) agility.

We also enrich existing accounts of entrepreneur wellbeing in the context of crisis (e.g., Torrès et al., 2021; Wolfe & Patel, 2021; Yue & Cowling, 2021). First, instead of inferring crisis from an entrepreneurs’ context as past research has done, our multi-level cross-country design allowed us to measure the severity of the crisis and assess entrepreneurs experience of crisis-induced disruption and adversity (as called for by resilience researchers Fisher et al., 2019; Williams et al., 2017) which varied substantially for entrepreneurs located in the same crisis context. Second, we complement the focus on negative wellbeing in this line of research (e.g., poor mental health or burnout Cohidon et al., 2009; Torrès et al., 2021) by showing that adversity also diminishes entrepreneurs’ positive hedonic and eudaimonic wellbeing. This is not self-evident because negative and positive wellbeing are not simply opposites but can vary independently and often have different antecedents (Stephan et al., 2022). Our research suggests that adversity is a shared antecedent of both forms of wellbeing.

### Entrepreneur Agility: Implications for Research on Entrepreneurship and Crisis

Prior studies have identified a number of different ways in which entrepreneurs can respond to crises (e.g., Giones et al., 2020; Kuckertz et al., 2020; Manolova et al., 2020; Shepherd, 2020). We contribute to this literature by conceptualizing and organizing these responses as aspects of entrepreneur agility as a specific in-crisis resilience mechanism. By investigating how entrepreneur agility impacts wellbeing and is activated by adversity across many entrepreneurs and countries, we offer new insights into resilient responses to crisis.

First, by linking entrepreneur agility and wellbeing, our study extends entrepreneurship research on crises and resilience that is focused on the relationship between crisis-responses and business survival and performance (Giones et al., 2020; Manolova et al., 2020; Shepherd, 2020), entrepreneurial intentions (Bergenholtz et al., 2023; Bullough et al., 2014; Renko et al., 2021), and start-up efforts (Davidsson & Gordon, 2016; Williams & Shepherd, 2016a,b) by proposing entrepreneur wellbeing as an important microlevel outcome. While past research suggests that agile responses have positive effects on business performance (Doz & Kosonen, 2008; Kuckertz et al., 2020; Manolova et al., 2020; Reymen et al., 2015; Shams et al., 2020; Weber & Tarba, 2014), our research presents more nuanced evidence of how these responses affect business leaders themselves. We also believe that our findings are relevant to related research on effectuation, improvisation, and bricolage (Fisher, 2012; Hmieleski & Corbett, 2008; Sarasvathy, 2001), which like agility also considers flexibility and adaptability, but which so far has not considered their effect on the personal wellbeing of the entrepreneur.

Specifically, our findings on planning agility suggest that there can be wellbeing benefits to embracing a “wait-and-see” approach that delays agile adaptive planning. Adapting plans requires effort and changing plans too early may increase uncertainty during a crisis. In this respect, our results suggest that in a crisis situation, it can be beneficial to wait and develop a better understanding how the crisis will evolve or until opportunities can be identified. These effects may be linked to the severity of the COVID-19 crisis, which was associated with high prolonged levels of uncertainty (e.g., about the virus and its mutation, and acceptable containment measures). We hope future research can determine what the “optimum” wait-and-see time might be for different degrees of adversity. Again, while adaptive and flexible planning in response to uncertainty (Brinckmann et al., 2010; Giones et al., 2020) benefits business performance, our findings encourage researchers to consider the potential personal wellbeing costs to entrepreneurs facing a crisis. If agile adaptive planning approaches deplete the wellbeing of entrepreneurs, they may put the business at risk. However, when paired with opportunity recognition, planning agility can create a sense of control and alleviate uncertainty by allowing entrepreneurs to structure a situation and set goals (Gielnik et al., 2015; Gollwitzer, 1996; Kush & Cochran, 1993). Thus, the wellbeing benefits derived from combining opportunity agility and planning agility align with research suggesting that “act” approaches can enhance business survival and performance (e.g., Giones et al., 2020), and indicate that what is good for the wellbeing of the entrepreneur is also good for their business.

Second, our research expands and nuances understandings about how adversity can activate agility and entrepreneurial responses to crises. We found that adversity had an activating effect on planning agility, which aligns with research on resilient responses to adversity (e.g., Sutcliffe & Vogus, 2003), entrepreneurial responses to crises (e.g., Giones et al., 2020; Kuckertz et al., 2020; Manolova et al., 2020; Shepherd, 2020), and agility in strategic management (e.g., Doz & Kosonen, 2008; Shams et al., 2020; Weber & Tarba, 2014); however, we found that adversity ultimately reduces opportunity agility. This latter finding is consistent with threat-rigidity theory (Staw et al., 1981) and goes against what most research argues about entrepreneurial responses that take place during a crisis (e.g., Kuckertz et al., 2020; Shepherd, 2020). More recent research that focuses on environmental enablers similarly casts environmental changes, including crises, as sources of business opportunities (Kimjeon & Davidsson, 2021). Our finding may reflect the greater opportunity costs associated with opportunity (vs. planning) agility.

Specifically, compared with adaptive planning, opportunity agility requires outward exploration and implies more substantive changes to the business. Making these types of changes can be more difficult for the entrepreneurs who took part in our study, who lead more established businesses (average age 10 years) that have established routines, especially compared to the start-ups and new businesses that appear in research on entrepreneurship and crisis and external enablers (Kimjeon & Davidsson, 2021; Kuckertz et al., 2020). Moreover, compared to the large firms considered in the strategy literature on agility (e.g., Doz & Kosonen, 2008), entrepreneurs are more resource constrained, which leaves them with less capacity to look outward for new opportunities when the survival of their business is at stake. Their personal opportunity cost is likely high as well because their livelihood and the livelihoods of their employees are at stake, which might make them more cautious and “rigid” about making substantial changes. In summary, our findings suggest that opportunity agility is not automatically activated by adversity, but instead needs to be actively supported and developed, especially during a severe crisis. Future research could develop more refined models that consider different aspects of agility separately, confirm boundary conditions, and extend our research to long-term outcomes and post-crisis performance.

### A Unique International Comparison offering Insights into Context

We believe that research making international comparisons is here to stay also in the field of entrepreneurship. The fact that international comparative studies like ours are scarce is surprising given that the two best known international efforts in the field (GEM and PSED) have been remarkably successful (Bergmann et al., 2013; Bosma, 2013; Davidsson, 2016). Our focus was on the wellbeing of practicing entrepreneurs, which is an important outcome because many entrepreneurs start their businesses to self-actualize and are not primarily interested in achieving high performance (cf. Wiklund et al., 2019). Importantly, focusing on wellbeing facilitated our international comparison because we could rely on internationally validated instruments, and the measure is not dependent on national reporting differences of individual or business financials. Unlike general cross-national datasets (e.g., World Values Survey), our data can uniquely shed light on what is entrepreneurship-specific about entrepreneur wellbeing.

Our study also broadens general research on entrepreneur wellbeing, which typically does not consider country context (Stephan, 2018; Wiklund et al., 2019)^25^ and it complements conceptual accounts of the agility of large firms across countries (Gomes et al., 2020; Shams et al., 2020). We offer insight into how a newly devised formal institution (lockdown regulations introduced in response to the COVID-19 crisis) can constrain and enable entrepreneur agility and impact wellbeing. Establishing links between crisis, adversity, agility, and three types of wellbeing, in a sample of countries that represent 73% of the world’s GDP and are home to 56% of the world’s population contributes to the generalization of our results. We hope our study inspires more international comparisons of entrepreneur wellbeing.

### Limitations and Future Research

Like all studies, ours has limitations that also provide opportunities for future research. First, to gather data of acceptable samples size from multiple countries, we had to use short versions of scales and single-item measures; however, we made sure that when we used single items, they were either validated (e.g., for life satisfaction) or our measures (e.g., planning agility) asked for factual information where concern about reliability and bias are less relevant (Bergkvist & Rossiter, 2007; Wanous & Hudy, 2001). Nevertheless, we hope future research can use more refined measures, especially for agility (opportunity agility and planning agility).

Second, our focus was on understanding entrepreneurs’ actions and experiences while the crisis was unfolding. There was some variation with how the pandemic started and the types of lockdowns that were implemented across countries, which is why we collected data from April to early September and why we controlled for the precise week of data collection for each respondent in our study. Future research can examine persistence throughout the pandemic and its different stages as well as long-term outcomes post-crisis. For instance, how might entrepreneurs learn from their agility? Can early agility make them and their businesses more resilient during future crises? The two different crisis responses (“act” or “wait-and-see”) could be explored in more detail and depth (e.g., qualitatively) over different time periods.

Third, we assessed the adverse impact of the pandemic on entrepreneurs’ businesses through reduction in sales/trading and perceived threat to survival. These measures capture the *immediate* impact of the pandemic when redundancies or business closures have not yet occurred. Many entrepreneurs delay business closure and laying off staff for personal reasons (e.g., Shepherd et al., 2009), and these actions are also confounded with government policies and support programs for entrepreneurs (e.g., COVID-loans, grants, tax relief, subsidies of employee wages). We thus believe that our measure of the adverse impact of the pandemic has the advantage of offering an immediate assessment that allows us to understand how entrepreneurs react to an ongoing crisis. Future research could develop and validate alternative operationalizations of adverse impact and relate them to post-crises outcomes.

Fourth, while we offer information on how our sample compares to representative household samples of entrepreneurs in GEM, we did not employ a representative sampling frame. This approach is difficult in cross-country research on entrepreneurship, especially as we were interested in operating businesses because formal business registrations vary substantially across countries. Another bias could be that some entrepreneurs may have already closed their businesses or went bankrupt. Our decision to collect data during the first phase of the pandemic likely helps mitigate this bias, as does the fact that 150 entrepreneurs in our sample stated that they had “to suspend trading and close [their] business” in the last month.

Fifth, while we proposed theoretical arguments for a particular causal direction, accounted for alternative explanations through control variables, and replicated findings in extensive robustness checks, our data are cross-sectional. There is thus a possibility for reverse causality such that high wellbeing may lead entrepreneurs to see more opportunities. To address this concern, we analyzed longitudinal data for two of our 20 countries for which we had collected pre-crisis data on wellbeing: Poland and Spain (available in Table A21 the online supplement). These findings support our hypothesized effects and alleviate concerns about reverse causality. Adverse impact was a stronger predictor of opportunity agility and planning agility than pre-crisis wellbeing. Longitudinal research of entrepreneurs across 20 countries is unfortunately not yet feasible due to the resources involved, but we hope it will be in the future.

Sixth, while we have emphasized agility, there are other important drivers of entrepreneur wellbeing during a crisis. We considered entrepreneurs’ applying for government support in robustness checks. Beyond that, Doern’s (2016, 2017) qualitative research highlights that mobilizing social support and reframing can help entrepreneurs re-establish wellbeing after a crisis, while Williamson et al. (2021) call for entrepreneurs to make time for recovery and self-care during stressful times (also see Wach et al., 2021). We encourage future research that considers these different strategies in tandem to discern their relative importance for entrepreneur wellbeing and potential synergistic effects among strategies. Future research would also benefit from directly assessing entrepreneurs’ sense of agency, for example, though measures of psychological functioning (e.g., Ryff, 2019; Shir & Ryff, 2021).

Finally, the COVID-19 pandemic differs from recent economic crises (e.g., the 2008 financial crisis) in that it poses a greater threat to health, high mortality rates, and changes to social interaction patterns due social distancing and remote work. However, historically, there have been similar crisis (e.g., the Spanish Flu pandemic 1918-1920), and there will likely be more pandemics in the future (Mithani, 2020; Phan, 2021; Phan & Wood, 2020). Thus, while our findings are specific to the context of one crisis, they have implications for future crises as well.

### Practical Implications

First, while agility is activated by adversity and trait resilience, it is ultimately a volitional action that entrepreneurs have control over and that can likely be supported through practical intervention. Our findings suggest that entrepreneurs should be aware of the various types of agility and the different ways that they are triggered by adversity. They also show that entrepreneurs should be encouraged to be agile in terms of recognizing new business opportunities during a crisis, as this will benefit their wellbeing. At the same time, recognizing new business opportunities becomes increasingly difficult the more severe a crisis/adverse situation becomes. Our findings indicate that in more adverse situations, entrepreneurs will be inclined to use planning agility, but they should recognize that engaging planning agility alone (i.e., without also identifying new opportunities) will drain their wellbeing and lead to stress. To counteract this type of stress, entrepreneurs may benefit from utilizing short-term stress alleviation techniques, such as emotion-focused coping and detachment (Patzelt & Shepherd, 2011; Wach et al., 2021) as well as coaching interventions (Schermuly et al., 2021) or cognitive behavior therapy intervention (Williamson et al., 2021). In summary, entrepreneurs should understand that a “wait-and-see” strategy could benefit their wellbeing if they do not recognize new opportunities. Conversely, acting is the best strategy for both wellbeing and business outcomes (Giones et al., 2020; Kuckertz et al., 2020) when an opportunity can be identified.

Second, our findings could also inform the actions of policy makers and entrepreneur support organizations. For instance, given the important role of opportunity agility, policy makers could implement incentives in the form of COVID-opportunity vouchers building on the success of innovation voucher schemes (Roper, 2018). These vouchers offer financial support to pay for advice and run small-scale experiments that explore the potential of new business opportunities for income generation. Entrepreneur support organizations could offer idea generation workshops or online tools and create networking opportunities to aid opportunity recognition. Networking may also help activate new sources of social support for entrepreneurs that would strengthen their wellbeing.

Third, we hope that our findings about the impact that crises have on the wellbeing of entrepreneurs will encourage policy makers to help de-stigmatize this important topic. Entrepreneurs are all too often depicted as heroic and successful (Suàrez et al., 2020), and a more honest conversation about wellbeing will help encourage them to look after their own mental health. This will also help investors safeguard the investments that they make in these entrepreneurs and ultimately benefit the economy by enhancing entrepreneur productivity (e.g., Stephan, 2018).

## Supplemental Material

sj-pdf-1-etp-10.1177_10422587221104820 – Supplemental Material for Act or Wait-and-See? Adversity, Agility, and Entrepreneur Wellbeing across Countries during the COVID-19 Pandemic Click here for additional data file. Supplemental Material, sj-pdf-1-etp-10.1177_10422587221104820 for Act or Wait-and-See? Adversity, Agility, and Entrepreneur Wellbeing across Countries during the COVID-19 Pandemic by Ute Stephan, Przemysław Zbierowski, Ana Pérez-Luño¸ Dominika Wach, Johan Wiklund, Marisleidy Alba Cabañas, Edgard Barki, Alexandre Benzari, Claudia Bernhard-Oettel, Janet A. Boekhorst, Arobindu Dash, Adnan Efendic, Constanze Eib, Pierre-Jean Hanard, Tatiana Iakovleva, Satoshi Kawakatsu, Saddam Khalid, Michael Leatherbee, Jun Li, Sharon Parker, Jingjing Qu, Francesco Rosati, Sreevas Sahasranamam, Marcus A. Y. Salusse, Tomoki Sekiguchi, Nicola Thomas, Olivier Torrès, Mi H. Tran, Mary Katherine Ward, Amanda Williamson and Muhammad Mohsin Zahid in Entrepreneurship Theory and Practice

## ORCID iDs

Ute Stephan https://orcid.org/0000-0003-4514-6057

Przemysław Zbierowski https://orcid.org/0000-0001-6144-1940

Ana Pérez-Luño https://orcid.org/0000-0002-9210-5269

Dominika Wach https://orcid.org/0000-0001-6344-9508

Johan Wiklund https://orcid.org/0000-0002-1105-2469

Alexandre Benzari https://orcid.org/0000-0002-6853-1225

Janet A. Boekhorst https://orcid.org/0000-0001-5294-6787

Arobindu Dash https://orcid.org/0000-0003-4642-512X

Michael Leatherbee https://orcid.org/0000-0002-9125-8271

Jun Li https://orcid.org/0000-0002-6737-2728

Jingjing Qu https://orcid.org/0000-0002-8510-433X

Francesco Rosati https://orcid.org/0000-0001-5085-5056

Sreevas Sahasranamam https://orcid.org/0000-0001-9418-4493

Marcus A. Y. Salusse https://orcid.org/0000-0001-8338-000X

Tomoki Sekiguchi https://orcid.org/0000-0003-4522-5249

Olivier Torrès https://orcid.org/0000-0002-7104-0486

Mi Hoang Tran https://orcid.org/0000-0003-1359-7339

M.K. Ward https://orcid.org/0000-0002-1253-1744

Amanda Williamson https://orcid.org/0000-0003-3727-4577

Muhammad Mohsin Zahid https://orcid.org/0000-0002-9255-1787

## Notes

1. As of March 2022, https://coronavirus.jhu.edu/map.html and Economist (2022) Excess death estimates.

2. We adopt a broad occupational definition of entrepreneurs as is common in research on entrepreneurs’ wellbeing (Stephan, 2018). We detail our sample selection criteria in the Methods section.

3. The only qualitative studies–of 15 UK entrepreneurs after the 2011 London Riots (Doern, 2016, 2017) and eight UK entrepreneurs during COVID-19 (Doern, 2021)—focused on entrepreneurs’ crisis response in general. They document entrepreneurs’ emotional stress and efforts to cope with the psychological strain of the crisis.

4. An exception is Williams and Shepherd (2016a), who examine start-ups that were created by crisis victims after a disaster.

5. We include two further elements of resilience (Fisher et al., 2019; Williams et al., 2017) in our study: severity of adversity (the resilience “trigger”) and resilience promoting capabilities (trait resilience robustness check).

6. Psychological functioning is another aspect of eudaimonic wellbeing (Nikolaev et al., 2020; Ryff, 2019; Shir & Ryff, 2021). Psychological functioning can be understood as a predictor of wellbeing outcomes (Diener et al., 2018), including hedonic wellbeing (Nikolaev et al., 2020) and subjective vitality (Shir et al., 2019; Stephan et al., 2020). In this paper, we examine wellbeing outcomes empirically and, in our theorizing, consider experienced agency and autonomy as a core element of psychological functioning (Shir et al., 2019).

7. We account for other aspect of crisis severity in our robustness checks (e.g., number of deaths).

8. Of course, this does not preclude that *some* entrepreneurs’ businesses may also thrive in lockdowns.

9. There is much research on supply chain agility (Gligor et al., 2015; Sherehiy et al., 2007; Swafford et al., 2006), and information systems research considers digital capacities and information technology as sources of organizational agility (Sambamurthy et al., 2003; Tallon et al., 2019).

10. Specifically, Laskovaia et al., (2019) found that flexible planning and experimenting (effectuation) was ineffective in boosting firm performance for entrepreneurs during the 2014–16 economic crisis in Russia.

11. Our study included Australia, Bangladesh, Bosnia and Herzegovina, Brazil, Canada, Chile, China, Colombia, Denmark, France, Germany, India, Italy, Japan, New Zealand, Pakistan, Poland, Spain, UK, USA. We also collected data in Sweden and Norway, but could not include these countries in our results due to missing variables

12.
https://coronavirus.jhu.edu/data/mortality

13. Source: World Bank, World Development Indicators, GDP in current US$ in 2019, population in 2019.

14. Such as insights into the challenges that entrepreneurs were facing (for support organization sand banks) and free personalized feedback on personal resilience and coping skills for the entrepreneurs.

15. We excluded 604 participants who declared that they were not entrepreneurs (i.e., neither self-employed, start-up entrepreneurs, or small- and medium-sized business owners, or a combination of these). Being an entrepreneur implies ownership and control, thus we also removed participants who did not own the business and who were *neither* the CEOs *nor* managed their business on a daily basis (*N* =1058). To ensure comparability across countries, we limited the sample to businesses with a maximum of 250 employees, in line with the EU definition of small- and medium-sized businesses (excluding 40 responses). Finally, 42 survey participants were excluded because they did not give consent to use their data.

16. The sample was reduced to 2767 for subjective vitality and 2974 for distress as dependent variables as they were not included in the survey for Germany and the USA (subjective vitality) and USA (distress).

17. Firms in our sample were on average 9.98 years old with a range from 0 years (newly created firms) to 81 years. Most firms were 6 years old or younger (51%) and five percent of firms were 30 years or older. While most firms in our sample were created by the entrepreneurs we surveyed (84.4%), our sample also included some family businesses and businesses that were bought and thus of older age. In all cases, the surveyed entrepreneur was owning and managing the business on a daily basis or acting as CEO.

18. Data comes from WVS round 5 and 6 covering 19 countries. No data was available for Bosnia Herzegovina.

19. Including school closing, workplace closing, cancelling public events, restrictions on private gathering, closing of public transportation, “stay at home” requirements, restrictions on internal movement and on international travel. Each indicator is rated for degree. For instance, the extent to which workplaces have been closed is assessed as “0 - no measures, 1 - recommend closing (or recommend work from home), 2 - require closing (or work from home) for some sectors or categories of workers, and 3 - require closing (or work from home) for all-but-essential workplaces. See (Hale et al. (2021) for more detail.

20. These analyses are based on 19 countries for life satisfaction and distress (18 countries for vitality) as data on trait resilience was not available for the USA.

21. The range in VIF scores is due to variation in sample size of countries and individuals for the three dependent variables as described in the method section.

22. We standardized country-level variables based on their country-level means and standard deviations. Lower-level variables are z-standardized (i.e., grand-mean centered).

23. The *R*^*2*^ for models that included only control variables were: 0.035 for adverse impact, 0.053 for opportunity agility, 0.024 for planning agility, 0.033 for life satisfaction, 0.063 for subjective vitality 0.057 for distress.

24. Specifically, when we aggregate the stringency of the government response over nine instead of 8 months, the significance of the indirect effect weakens to *p = .*06 instead of *p = .*04. When we use a limited set of control variables and when we treat life satisfaction as ordinal variable and estimate probit regression, the interaction between opportunity agility and planning agility (H3c) weakens to *p = .*051 instead of *p < .*05.

25. The few exceptions investigate how institutions and culture impact entrepreneur wellbeing across European countries (Fritsch et al., 2019; Stephan et al., 2020; Wolfe & Patel, 2018), in a meta-analysis (Stephan et al., 2022) or document descriptive differences between entrepreneurs and employees (Larsson & Thulin, 2019).
