# Supplementary material for: Act or Wait-and-See? Adversity, Agility, and Entrepreneur Wellbeing across Countries during the COVID-19 Pandemic
Source: Entrep Theory Pract. 2022 Jun 9;47(3):682–723. doi: 10.1177/10422587221104820 (PMC9184834; doi:10.1177/10422587221104820)
Supplement: sj-pdf-1-etp-10.1177_10422587221104820 – Supplemental Material for Act or Wait-and-See? Adversity, Agility, and Entrepreneur Wellbeing across Countries during the COVID-19 Pandemic [file sj-pdf-1-etp-10.1177_10422587221104820.pdf]

Online Supplement

For

Stephan, U., Zbierowski, P., Pérez-Luño, A., Wach, D., Wiklund, J., Alba Cabañas, M., Barki, E., Benzari, A., Bernhard-Oettel, C., Boekhorst, J.A., Dash, A., Efendic, A., Eib, C., Hanard, P.-J., Iakovleva, T., Kawakatsu, S., Khalid, S., Leatherbee, M., Li, J., Parker, S., Qu, J., Rosati, F., Sahasranamam, S., Sekiguchi, T., Salusse, M.A.Y., Thomas, N., Torres, O., Tran, M.H., Ward, M.K., Williamson, A. & Zahid, M.
Act or Wait-and-See? Adversity, Agility, and Entrepreneur Wellbeing across Countries during the COVID-19 Pandemic. *Entrepreneurship Theory & Practice*

For Review Only

Table of contents

Descriptive statistics: Country characteristics and country means 2
Summary Robustness Checks 4
Overview Table Robustness Checks 6
Sample Comparisons & Representativeness against GEM entrepreneurs 28
Convergent and discriminant validity 38

Descriptive statistics: Country characteristics and country means

Table A1. Descriptive Statistics: Country Characteristics and Country Means and SD of Entrepreneur/Firm Characteristics by Country

		Country characteristics			Country Means of Entrepreneur/Firm Characteristics								
	N (%)	Severity of lockdown	GDP pc 2019	Government support	Life satisfaction *Mean (SD)*	Vitality *Mean (SD)*	Distress *Mean (SD)*	Adverse impact *Mean (SD)*	Opportunity agility *N (%)*	Planning agility *Mean (SD)*	Firm age *Mean (SD)*	Firm size *Mean (SD)*	Industry: business services *N (%)*
Australia	88	48.75	53320	0.63	6.59	3.38	2.83	1.00	60	5.25	5.98	9.32	56
					(2.16)	(0.88)	(0.75)	(1.19)	(68.2)	(3.13)	(4.25)	(18.15)	(63.6)
Bangladesh	81	52.88	4950	0.13	6.15	3.78	2.54	1.77	11	4.65	5.43	24.59	5
					(2.61)	(0.53)	(0.67)	(0.62)	(13.6)	(2.90)	(7.94)	(58.13)	(6.2)
Bosnia -Herzegovina	89	46.63	15792	0.50	7.40	3.57	2.68	1.42	25	3.53	13.64	23.78	35
					(1.93)	(0.56)	(0.57)	(0.93)	(28.1)	(3.08)	(10.95)	(47.32)	(39.3)
Brazil	154	50.50	15259	0.63	6.76	3.35	2.95	1.17	73	5.26	11.08	6.05	56
					(1.84)	(0.87)	(0.63)	(1.09)	(47.4)	(2.79)	(12.29)	(14.65)	(36.4)
Canada	276	44.88	51342	1.25	6.91	3.20	2.76	1.06	134	4.53	12.68	9.28	194
					(1.56)	(0.82)	(0.70)	(1.12)	(48.6)	(3.02)	(12.65)	(18.59)	(70.3)
Chile	130	50.25	25155	0.88	5.98	3.57	2.63	1.23	70	5.17	3.38	7.51	78
					(1.96)	(0.71)	(0.60)	(1.07)	(53.8)	(2.90)	(7.68)	(15.59)	(60.0)
China	469	66.25	16785	0.50	6.87	3.64	2.56	1.90	113	4.81	5.79	30.65	60
					(1.66)	(0.59)	(0.56)	(0.44)	(24.1)	(3.23)	(4.50)	(46.82)	(12.8)
Colombia	84	57.00	15644	0.63	7.42	3.73	2.59	1.20	37	4.14	7.07	10.27	31
					(1.72)	(0.67)	(0.54)	(1.13)	(44.0)	(2.57)	(7.21)	(21.13)	(36.9)
Denmark	134	43.63	59830	1.25	7.54	3.58	2.33	1.08	44	3.25	11.66	6.37	84
					(1.79)	(0.79)	(0.69)	(0.94)	(32.8)	(3.47)	(9.29)	(17.77)	(62.7)
France	111	48.50	49435	1.25	5.73	3.02	3.04	1.59	21	3.47	17.88	6.76	21
					(2.00)	(0.91)	(0.72)	(0.68)	(18.9)	(2.89)	(19.84)	(16.78)	(18.9)
Germany	211	43.38	56052	1.25	6.70		2.92	1.64	35	3.87	12.25	4.79	97
					(1.93)		(0.75)	(0.64)	(16.6)	(3.51)	(7.84)	(19.55)	(46.0)
India	97	56.00	7034	0.63	6.29	3.61	2.88	1.13	56	4.16	6.45	21.36	66
					(2.21)	(0.72)	(0.68)	(1.07)	(57.7)	(2.84)	(9.46)	(29.77)	(68.0)
Italy	135	49.63	44197	0.63	7.02	3.44	2.68	0.91	76	4.21	6.73	7.37	84
					(1.66)	(0.78)	(0.63)	(1.19)	(56.3)	(3.22)	(9.99)	(20.39)	(62.2)
Japan	71	30.00	43236	0.50	5.25	2.85	2.90	1.13	31	3.15	28.39	19.56	19
					(2.28)	(0.57)	(0.42)	(0.96)	(43.7)	(3.57)	(23.43)	(71.89)	(26.8)
New Zealand	118	36.75	43953	1.25	7.20	3.23	2.54	1.06	54	4.44	11.41	10.17	70
					(1.84)	(0.81)	(0.62)	(1.12)	(45.8)	(3.10)	(12.30)	(14.14)	(59.3)
Pakistan	93	51.25	4884	0.50	6.63	3.39	2.97	1.47	23	3.00	8.40	22.68	23
					(2.32)	(0.66)	(0.54)	(0.98)	(24.7)	(3.01)	(9.22)	(46.42)	(24.7)
Poland	85	40.75	34218	0.63	7.19	3.49	2.78	0.89	36	3.76	9.67	9.08	46
					(1.71)	(0.70)	(0.65)	(1.08)	(42.4)	(3.28)	(7.87)	(20.24)	(54.1)
Spain	215	46.88	42214	1.13	6.46	3.37	2.70	1.21	66	3.86	12.23	2.58	82
					(1.98)	(0.72)	(0.63)	(1.09)	(30.7)	(3.20)	(11.86)	(5.86)	(38.1)
UK	374	47.75	48710	1.25	6.82	3.17	2.66	1.15	180	4.74	11.60	10.60	294
					(1.85)	(0.90)	(0.77)	(1.05)	(48.1)	(3.14)	(10.04)	(21.71)	(78.6)
USA	147	47.63	65281	1.25	7.13			0.97	94	4.73	5.69	9.61	114
					(1.86)			(1.11)	(63.9)	(3.35)	(3.51)	(27.66)	(77.6)

For Review Only

Table A1 continued. Descriptive Statistics: Country Characteristics and Country Means and SD of Entrepreneur/Firm Characteristics by Country

	N (%)	Industry: retail & gastronomy, *N (%)*	Industry: manufacturing and extractive, *N (%)*	Industry: human-oriented ervices, *N (%)*	Industry: other, *N (%)*	Firm profit last year *N (%)*	Entrepreneur gender *N (% women)*	Entrepreneur age *Mean (SD)*	Secondary education *N (%)*	University education *N (%)*	Week *Mean (SD)*	Trait resilience *Mean (SD)*
Australia	88	8 (9.1)	5 (5.7)	14 (15.9)	5 (5.7)	55 (62.5)	25 (28.4)	46.45 (10.27)	87 (98.9)	62 (70.5)	25.19 (5.01)	4.93 (0.57)
Bangladesh	81	24 (29.6)	35 (43.2)	2 (2.5)	15 (18.5)	53 (65.4)	7 (8.6)	29.15 (7.65)	77 (95.1)	51 (63.0)	25.43 (2.93)	4.43 (0.62)
Bosnia -Herzegovina	89	18 (20.2)	24 (27.0)	3 (3.4)	9 (10.1)	82 (92.1)	15 (16.9)	45.28 (10.11)	89 (100.0)	59 (66.3)	32.88 (3.41)	3.83 (0.40)
Brazil	154	42 (27.3)	16 (10.4)	35 (22.7)	5 (3.2)	109 (70.8)	96 (62.3)	44.26 (10.54)	151 (98.1)	137 (89.0)	24.41 (3.03)	4.51 (0.66)
Canada	276	14 (5.1)	30 (10.9)	28 (10.1)	10 (3.6)	209 (75.7)	107 (38.8)	48.31 (12.04)	274 (99.3)	217 (78.6)	31.22 (2.77)	4.85 (0.71)
Chile	130	14 (10.8)	26 (20.0)	12 (9.2)		54 (41.5)	35 (26.9)	37.73 (7.95)	127 (97.7)	121 (93.1)	30.77 (1.08)	4.71 (0.61)
China	469	256 (54.6)	97 (20.7)	13 (2.8)	43 (9.2)	427 (91.0)	170 (36.2)	33.38 (6.39)	463 (98.7)	321 (68.4)	27.36 (1.26)	4.33 (0.50)
Colombia	84	26 (31.0)	20 (23.8)	6 (7.1)	1 (1.2)	62 (73.8)	24 (28.6)	39.82 (10.59)	83 (98.8)	78 (92.9)	33.94 (1.01)	4.63 (0.66)
Denmark	134	4 (3.0)	20 (14.9)	17 (12.7)	9 (6.7)	95 (70.9)	34 (25.4)	53.60 (12.29)	133 (99.3)	109 (81.3)	26.48 (4.26)	4.87 (0.59)
France	111	49 (44.1)	18 (16.2)	23 (20.7)		59 (53.2)	47 (42.3)	50.71 (8.54)	107 (96.4)	54 (48.6)	22.11 (1.09)	4.43 (0.88)
Germany	211	27 (12.8)	11 (5.2)	66 (31.3)	10 (4.7)	195 (92.4)	98 (46.4)	46.62 (9.75)	194 (91.9)	131 (62.1)	16.92 (0.58)	4.49 (0.74)
India	97	3 (3.1)	18 (18.6)	8 (8.2)	2 (2.1)	57 (58.8)	13 (13.4)	38.87 (9.72)	95 (97.9)	91 (93.8)	32.09 (3.72)	4.54 (0.74)
Italy	135	3 (2.2)	18 (13.3)	18 (13.3)	12 (8.9)	69 (51.1)	36 (26.7)	41.02 (11.20)	134 (99.3)	110 (81.5)	30.10 (2.50)	4.59 (0.66)
Japan	71	11 (15.5)	11 (15.5)	8 (11.3)	22 (31.0)	37 (52.1)	23 (32.4)	46.21 (14.85)	65 (91.5)	37 (52.1)	25.59 (1.20)	3.72 (0.74)
New Zealand	118	12 (10.2)	18 (15.3)	18 (15.3)		82 (69.5)	37 (31.4)	49.81 (12.08)	109 (92.4)	78 (66.1)	24.84 (5.25)	4.74 (0.62)
Pakistan	93	18 (19.4)	24 (25.8)	9 (9.7)	19 (20.4)	65 (69.9)	13 (14.0)	36.04 (8.99)	88 (94.6)	54 (58.1)	27.97 (2.78)	4.14 (0.73)
Poland	85	12 (14.1)	10 (11.8)	15 (17.6)	2 (2.4)	57 (67.1)	38 (44.7)	40.93 (9.57)	84 (98.8)	70 (82.4)	22.45 (5.30)	4.64 (0.72)
Spain	215	62 (28.8)	29 (13.5)	33 (15.3)	9 (4.2)	155 (72.1)	95 (44.2)	46.40 (11.20)	192 (89.3)	130 (60.5)	22.18 (2.38)	4.52 (0.71)
UK	374	10 (2.7)	35 (9.4)	19 (5.1)	16 (4.3)	274 (73.3)	99 (26.5)	48.47 (10.82)	352 (94.1)	272 (72.7)	25.82 (2.08)	4.88 (0.68)
USA	147	10 (6.8)	4 (2.7)	19 (12.9)		80 (54.4)	41 (27.9)	47.06 (11.94)	147 (100.0)	130 (88.4)	19.66 (0.76)	

For Review Only

Summary Robustness Checks

The robustness checks that we conducted are summarized in Table A2. In total, we conducted 18 robustness checks and in these tested 350 coefficients that reflect our hypotheses (across three dependent variables). Our results replicate with the same or stronger significance levels except for 5 of the 350 coefficients where significance levels for coefficients change from $p<.05$ to $p=.05$ or $p=.06$[1]. We replicate our findings in following robustness checks:

- taking into account shorter and longer time periods of stringency of lockdown and government support (January to July, Table A3, and January to September, A4),
- controlling only for GDP at the country level (Table A5) to account for multicollinearity among country-level controls,
- considering additional effects of the pandemic by expanding the set of country-level controls to include the number of Covid-19 cases per million people (Table A6) and number of Covid-19 deaths per million people (A7),
- controlling for whether the entrepreneur applied for government support or not (Table A8)
- using slightly different versions of our measure of adverse impact (Tables A9-A11):
    - the first alternative version of this measure (Table A9) combines the trading and threat question with equal weight after first z-standardizing them so that they are on the same scale and can be combined. The impact on trading was coded three-point scale 1=increased trading; 2=no change; 3=decreased trading before it was z-standardized. Cronbach Alpha for this index was .69,
    - the second alternative version of the index (Table A10) gives slightly greater emphasis to the question whether the existence of the business is threatened – it captures the adverse impact on the business from "-1" positive impact to "2" the most negative adverse impact. The value of "2" reflects 'yes' that the existence of the business is threatened by the pandemic, regardless of the impact on business trading. If the entrepreneur responded that the existence was not threatened, we coded as "1" if there was a decline in business trading, "0" if there was no change in business trading and "–1" if the treading increased.
    - third alternative version of the index (Table A11) is coded: "2" for decrease in trading and

[1] When we use the longer time period the significance of the indirect effect postulated in H1 (severity of lockdown-adverse impact on business-agility-WB) weakens to p=0.06 instead of p=0.04 for all three measures of WB. When we use limited set of control variables and when we treat life satisfaction as ordinal variable and run probit regression the interaction term between opportunity agility and planning agility postulated in H3c weakens to p=0.051.

For Review Only

threat to existence, “1” for decrease in trading and no threat to existence or no change in trading and threat to existence, “0” for no change in trading and no threat to existence or increase in trading and threat to existence, and “-1” for increase in trading and no threat to existence.

- testing the effect of adverse impact on agility and wellbeing with the inclusion of trait resilience (Table 7 in the paper) and the interaction term between adverse impact and trait resilience (A12),
- considering different sample sizes – due to missing data our sample for subjective vitality and distress as dependent variables is more limited. Hence, as a robustness check we limit the sample at all stages of analyses and replicate our results (Tables A13 and A14),
- replicating the findings with limited set of control variables (A15) – we apply country-level control variables only for the first stage of GSEM model (estimating the relationship of country-level lockdowns on the adverse impact on the business), we use firm-level control variables for the second stage (estimating the effect of adverse impact on the business on agility), we use individual-level control variables for the third stage (estimating the effect of agility on wellbeing), and we control for week of data collection at all stages of the GSEM model,
- testing the effect of agility on wellbeing with stringency of lockdown and adverse impact on the business removed from the model (Table A16),
- treating life satisfaction as ordinal variable to conduct the analysis as logit and probit regression (Tables A17 and A18),
- including the square of the week of data collection to account for a possible non-linear effect (Table A19),
- adjusting the week of data collection for the time since the introduction of lockdown in a country (Table A20),
- using pre-pandemic data from Polish and Spanish entrepreneurs to conduct a longitudinal analysis of the effect of pre-pandemic hedonic (life satisfaction) and eudaimonic (subjective vitality) on agility (Table A21) to address reverse causality.
- Table 22 presents the indirect effects for hypotheses H1 and H4 for all of the robustness checks. For each of the dependent variables we present the values of coefficients for indirect effects severity of lockdown-adverse impact on business-well-being (H1), severity of lockdown-adverse impact on business-agility-well-being and adverse impact on business-agility-well-being (H4).

For Review Only

Table A2. Overview Table Robustness Checks

Table No.	Description
A3	Stringency of lockdown replaced with the measure with the average from 7 months (January to July)
A4	Stringency of lockdown replaced with the measure with the average from 9 months (January to September)
A5	With only GDP as a control variable at the country level
A6	Number of Covid-19 cases per million people as a control variable
A7	Number of Covid-19 deaths per million people as a control variable
A8	Application for government support by the entrepreneur as a control variable
A9	Measure of adverse impact on business which combines the change in trading and threat to existence as the mean from z-standardized measures
A10	Measure of adverse impact on business which gives greater emphasis to the threat to the existence of the business
A11	Measure of adverse impact on business which gives the equal weight the change in trading and the threat to existence of the business
A12	The effect of adverse impact on business and trait resilience on agility and well-being including the interaction between adverse impact and trait resilience
A13	Limited sample size to account for missing data for vitality
A14	Limited sample size to account for missing data for distress
A15	Limited set of control variables
A16	The effect of agility on well-being with adverse impact and lockdowns removed from the model
A17	Life satisfaction as an ordinal variable – logit regression
A18	Life satisfaction as an ordinal variable – probit regression
A19	Squared value of Week included
A20	The value of Week variable adjusted for the country-level start of lockdown
A21	Longitudinal analysis of the effect of pre-pandemic hedonic (life satisfaction) and eudaimonic (subjective vitality) on agility
A22	The indirect effects for hypotheses H1 and H4 for all of the robustness checks

For Review Only

Table A3. Robustness test with stringency of lockdown replaced with the measure with the average from 7 months (January to July)

	Models 1, 2 and 3			Model 1	Model 2	Model 3
	Adverse impact	Opportunity agility	Planning agility	Life satisfaction	Subjective vitality	Distress
Column	1	2	3	4	5	6
Severity of lockdown	0.135*	-0.098	0.022	0.185	0.110*	-0.062
	(0.057)	(0.127)	(0.057)	(0.130)	(0.046)	(0.048)
Adverse impact on business		-0.393***	0.212***	-0.254***	-0.085***	0.100***
		(0.041)	(0.018)	(0.036)	(0.015)	(0.013)
Opportunity agility				0.417***	0.238***	-0.120***
				(0.074)	(0.031)	(0.026)
Planning agility				-0.163***	-0.038*	0.071***
				(0.043)	(0.018)	(0.015)
Planning agility* Opp. agility				0.146*	0.069*	-0.038
				(0.070)	(0.030)	(0.025)
Controls						
GDP ppp pc 2019	-0.135	0.336+	0.049	0.092	-0.086	-0.015
	(0.088)	(0.195)	(0.088)	(0.200)	(0.073)	(0.077)
Government support	0.017	-0.159	0.006	0.087	-0.045	0.028
	(0.080)	(0.177)	(0.079)	(0.181)	(0.063)	(0.066)
Firm age	0.038+	-0.166***	-0.025	-0.022	-0.029+	0.010
	(0.020)	(0.049)	(0.020)	(0.039)	(0.016)	(0.013)
Firm size (log employees)	-0.051**	0.316***	0.114***	0.120**	0.064***	-0.049***
	(0.020)	(0.047)	(0.020)	(0.039)	(0.016)	(0.014)
Industry: retail and gastronomy	0.118*	-0.388**	0.013	0.009	-0.007	0.083*
	(0.053)	(0.126)	(0.054)	(0.103)	(0.043)	(0.036)
Industry: manufacturing	0.004	-0.554***	0.046	0.165	0.064	0.047
	(0.054)	(0.126)	(0.054)	(0.104)	(0.043)	(0.036)
Industry: human oriented services	0.255***	-0.260+	0.015	0.126	-0.017	0.019
	(0.057)	(0.134)	(0.058)	(0.111)	(0.050)	(0.040)
Industry: other	0.015	-0.572**	-0.118	-0.188	0.006	0.149**
	(0.078)	(0.192)	(0.078)	(0.151)	(0.062)	(0.052)
Firm profit last year	-0.089*	-0.203*	-0.106**	0.186*	-0.011	-0.115***
	(0.040)	(0.093)	(0.040)	(0.078)	(0.033)	(0.028)
Entrepreneur gender (1=women)	0.043	0.137	0.001	-0.137+	-0.123***	0.129***
	(0.037)	(0.087)	(0.038)	(0.072)	(0.031)	(0.025)
Entrepreneur age	0.035+	-0.118*	-0.063**	0.094*	0.079***	-0.114***
	(0.021)	(0.049)	(0.021)	(0.041)	(0.017)	(0.014)
Secondary education	-0.071	-0.130	0.050	-0.106	-0.080	0.071
	(0.097)	(0.239)	(0.098)	(0.189)	(0.082)	(0.065)
University education	0.030	0.188+	0.062	0.176*	0.010	-0.016
	(0.042)	(0.102)	(0.042)	(0.081)	(0.035)	(0.028)
Week of data collection	-0.072*	0.091	-0.070*	0.100+	-0.006	0.005
	(0.029)	(0.066)	(0.029)	(0.057)	(0.023)	(0.020)
Constant	-0.016	-0.147	-0.046	6.362***	3.389***	2.729***
	(0.109)	(0.260)	(0.109)	(0.221)	(0.092)	(0.078)
N Individ./Countries	3,162 / 20	3,162 / 20	3,162 / 20	3,162 / 20	2,767 / 18	2,974 / 19
Overall R^2				0.071	0.107	0.091
Indiv./Country R^2				0.053/0.287	0.068/0.545	0.095/0.033
- 2 log likelihood				-16985.319	-13621.899	-13404.195

Standard errors in parentheses, *** p<0.001, ** p<0.01, * p<0.05, + p<0.1, see methods for variable definitions

For Review Only

Table A4. Robustness test with stringency of lockdown replaced with the measure with the average from 9 months (January to September)

	Models 1, 2 and 3<br>Adverse impact	 Opportunity agility	 Planning agility	Model 1<br>Life satisfaction	Model 2<br>Subjective vitality	Model 3<br>Distress
Column	1	2	3	4	5	6
Severity of	0.119*	-0.002	0.083	0.109	0.125**	-0.072
lockdown	(0.060)	(0.130)	(0.055)	(0.135)	(0.045)	(0.049)
Adverse impact on		-0.394***	0.211***	-0.253***	-0.085***	0.100***
business		(0.041)	(0.018)	(0.036)	(0.015)	(0.013)
Opportunity agility				0.416***	0.237***	-0.120***
				(0.074)	(0.031)	(0.026)
Planning agility				-0.164***	-0.039*	0.072***
				(0.043)	(0.018)	(0.015)
Planning agility*				0.145*	0.069*	-0.038
Opp. agility				(0.070)	(0.030)	(0.025)
Controls						
GDP ppp pc 2019	-0.147	0.398*	0.087	0.043	-0.070	-0.023
	(0.091)	(0.199)	(0.084)	(0.205)	(0.071)	(0.076)
Government	0.020	-0.177	-0.005	0.101	-0.052	0.030
support	(0.082)	(0.181)	(0.076)	(0.186)	(0.061)	(0.066)
Firm age	0.038+	-0.162**	-0.023	-0.023	-0.028+	0.010
	(0.020)	(0.049)	(0.020)	(0.039)	(0.016)	(0.013)
Firm size (log	-0.051*	0.314***	0.112***	0.121**	0.064***	-0.049***
employees)	(0.020)	(0.047)	(0.020)	(0.039)	(0.016)	(0.014)
Industry: retail and	0.120*	-0.393**	0.009	0.014	-0.005	0.083*
gastronomy	(0.053)	(0.126)	(0.054)	(0.103)	(0.043)	(0.036)
Industry:	0.005	-0.554***	0.046	0.166	0.065	0.047
manufacturing	(0.054)	(0.126)	(0.054)	(0.104)	(0.043)	(0.036)
Industry: human	0.255***	-0.259+	0.016	0.127	-0.016	0.019
oriented services	(0.057)	(0.134)	(0.058)	(0.111)	(0.050)	(0.040)
Industry: other	0.015	-0.568**	-0.117	-0.188	0.007	0.149**
	(0.078)	(0.192)	(0.078)	(0.151)	(0.062)	(0.052)
Firm profit last	-0.088*	-0.205*	-0.108**	0.186*	-0.011	-0.115***
year	(0.040)	(0.093)	(0.040)	(0.078)	(0.033)	(0.028)
Entrepreneur	0.043	0.136	0.001	-0.137+	-0.124***	0.129***
gender (1=women)	(0.037)	(0.087)	(0.038)	(0.072)	(0.031)	(0.025)
Entrepreneur age	0.035+	-0.118*	-0.062**	0.094*	0.079***	-0.114***
	(0.021)	(0.049)	(0.021)	(0.041)	(0.017)	(0.014)
Secondary	-0.069	-0.133	0.048	-0.104	-0.079	0.071
education	(0.097)	(0.239)	(0.098)	(0.189)	(0.082)	(0.065)
University	0.030	0.188+	0.062	0.175*	0.009	-0.015
education	(0.042)	(0.102)	(0.042)	(0.081)	(0.035)	(0.028)
Week of data	-0.072*	0.089	-0.070*	0.100+	-0.009	0.006
collection	(0.029)	(0.066)	(0.029)	(0.057)	(0.023)	(0.020)
Constant	-0.018	-0.144	-0.044	6.361***	3.392***	2.728***
	(0.109)	(0.261)	(0.109)	(0.223)	(0.092)	(0.078)
N Individ./Countries	3,162 / 20	3,162 / 20	3,162 / 20	3,162 / 20	2,767 / 18	2,974 / 19
Overall R^2				0.067	0.110	0.093
Indiv./Country R^2				0.053/0.233	0.068/0.588	0.095/0.061
- 2 log likelihood				-16985.807	-13620.995	-13403.801

Standard errors in parentheses, *** p<0.001, ** p<0.01, * p<0.05, + p<0.1, see methods for variable definitions

For Review Only

Table A5. Robustness check with only GDP as the control variable at the country level

	Models 1, 2 and 3			Model 1	Model 2	Model 3
	Adverse impact	Opportunity agility	Planning agility	Life satisfaction	Subjective vitality	Distress
Column	1	2	3	4	5	6
Severity of lockdown	0.136*	-0.060	0.058	0.170	0.121**	-0.066
	(0.059)	(0.134)	(0.057)	(0.135)	(0.046)	(0.049)
Adverse impact on business		-0.393***	0.211***	-0.254***	-0.085***	0.100***
		(0.041)	(0.018)	(0.036)	(0.015)	(0.013)
Opportunity agility				0.416***	0.238***	-0.120***
				(0.074)	(0.031)	(0.026)
Planning agility				-0.164***	-0.038*	0.072***
				(0.043)	(0.018)	(0.015)
Planning agility* Opp. agility				0.145*	0.069*	-0.038
				(0.070)	(0.030)	(0.025)
Controls						
GDP ppp pc 2019	-0.119*	0.231+	0.073	0.156	-0.110*	0.003
	(0.061)	(0.139)	(0.059)	(0.137)	(0.052)	(0.053)
Firm age	0.039+	-0.163***	-0.024	-0.022	-0.028+	0.010
	(0.020)	(0.049)	(0.020)	(0.039)	(0.016)	(0.013)
Firm size (log employees)	-0.051**	0.316***	0.113***	0.120**	0.064***	-0.049***
	(0.020)	(0.047)	(0.020)	(0.039)	(0.016)	(0.014)
Industry: retail and gastronomy	0.119*	-0.388**	0.011	0.011	-0.005	0.083*
	(0.053)	(0.126)	(0.054)	(0.103)	(0.043)	(0.036)
Industry: manufacturing	0.005	-0.551***	0.046	0.165	0.065	0.047
	(0.054)	(0.126)	(0.054)	(0.104)	(0.043)	(0.036)
Industry: human oriented services	0.255***	-0.259+	0.016	0.127	-0.016	0.019
	(0.057)	(0.134)	(0.058)	(0.111)	(0.050)	(0.040)
Industry: other	0.014	-0.556**	-0.117	-0.191	0.011	0.148**
	(0.077)	(0.192)	(0.078)	(0.150)	(0.062)	(0.051)
Firm profit last year	-0.089*	-0.204*	-0.107**	0.186*	-0.012	-0.115***
	(0.040)	(0.093)	(0.040)	(0.078)	(0.033)	(0.028)
Entrepreneur gender (1=women)	0.043	0.135	0.001	-0.137+	-0.124***	0.129***
	(0.037)	(0.087)	(0.038)	(0.072)	(0.031)	(0.025)
Entrepreneur age	0.035+	-0.121*	-0.062**	0.095*	0.078***	-0.113***
	(0.021)	(0.048)	(0.021)	(0.041)	(0.017)	(0.014)
Secondary education	-0.071	-0.126	0.048	-0.107	-0.078	0.070
	(0.097)	(0.239)	(0.098)	(0.189)	(0.082)	(0.065)
University education	0.030	0.186+	0.062	0.176*	0.009	-0.015
	(0.042)	(0.101)	(0.042)	(0.081)	(0.035)	(0.028)
Week of data collection	-0.073*	0.088	-0.070*	0.099+	-0.008	0.005
	(0.029)	(0.067)	(0.029)	(0.057)	(0.023)	(0.020)
Constant	-0.016	-0.154	-0.045	6.365***	3.390***	2.729***
	(0.109)	(0.262)	(0.109)	(0.222)	(0.092)	(0.078)
N Individ./Countries	3,162 / 20	3,162 / 20	3,162 / 20	3,162 / 20	2,767 / 18	2,974 / 19
Overall R^2				0.068	0.108	0.091
Indiv./Country R^2				0.053/0.255	0.068?0.562	0.095/0.035
- 2 log likelihood				-16986.135	-13621.946	-13404.574

Standard errors in parentheses, *** p<0.001, ** p<0.01, * p<0.05, + p<0.1, see methods for variable definitions

For Review Only

Table A6. Robustness check with the number of Covid-19 cases per million people as a control variable

	Models 1, 2 and 3			Model 1	Model 2	Model 3
	Adverse impact	Opportunity agility	Planning agility	Life satisfaction	Subjective vitality	Distress
Column	1	2	3	4	5	6
Severity of lockdown	0.154**	-0.091	0.041	0.168	0.126**	-0.070
	(0.052)	(0.111)	(0.051)	(0.135)	(0.045)	(0.049)
Adverse impact on business		-0.393***	0.212***	-0.254***	-0.084***	0.100***
		(0.041)	(0.018)	(0.036)	(0.015)	(0.013)
Opportunity agility				0.417***	0.237***	-0.120***
				(0.074)	(0.031)	(0.026)
Planning agility				-0.163***	-0.039*	0.071***
				(0.043)	(0.018)	(0.015)
Planning agility* Opp. agility				0.145*	0.069*	-0.038
				(0.070)	(0.030)	(0.025)
Controls						
GDP ppp pc 2019	-0.163*	0.441**	0.108	0.050	-0.054	-0.033
	(0.078)	(0.168)	(0.077)	(0.203)	(0.075)	(0.081)
Government support	0.062	-0.273+	-0.049	0.133	-0.062	0.042
	(0.071)	(0.153)	(0.070)	(0.185)	(0.064)	(0.068)
Number of cases per million people	-0.111*	0.258**	0.106*	-0.056	0.023	-0.002
	(0.044)	(0.095)	(0.044)	(0.117)	(0.044)	(0.048)
Firm age	0.039*	-0.165***	-0.025	-0.022	-0.029+	0.010
	(0.020)	(0.049)	(0.020)	(0.039)	(0.016)	(0.013)
Firm size (log employees)	-0.053**	0.324***	0.116***	0.120**	0.064***	-0.049***
	(0.020)	(0.047)	(0.020)	(0.039)	(0.016)	(0.014)
Industry: retail and gastronomy	0.122*	-0.402**	0.009	0.012	-0.006	0.083*
	(0.053)	(0.126)	(0.054)	(0.103)	(0.043)	(0.036)
Industry: manufacturing	0.007	-0.564***	0.045	0.166	0.065	0.047
	(0.054)	(0.126)	(0.054)	(0.104)	(0.043)	(0.036)
Industry: human oriented services	0.256***	-0.265*	0.014	0.127	-0.017	0.019
	(0.057)	(0.134)	(0.058)	(0.111)	(0.050)	(0.040)
Industry: other	0.016	-0.570**	-0.116	-0.188	0.007	0.150**
	(0.078)	(0.192)	(0.078)	(0.151)	(0.062)	(0.052)
Firm profit last year	-0.091*	-0.199*	-0.103*	0.185*	-0.010	-0.115***
	(0.040)	(0.093)	(0.040)	(0.078)	(0.033)	(0.028)
Entrepreneur gender (1=women)	0.044	0.135	0.001	-0.137+	-0.124***	0.129***
	(0.037)	(0.087)	(0.038)	(0.072)	(0.031)	(0.025)
Entrepreneur age	0.035+	-0.116*	-0.062**	0.094*	0.079***	-0.114***
	(0.021)	(0.049)	(0.021)	(0.041)	(0.017)	(0.014)
Secondary education	-0.070	-0.135	0.048	-0.105	-0.079	0.071
	(0.097)	(0.239)	(0.098)	(0.189)	(0.082)	(0.065)
University education	0.032	0.180+	0.058	0.177*	0.009	-0.015
	(0.042)	(0.101)	(0.042)	(0.081)	(0.035)	(0.028)
Week of data collection	-0.078**	0.110+	-0.064*	0.099+	-0.009	0.006
	(0.028)	(0.065)	(0.029)	(0.057)	(0.023)	(0.020)
Constant	-0.019	-0.134	-0.043	6.361***	3.395***	2.727***
	(0.106)	(0.254)	(0.107)	(0.222)	(0.092)	(0.078)
N Individ./Countries	3,162 / 20	3,162 / 20	3,162 / 20	3,162 / 20	2,767 / 18	2,974 / 19
Overall R^2				0.070	0.111	0.093
Indiv./Country R^2				0.053/0.278	0.068/0.594	0.095/0.061
- 2 log likelihood				-16977.151	-13612.787	-13395.693

Standard errors in parentheses, *** p<0.001, ** p<0.01, * p<0.05, + p<0.1, see methods for variable definitions

For Review Only

Table A7. Robustness check with the number of Covid-19 deaths per million people as a control variable

	Models 1, 2 and 3			Model 1	Model 2	Model 3
	Adverse impact	Opportunity agility	Planning agility	Life satisfaction	Subjective vitality	Distress
Column	1	2	3	4	5	6
Severity of	0.170**	-0.112	0.031	0.188	0.130**	-0.075
lockdown	(0.052)	(0.121)	(0.054)	(0.137)	(0.046)	(0.050)
Adverse impact on		-0.392***	0.212***	-0.255***	-0.085***	0.100***
business		(0.041)	(0.018)	(0.036)	(0.015)	(0.013)
Opportunity agility				0.418***	0.238***	-0.120***
				(0.074)	(0.031)	(0.026)
Planning agility				-0.163***	-0.038*	0.071***
				(0.043)	(0.018)	(0.015)
Planning agility*				0.145*	0.069*	-0.038
Opp. agility				(0.070)	(0.030)	(0.025)
Controls						
GDP ppp pc 2019	-0.130+	0.362*	0.077	0.067	-0.069	-0.030
	(0.076)	(0.176)	(0.078)	(0.197)	(0.071)	(0.076)
Government	0.067	-0.263	-0.047	0.155	-0.042	0.031
support	(0.071)	(0.165)	(0.073)	(0.184)	(0.063)	(0.067)
Number of deaths	-0.116*	0.223*	0.095*	-0.103	-0.017	0.020
per million people	(0.046)	(0.107)	(0.047)	(0.122)	(0.042)	(0.045)
Firm age	0.041*	-0.168***	-0.026	-0.021	-0.028+	0.010
	(0.020)	(0.049)	(0.020)	(0.039)	(0.016)	(0.013)
Firm size (log	-0.054**	0.323***	0.116***	0.119**	0.063***	-0.048***
employees)	(0.020)	(0.047)	(0.020)	(0.039)	(0.016)	(0.014)
Industry: retail and	0.123*	-0.397**	0.010	0.011	-0.006	0.083*
gastronomy	(0.053)	(0.126)	(0.054)	(0.103)	(0.043)	(0.036)
Industry:	0.008	-0.561***	0.045	0.166	0.065	0.047
manufacturing	(0.054)	(0.126)	(0.054)	(0.104)	(0.043)	(0.036)
Industry: human	0.256***	-0.262*	0.015	0.127	-0.016	0.019
oriented services	(0.057)	(0.134)	(0.058)	(0.111)	(0.050)	(0.040)
Industry: other	0.017	-0.571**	-0.117	-0.189	0.006	0.150**
	(0.078)	(0.192)	(0.078)	(0.151)	(0.062)	(0.052)
Firm profit last	-0.091*	-0.199*	-0.104*	0.184*	-0.011	-0.115***
year	(0.040)	(0.093)	(0.040)	(0.078)	(0.033)	(0.028)
Entrepreneur	0.044	0.135	0.001	-0.136+	-0.124***	0.129***
gender (1=women)	(0.037)	(0.087)	(0.038)	(0.072)	(0.031)	(0.025)
Entrepreneur age	0.034	-0.116*	-0.062**	0.094*	0.079***	-0.114***
	(0.021)	(0.049)	(0.021)	(0.041)	(0.017)	(0.014)
Secondary	-0.071	-0.130	0.049	-0.105	-0.080	0.071
education	(0.097)	(0.239)	(0.098)	(0.189)	(0.082)	(0.065)
University	0.030	0.186+	0.060	0.177*	0.010	-0.016
education	(0.042)	(0.101)	(0.042)	(0.081)	(0.035)	(0.028)
Week of data	-0.074**	0.096	-0.068*	0.100+	-0.009	0.005
collection	(0.028)	(0.065)	(0.029)	(0.057)	(0.023)	(0.020)
Constant	-0.015	-0.147	-0.046	6.362***	3.393***	2.728***
	(0.106)	(0.256)	(0.107)	(0.221)	(0.092)	(0.078)
N Individ./Countries	3,162 / 20	3,162 / 20	3,162 / 20	3,162 / 20	2,767 / 18	2,974 / 19
Overall R^2				0.072	0.110	0.094
Indiv./Country R^2				0.053/0.300	0.068/0.591	0.095/0.074
- 2 log likelihood				-16978.79	-13614.718	-13397.467

Standard errors in parentheses, *** p<0.001, ** p<0.01, * p<0.05, + p<0.1, see methods for variable definitions

For Review Only

Table A8. Robustness check application for government support by the entrepreneur as a control variable

	Models 1, 2 and 3			Model 1	Model 2	Model 3
	Adverse impact	Opportunity agility	Planning agility	Life satisfaction	Subjective vitality	Distress
Column	1	2	3	4	5	6
Severity of lockdown	0.135*	0.084	0.123	0.074	0.144*	-0.028
	(0.059)	(0.166)	(0.076)	(0.192)	(0.060)	(0.062)
Adverse impact on business		-0.403***	0.179***	-0.244***	-0.080***	0.090***
		(0.045)	(0.019)	(0.041)	(0.016)	(0.014)
Opportunity agility				0.412***	0.249***	-0.115***
				(0.086)	(0.035)	(0.029)
Planning agility				-0.156**	-0.040+	0.069***
				(0.054)	(0.022)	(0.018)
Planning agility* Opp. agility				0.235**	0.074*	-0.033
				(0.083)	(0.034)	(0.028)
Controls						
GDP ppp pc 2019	-0.131	0.425*	0.082	-0.062	-0.055	-0.016
	(0.089)	(0.215)	(0.097)	(0.246)	(0.077)	(0.080)
Government support	0.014	-0.212	-0.005	0.174	-0.055	0.020
	(0.079)	(0.178)	(0.081)	(0.206)	(0.064)	(0.067)
Firm age	0.039+	-0.163**	-0.025	-0.015	-0.026	0.003
	(0.020)	(0.051)	(0.021)	(0.042)	(0.017)	(0.014)
Firm size (log employees)	-0.051**	0.287***	0.091***	0.092*	0.059**	-0.039*
	(0.020)	(0.053)	(0.023)	(0.046)	(0.018)	(0.016)
Industry: retail and gastronomy	0.119*	-0.483**	-0.020	-0.092	0.007	0.094*
	(0.053)	(0.153)	(0.064)	(0.128)	(0.052)	(0.043)
Industry: manufacturing	0.005	-0.408**	0.070	0.241*	0.060	0.047
	(0.054)	(0.138)	(0.059)	(0.119)	(0.048)	(0.040)
Industry: human oriented services	0.255***	-0.157	0.038	0.049	-0.024	-0.001
	(0.057)	(0.150)	(0.066)	(0.131)	(0.053)	(0.045)
Industry: other	0.015	-0.487*	0.014	-0.050	0.027	0.150*
	(0.078)	(0.216)	(0.089)	(0.179)	(0.072)	(0.061)
Firm profit last year	-0.089*	-0.119	-0.110*	0.127	-0.037	-0.093**
	(0.040)	(0.101)	(0.043)	(0.087)	(0.035)	(0.030)
Applying for the gov. support		0.150	0.180***	-0.146	-0.055	0.051+
		(0.103)	(0.044)	(0.089)	(0.036)	(0.030)
Entrepreneur gender (1=women)	0.043	0.129	0.003	-0.254**	-0.160***	0.161***
	(0.037)	(0.102)	(0.044)	(0.088)	(0.036)	(0.030)
Entrepreneur age	0.035+	-0.091+	-0.063**	0.113*	0.083***	-0.121***
	(0.021)	(0.053)	(0.023)	(0.046)	(0.019)	(0.016)
Secondary education	-0.070	-0.252	0.039	-0.129	-0.102	0.063
	(0.097)	(0.260)	(0.109)	(0.219)	(0.089)	(0.075)
University education	0.030	0.159	0.044	0.168+	0.029	0.004
	(0.042)	(0.118)	(0.050)	(0.099)	(0.040)	(0.034)
Week of data collection	-0.073*	0.068	-0.087**	0.130*	-0.017	0.009
	(0.029)	(0.071)	(0.031)	(0.063)	(0.025)	(0.021)
Constant	-0.017	-0.091	-0.111	6.479***	3.464***	2.672***
	(0.109)	(0.288)	(0.124)	(0.266)	(0.101)	(0.089)
N Individ./Countries	3,162 / 18	2,293 / 18	2,293 / 18	2,293 / 18	2,289 / 18	2,285 / 18
Overall R^2				0.029	0.045	0.075
Indiv./Country R^2				0.024/0.095	-0.001/0.558	0.076/0.055
- 2 log likelihood				-13541.317	-11457.64	-11053.386

Standard errors in parentheses, *** p<0.001, ** p<0.01, * p<0.05, + p<0.1, see methods for variable definitions

For Review Only

Table A9. Robustness check with the measure of adverse impact on business which combines the change in trading and threat to existence as the mean from z-standardized measures

	Models 1, 2 and 3			Model 1	Model 2	Model 3
	Adverse impact	Opportunity agility	Planning agility	Life satisfaction	Subjective vitality	Distress
Column	1	2	3	4	5	6
Severity of lockdown	0.137*	-0.049	0.056	0.162	0.127**	-0.072
	(0.061)	(0.133)	(0.057)	(0.134)	(0.045)	(0.049)
Adverse impact on business		-0.368***	0.226***	-0.274***	-0.090***	0.109***
		(0.041)	(0.018)	(0.036)	(0.015)	(0.013)
Opportunity agility				0.413***	0.237***	-0.118***
				(0.074)	(0.031)	(0.026)
Planning agility				-0.157***	-0.037*	0.068***
				(0.043)	(0.018)	(0.015)
Planning agility* Opp. agility				0.148*	0.070*	-0.039
				(0.070)	(0.030)	(0.025)
Controls						
GDP ppp pc 2019	-0.122	0.367+	0.077	0.065	-0.067	-0.032
	(0.091)	(0.199)	(0.086)	(0.200)	(0.071)	(0.076)
Government support	0.007	-0.165	-0.004	0.108	-0.051	0.041
	(0.081)	(0.179)	(0.077)	(0.179)	(0.060)	(0.065)
Firm age	0.034+	-0.166***	-0.023	-0.022	-0.029+	0.010
	(0.020)	(0.049)	(0.020)	(0.039)	(0.016)	(0.013)
Firm size (log employees)	-0.053**	0.314***	0.114***	0.119**	0.063***	-0.048***
	(0.020)	(0.047)	(0.020)	(0.039)	(0.016)	(0.014)
Industry: retail and gastronomy	0.134*	-0.387**	0.006	0.018	-0.004	0.081*
	(0.053)	(0.126)	(0.053)	(0.103)	(0.043)	(0.036)
Industry: manufacturing	0.021	-0.545***	0.042	0.170	0.067	0.045
	(0.053)	(0.126)	(0.054)	(0.104)	(0.043)	(0.036)
Industry: human oriented services	0.262***	-0.264*	0.011	0.133	-0.015	0.016
	(0.057)	(0.134)	(0.058)	(0.111)	(0.050)	(0.039)
Industry: other	0.010	-0.570**	-0.117	-0.187	0.006	0.150**
	(0.078)	(0.192)	(0.078)	(0.150)	(0.062)	(0.051)
Firm profit last year	-0.116**	-0.210*	-0.100*	0.178*	-0.013	-0.112***
	(0.040)	(0.093)	(0.040)	(0.078)	(0.033)	(0.028)
Entrepreneur gender (1=women)	0.041	0.133	0.001	-0.137+	-0.124***	0.129***
	(0.037)	(0.087)	(0.038)	(0.072)	(0.031)	(0.025)
Entrepreneur age	0.028	-0.121*	-0.061**	0.093*	0.079***	-0.114***
	(0.021)	(0.048)	(0.021)	(0.041)	(0.017)	(0.014)
Secondary education	-0.059	-0.124	0.047	-0.103	-0.079	0.070
	(0.097)	(0.239)	(0.098)	(0.189)	(0.082)	(0.065)
University education	0.008	0.176+	0.066	0.169*	0.007	-0.013
	(0.042)	(0.101)	(0.042)	(0.081)	(0.034)	(0.028)
Week of data collection	-0.071*	0.092	-0.069*	0.099+	-0.009	0.006
	(0.029)	(0.066)	(0.029)	(0.057)	(0.023)	(0.020)
Constant	-0.002	-0.141	-0.048	6.366***	3.393***	2.725***
	(0.109)	(0.261)	(0.109)	(0.222)	(0.091)	(0.078)
N Individ./Countries	3,162 / 20	3,162 / 20	3,162 / 20	3,162 / 20	2,767 / 18	2,974 / 19
Overall R^2				0.072	0.111	0.096
Indiv./Country R^2				0.055/0.274	0.069/0.591	0.098/0.056
- 2 log likelihood				-16971.254	-13608.918	-13387.691

Standard errors in parentheses, *** p<0.001, ** p<0.01, * p<0.05, + p<0.1, see methods for variable definitions

For Review Only

Table A10. Robustness check with the measure of adverse impact on business which gives greater emphasis to the threat to the existence of the business

	Models 1, 2 and 3			Model 1	Model 2	Model 3
	Adverse impact	Opportunity agility	Planning agility	Life satisfaction	Subjective vitality	Distress
Column	1	2	3	4	5	6
Severity of lockdown	0.131* (0.059)	-0.056 (0.136)	0.059 (0.056)	0.159 (0.134)	0.126** (0.045)	-0.070 (0.049)
Adverse impact on business		-0.334*** (0.041)	0.214*** (0.018)	-0.260*** (0.036)	-0.087*** (0.015)	0.100*** (0.013)
Opportunity agility				0.425*** (0.074)	0.240*** (0.031)	-0.125*** (0.026)
Planning agility				-0.165*** (0.043)	-0.039* (0.018)	0.072*** (0.015)
Planning agility* Opp. agility				0.153* (0.070)	0.072* (0.030)	-0.040 (0.025)
Controls						
GDP ppp pc 2019	-0.113 (0.088)	0.372+ (0.203)	0.074 (0.084)	0.069 (0.199)	-0.066 (0.070)	-0.034 (0.076)
Government support	0.006 (0.079)	-0.164 (0.182)	-0.004 (0.075)	0.108 (0.178)	-0.051 (0.060)	0.041 (0.064)
Firm age	0.039+ (0.020)	-0.166*** (0.049)	-0.024 (0.020)	-0.021 (0.039)	-0.028+ (0.016)	0.010 (0.013)
Firm size (log employees)	-0.053** (0.020)	0.315*** (0.046)	0.113*** (0.020)	0.119** (0.039)	0.063*** (0.016)	-0.048*** (0.014)
Industry: retail and gastronomy	0.122* (0.053)	-0.394** (0.126)	0.009 (0.054)	0.015 (0.103)	-0.005 (0.043)	0.083* (0.036)
Industry: manufacturing	0.037 (0.054)	-0.536*** (0.125)	0.039 (0.054)	0.176+ (0.104)	0.069 (0.043)	0.043 (0.036)
Industry: human oriented services	0.242*** (0.057)	-0.279* (0.133)	0.018 (0.058)	0.126 (0.111)	-0.018 (0.050)	0.021 (0.039)
Industry: other	0.000 (0.078)	-0.570** (0.192)	-0.115 (0.078)	-0.189 (0.150)	0.004 (0.062)	0.150** (0.052)
Firm profit last year	-0.129** (0.040)	-0.209* (0.092)	-0.099* (0.040)	0.176* (0.078)	-0.013 (0.033)	-0.112*** (0.028)
Entrepreneur gender (1=women)	0.028 (0.037)	0.127 (0.087)	0.004 (0.038)	-0.141+ (0.072)	-0.125*** (0.031)	0.132*** (0.025)
Entrepreneur age	0.022 (0.021)	-0.123* (0.048)	-0.059** (0.021)	0.091* (0.041)	0.078*** (0.017)	-0.113*** (0.014)
Secondary education	-0.021 (0.098)	-0.105 (0.239)	0.038 (0.098)	-0.091 (0.189)	-0.075 (0.082)	0.066 (0.065)
University education	-0.010 (0.042)	0.168+ (0.101)	0.070+ (0.042)	0.165* (0.081)	0.006 (0.035)	-0.012 (0.028)
Week of data collection	-0.074* (0.029)	0.091 (0.067)	-0.069* (0.029)	0.098+ (0.057)	-0.009 (0.023)	0.006 (0.020)
Constant	-0.008 (0.109)	-0.145 (0.261)	-0.046 (0.109)	6.357*** (0.221)	3.391*** (0.091)	2.729*** (0.078)
N Individ./Countries	3,162 / 20	3,162 / 20	3,162 / 20	3,162 / 20	2,767 / 18	2,974 / 19
Overall R^2				0.071	0.111	0.094
Indiv./Country R^2				0.054/0.282	0.068/0.594	0.095/0.070
- 2 log likelihood				-17010.823	-13647.025	-13430.514

Standard errors in parentheses, *** p<0.001, ** p<0.01, * p<0.05, + p<0.1, see methods for variable definitions

For Review Only

Table A11. Robustness check with the measure of adverse impact on business which gives the equal weight the change in trading and the threat to existence of the business

	Models 1, 2 and 3			Model 1	Model 2	Model 3
	Adverse impact	Opportunity agility	Planning agility	Life satisfaction	Subjective vitality	Distress
Column	1	2	3	4	5	6
Severity of lockdown	0.134*	-0.048	0.058	0.161	0.127**	-0.071
	(0.060)	(0.133)	(0.057)	(0.134)	(0.045)	(0.049)
Adverse impact on business		-0.383***	0.217***	-0.266***	-0.089***	0.103***
		(0.041)	(0.018)	(0.036)	(0.015)	(0.013)
Opportunity agility				0.412***	0.236***	-0.119***
				(0.074)	(0.031)	(0.026)
Planning agility				-0.161***	-0.037*	0.071***
				(0.043)	(0.018)	(0.015)
Planning agility* Opp. agility				0.149*	0.070*	-0.039
				(0.070)	(0.030)	(0.025)
Controls						
GDP ppp pc 2019	-0.127	0.365+	0.077	0.065	-0.068	-0.032
	(0.090)	(0.198)	(0.086)	(0.200)	(0.071)	(0.076)
Government support	0.011	-0.164	-0.005	0.109	-0.051	0.041
	(0.080)	(0.178)	(0.076)	(0.179)	(0.060)	(0.064)
Firm age	0.040*	-0.163***	-0.024	-0.021	-0.028+	0.010
	(0.020)	(0.049)	(0.020)	(0.039)	(0.016)	(0.013)
Firm size (log employees)	-0.054**	0.313***	0.114***	0.119**	0.063***	-0.048***
	(0.020)	(0.047)	(0.020)	(0.039)	(0.016)	(0.014)
Industry: retail and gastronomy	0.121*	-0.392**	0.010	0.014	-0.005	0.083*
	(0.053)	(0.126)	(0.054)	(0.103)	(0.043)	(0.036)
Industry: manufacturing	0.016	-0.548***	0.043	0.169	0.066	0.046
	(0.054)	(0.126)	(0.054)	(0.104)	(0.043)	(0.036)
Industry: human oriented services	0.255***	-0.263*	0.014	0.129	-0.016	0.019
	(0.057)	(0.134)	(0.058)	(0.111)	(0.050)	(0.039)
Industry: other	0.006	-0.574**	-0.116	-0.188	0.005	0.150**
	(0.078)	(0.192)	(0.078)	(0.150)	(0.062)	(0.051)
Firm profit last year	-0.108**	-0.209*	-0.103*	0.180*	-0.013	-0.113***
	(0.040)	(0.093)	(0.040)	(0.078)	(0.033)	(0.028)
Entrepreneur gender (1=women)	0.038	0.133	0.002	-0.138+	-0.124***	0.130***
	(0.037)	(0.087)	(0.038)	(0.072)	(0.031)	(0.025)
Entrepreneur age	0.030	-0.120*	-0.061**	0.093*	0.079***	-0.113***
	(0.021)	(0.048)	(0.021)	(0.041)	(0.017)	(0.014)
Secondary education	-0.052	-0.122	0.045	-0.100	-0.078	0.069
	(0.097)	(0.239)	(0.098)	(0.189)	(0.082)	(0.065)
University education	0.015	0.181+	0.065	0.171*	0.008	-0.014
	(0.042)	(0.101)	(0.042)	(0.081)	(0.034)	(0.028)
Week of data collection	-0.075**	0.090	-0.069*	0.098+	-0.009	0.006
	(0.029)	(0.066)	(0.029)	(0.057)	(0.023)	(0.020)
Constant	-0.012	-0.144	-0.045	6.364***	3.393***	2.727***
	(0.109)	(0.261)	(0.109)	(0.222)	(0.092)	(0.078)
N Individ./Countries	3,162 / 20	3,162 / 20	3,162 / 20	3,162 / 20	2,767 / 18	2,974 / 19
Overall R^2				0.071	0.111	0.094
Indiv./Country R^2				0.055/0.274	0.069/0.589	0.096/0.061
- 2 log likelihood				-16981.798	-13618.479	-13400.85

Standard errors in parentheses, *** p<0.001, ** p<0.01, * p<0.05, + p<0.1, see methods for variable definitions

For Review Only

Table A12. Robustness check with the effect of adverse impact on business and trait resilience on agility including the interaction between adverse impact and trait resilience

	Models 1-5 Adverse impact	Model 1 Opportunity agility	Model 2 Planning agility	Model 3 Life satisfaction	Model 4 Subjective vitality	Model 5 Distress
Column	1	2	3			
Severity of lockdown	0.135* (0.059)	-0.107 (0.140)	0.174 (0.187)	0.003 (0.148)	0.060 (0.042)	-0.006 (0.049)
Adverse impact on business		-0.392*** (0.044)	0.676*** (0.061)	-0.274*** (0.036)	-0.085*** (0.014)	0.099*** (0.012)
Trait resilience		0.189*** (0.046)	0.112+ (0.062)	0.538*** (0.036)	0.316*** (0.014)	-0.277*** (0.012)
Adverse impact* Trait resilience		0.052 (0.043)	-0.005 (0.059)	0.007 (0.034)	0.012 (0.013)	-0.000 (0.011)
Controls						
GDP ppp pc 2019	-0.131 (0.089)	0.286 (0.218)	0.220 (0.289)	-0.090 (0.228)	-0.109+ (0.065)	0.014 (0.076)
Government support	0.014 (0.079)	-0.228 (0.185)	-0.025 (0.245)	0.064 (0.194)	-0.108+ (0.055)	0.083 (0.064)
Firm age	0.039+ (0.020)	-0.149** (0.050)	-0.062 (0.066)	0.015 (0.038)	-0.011 (0.015)	-0.008 (0.012)
Firm size (log employees)	-0.051** (0.020)	0.310*** (0.048)	0.346*** (0.066)	0.088* (0.038)	0.054*** (0.015)	-0.032** (0.012)
Industry: retail and gastronomy	0.119* (0.053)	-0.358** (0.130)	0.064 (0.175)	0.025 (0.102)	0.000 (0.040)	0.070* (0.033)
Industry: manufacturing	0.005 (0.054)	-0.517*** (0.129)	0.229 (0.176)	0.125 (0.103)	0.055 (0.040)	0.057+ (0.033)
Industry: human oriented services	0.255*** (0.057)	-0.175 (0.141)	0.091 (0.194)	0.026 (0.113)	-0.028 (0.047)	0.025 (0.037)
Industry: other	0.015 (0.078)	-0.569** (0.196)	-0.314 (0.253)	-0.218 (0.147)	0.008 (0.058)	0.142** (0.048)
Firm profit last year	-0.089* (0.040)	-0.171+ (0.098)	-0.369** (0.135)	0.104 (0.079)	-0.040 (0.031)	-0.096*** (0.026)
Entrepreneur gender (1=women)	0.043 (0.037)	0.122 (0.091)	-0.019 (0.124)	-0.151* (0.073)	-0.117*** (0.029)	0.130*** (0.024)
Entrepreneur age	0.035+ (0.021)	-0.118* (0.052)	-0.214** (0.071)	0.031 (0.042)	0.032* (0.016)	-0.078*** (0.013)
Secondary education	-0.070 (0.097)	-0.076 (0.247)	0.162 (0.319)	-0.138 (0.186)	-0.077 (0.077)	0.051 (0.060)
University education	0.030 (0.042)	0.218* (0.105)	0.221 (0.139)	0.223** (0.081)	0.018 (0.032)	-0.019 (0.026)
Week of data collection	-0.073* (0.029)	0.081 (0.072)	-0.232* (0.097)	0.130* (0.058)	-0.006 (0.022)	-0.001 (0.019)
Constant	-0.017 (0.109)	-0.270 (0.271)	4.155*** (0.355)	6.607*** (0.223)	3.513*** (0.085)	2.674*** (0.073)
N Individ./Countries	3,162 / 20	2,935 / 19	2,935 / 19	2,935 / 19	2,723 / 18	2,931 / 19
Overall R^2				0.113	0.225	0.219
Indiv./Country R^2				0.110/0.149	0.186/0.660	0.230/0.051
- 2 log likelihood				-10186.584	-7138.5973	-6878.4682

Standard errors in parentheses, *** p<0.001, ** p<0.01, * p<0.05, + p<0.1, see methods for variable definitions

For Review Only

Table A13. Robustness check with limited sample to account for missing data for vitality

	Adverse impact	Opportunity agility	Planning agility	Subjective vitality
Column	1	2	3	5
Severity of lockdown	0.142*	-0.063	0.063	0.126**
	(0.057)	(0.136)	(0.061)	(0.046)
Adverse impact on business		-0.375***	0.201***	-0.085***
		(0.043)	(0.019)	(0.015)
Opportunity agility				0.237***
				(0.031)
Planning agility				-0.038*
				(0.018)
Planning agility* Opp. agility				0.069*
				(0.030)
Controls				
GDP ppp pc 2019	-0.125	0.379+	0.082	-0.068
	(0.088)	(0.213)	(0.094)	(0.071)
Government support	0.009	-0.169	0.007	-0.050
	(0.075)	(0.180)	(0.080)	(0.060)
Firm age	0.040+	-0.165***	-0.027	-0.029+
	(0.021)	(0.050)	(0.021)	(0.016)
Firm size (log employees)	-0.033	0.313***	0.115***	0.064***
	(0.021)	(0.049)	(0.021)	(0.016)
Industry: retail and gastronomy	0.121*	-0.321*	0.017	-0.006
	(0.057)	(0.132)	(0.056)	(0.043)
Industry: manufacturing	0.023	-0.496***	0.076	0.065
	(0.056)	(0.129)	(0.055)	(0.043)
Industry: human oriented services	0.268***	-0.211	0.027	-0.017
	(0.065)	(0.147)	(0.065)	(0.050)
Industry: other	0.023	-0.516**	-0.088	0.006
	(0.081)	(0.197)	(0.080)	(0.062)
Firm profit last year	-0.108*	-0.121	-0.111**	-0.011
	(0.043)	(0.098)	(0.042)	(0.033)
Entrepreneur gender (1=women)	0.044	0.122	0.017	-0.124***
	(0.040)	(0.093)	(0.040)	(0.031)
Entrepreneur age	0.026	-0.086+	-0.056*	0.079***
	(0.023)	(0.052)	(0.022)	(0.017)
Secondary education	-0.027	-0.225	0.018	-0.080
	(0.107)	(0.252)	(0.106)	(0.082)
University education	0.009	0.250*	0.066	0.009
	(0.045)	(0.108)	(0.045)	(0.035)
Week of data collection	-0.058+	0.051	-0.082**	-0.009
	(0.030)	(0.071)	(0.031)	(0.023)
Constant	-0.052	-0.137	-0.014	3.392***
	(0.117)	(0.275)	(0.117)	(0.092)
N Individ./Countries	2,767 / 18	2,767 / 18	2,767 / 18	2,767 / 18
Overall R^2				0.110
Indiv./Country R^2				0.068/0.586
- 2 log likelihood				-12321.331

Standard errors in parentheses, *** p<0.001, ** p<0.01, * p<0.05, + p<0.1, see methods for variable definitions

For Review Only

Table A14. Robustness check with limited sample to account for missing data for distress

	Adverse impact	Opportunity agility	Planning agility	Distress
Column	1	2	3	6
Severity of lockdown	0.141*	-0.069	0.059	-0.070
	(0.063)	(0.143)	(0.061)	(0.049)
Adverse impact on business		-0.382***	0.209***	0.100***
		(0.043)	(0.019)	(0.013)
Opportunity agility				-0.120***
				(0.026)
Planning agility				0.071***
				(0.015)
Planning agility* Opp. agility				-0.038
				(0.025)
Controls				
GDP ppp pc 2019	-0.113	0.342	0.073	-0.032
	(0.097)	(0.222)	(0.094)	(0.076)
Government support	0.022	-0.211	-0.002	0.041
	(0.082)	(0.188)	(0.080)	(0.064)
Firm age	0.041*	-0.173***	-0.023	0.010
	(0.020)	(0.050)	(0.020)	(0.013)
Firm size (log employees)	-0.050*	0.322***	0.112***	-0.049***
	(0.020)	(0.048)	(0.020)	(0.014)
Industry: retail and gastronomy	0.121*	-0.335**	0.019	0.083*
	(0.053)	(0.129)	(0.054)	(0.036)
Industry: manufacturing	0.011	-0.504***	0.069	0.047
	(0.054)	(0.128)	(0.055)	(0.036)
Industry: human oriented services	0.262***	-0.195	0.036	0.019
	(0.059)	(0.140)	(0.060)	(0.040)
Industry: other	0.013	-0.539**	-0.106	0.150**
	(0.077)	(0.193)	(0.079)	(0.052)
Firm profit last year	-0.101*	-0.130	-0.110**	-0.115***
	(0.041)	(0.097)	(0.042)	(0.028)
Entrepreneur gender (1=women)	0.046	0.128	0.001	0.129***
	(0.038)	(0.090)	(0.039)	(0.025)
Entrepreneur age	0.021	-0.087+	-0.065**	-0.114***
	(0.022)	(0.051)	(0.022)	(0.014)
Secondary education	-0.047	-0.145	0.051	0.071
	(0.097)	(0.242)	(0.099)	(0.065)
University education	0.022	0.218*	0.070	-0.015
	(0.042)	(0.104)	(0.043)	(0.028)
Week of data collection	-0.067*	0.083	-0.070*	0.006
	(0.030)	(0.071)	(0.030)	(0.020)
Constant	-0.017	-0.245	-0.063	2.728***
	(0.110)	(0.267)	(0.111)	(0.078)
N Individ./Countries	2,974 / 19	2,974 / 19	2,974 / 19	2,974 / 19
Overall R^2				0.093
Indiv./Country R^2				0.095/0.061
- 2 log likelihood				-12725.93

Standard errors in parentheses, *** p<0.001, ** p<0.01, * p<0.05, + p<0.1, see methods for variable definitions

For Review Only

Table A15. Robustness check with limited set of control variables

	Models 1, 2 and 3			Model 1	Model 2	Model 3
	Adverse impact	Opportunity agility	Planning agility	Life satisfaction	Subjective vitality	Distress
Column	1	2	3	4	5	6
Severity of	0.134*	-0.172	0.023	0.135	0.195***	-0.080+
lockdown	(0.058)	(0.124)	(0.051)	(0.122)	(0.045)	(0.042)
Adverse impact on		-0.393***	0.211***	-0.262***	-0.087***	0.104***
business		(0.041)	(0.018)	(0.036)	(0.015)	(0.013)
Opportunity agility				0.430***	0.249***	-0.134***
				(0.074)	(0.031)	(0.026)
Planning agility				-0.150***	-0.032+	0.069***
				(0.043)	(0.018)	(0.015)
Planning agility*				0.137+	0.067*	-0.036
Opp. agility				(0.070)	(0.030)	(0.025)
Controls						
GDP ppp pc 2019	-0.128					
	(0.086)					
Government	0.026					
support	(0.077)					
Firm age	0.050**	-0.165***	-0.024			
	(0.019)	(0.049)	(0.020)			
Firm size (log	-0.054**	0.312***	0.112***			
employees)	(0.019)	(0.047)	(0.020)			
Industry: retail and	0.117*	-0.393**	0.008			
gastronomy	(0.053)	(0.126)	(0.054)			
Industry:	0.000	-0.561***	0.042			
manufacturing	(0.054)	(0.126)	(0.054)			
Industry: human	0.255***	-0.261+	0.015			
oriented services	(0.057)	(0.134)	(0.058)			
Industry: other	0.013	-0.571**	-0.122			
	(0.077)	(0.192)	(0.078)			
Firm profit last	-0.091*	-0.202*	-0.108**			
year	(0.040)	(0.093)	(0.040)			
Entrepreneur		0.135	0.001	-0.165*	-0.144***	0.145***
gender (1=women)		(0.087)	(0.038)	(0.072)	(0.030)	(0.025)
Entrepreneur age		-0.113*	-0.060**	0.097*	0.070***	-0.115***
		(0.048)	(0.021)	(0.038)	(0.016)	(0.014)
Secondary		-0.120	0.050	-0.090	-0.070	0.057
education		(0.239)	(0.098)	(0.189)	(0.082)	(0.065)
University		0.184+	0.061	0.206*	0.022	-0.037
education		(0.101)	(0.042)	(0.080)	(0.034)	(0.028)
Week of data	-0.076**	0.063	-0.078**	0.079	-0.004	0.008
collection	(0.028)	(0.065)	(0.028)	(0.057)	(0.024)	(0.020)
Constant	-0.047	-0.149	-0.042	6.485***	3.385***	2.718***
	(0.058)	(0.265)	(0.110)	(0.213)	(0.089)	(0.074)
N Individ./Countries	3,162 / 20	3,162 / 20	3,162 / 20	3,162 / 20	2,767 / 18	2,974 / 19
Overall R^2				0.053	0.086	0.075
Indiv./Country R^2				0.046/0.138	0.060/0.372	0.081/-0.004
- 2 log likelihood				-17003.871	-13639.814	-13432.435

Standard errors in parentheses, *** p<0.001, ** p<0.01, * p<0.05, + p<0.1, see methods for variable definitions

For Review Only

Table A16. Robustness check with the effect of agility on well-being with adverse impact and lockdowns removed from the model

	Model 1 Life satisfaction	Model 2 Subjective vitality	Model 3 Distress
Column	4	5	6
Opportunity agility	0.529***	0.274***	-0.163***
	(0.073)	(0.031)	(0.026)
Planning agility	-0.220***	-0.057**	0.093***
	(0.042)	(0.018)	(0.015)
Planning agility* Opp. agility	0.141*	0.067*	-0.035
	(0.070)	(0.030)	(0.025)
Controls			
GDP ppp pc 2019	0.005	-0.149*	0.003
	(0.187)	(0.073)	(0.068)
Government support	0.134	-0.026	0.029
	(0.185)	(0.069)	(0.065)
Firm age	-0.031	-0.033*	0.014
	(0.039)	(0.016)	(0.014)
Firm size (log employees)	0.134***	0.068***	-0.054***
	(0.039)	(0.016)	(0.014)
Industry: retail and gastronomy	-0.001	-0.007	0.089*
	(0.104)	(0.043)	(0.036)
Industry: manufacturing	0.182+	0.068	0.041
	(0.105)	(0.043)	(0.036)
Industry: human oriented services	0.075	-0.035	0.040
	(0.112)	(0.050)	(0.040)
Industry: other	-0.184	0.007	0.149**
	(0.152)	(0.062)	(0.052)
Firm profit last year	0.206**	-0.002	-0.124***
	(0.079)	(0.033)	(0.028)
Entrepreneur gender (1=women)	-0.150*	-0.127***	0.134***
	(0.073)	(0.031)	(0.026)
Entrepreneur age	0.084*	0.076***	-0.111***
	(0.041)	(0.017)	(0.015)
Secondary education	-0.080	-0.073	0.063
	(0.190)	(0.082)	(0.066)
University education	0.168*	0.007	-0.013
	(0.082)	(0.035)	(0.029)
Week of data collection	0.111+	-0.002	0.001
	(0.058)	(0.024)	(0.020)
Constant	6.309***	3.370***	2.750***
	(0.225)	(0.095)	(0.079)
N Individ./Countries	3,162 / 20	2,767 / 18	2,974 / 19
Overall R^2	0.051	0.085	0.072
Indiv./Country R^2	0.038/0.208	0.057/0.398	0.076/0.016
- 2 log likelihood	-6443.1899	-3072.3835	-2867.3964

Standard errors in parentheses, *** p<0.001, ** p<0.01, * p<0.05, + p<0.1, see methods for variable definitions

For Review Only

For Review Only

Table A17. Robustness check with life satisfaction as ordinal variable – logit regression

	Adverse impact	Opportunity agility	Planning agility	Life satisfaction
Column	1	2	3	6
Severity of lockdown	0.135*	-0.047	0.059	0.152
	(0.059)	(0.132)	(0.057)	(0.133)
Adverse impact on business		-0.394***	0.211***	-0.280***
		(0.041)	(0.018)	(0.036)
Opportunity agility				0.405***
				(0.072)
Planning agility				-0.147***
				(0.041)
Planning agility* Opp. agility				0.157*
				(0.067)
Controls				
GDP ppp pc 2019	-0.131	0.363+	0.077	0.059
	(0.089)	(0.197)	(0.086)	(0.199)
Government support	0.014	-0.164	-0.005	0.098
	(0.079)	(0.177)	(0.077)	(0.178)
Firm age	0.039+	-0.164***	-0.024	-0.020
	(0.020)	(0.049)	(0.020)	(0.039)
Firm size (log employees)	-0.051**	0.315***	0.113***	0.132***
	(0.020)	(0.047)	(0.020)	(0.038)
Industry: retail and gastronomy	0.119*	-0.392**	0.011	0.081
	(0.053)	(0.126)	(0.054)	(0.100)
Industry: manufacturing	0.005	-0.554***	0.046	0.161
	(0.054)	(0.126)	(0.054)	(0.102)
Industry: human oriented services	0.255***	-0.260+	0.016	0.159
	(0.057)	(0.134)	(0.058)	(0.107)
Industry: other	0.015	-0.570**	-0.118	-0.186
	(0.078)	(0.192)	(0.078)	(0.144)
Firm profit last year	-0.089*	-0.203*	-0.107**	0.172*
	(0.040)	(0.093)	(0.040)	(0.076)
Entrepreneur gender (1=women)	0.043	0.136	0.001	-0.125+
	(0.037)	(0.087)	(0.038)	(0.070)
Entrepreneur age	0.035+	-0.117*	-0.062**	0.099*
	(0.021)	(0.049)	(0.021)	(0.039)
Secondary education	-0.070	-0.132	0.048	-0.088
	(0.097)	(0.239)	(0.098)	(0.183)
University education	0.030	0.188+	0.062	0.194*
	(0.042)	(0.102)	(0.042)	(0.078)
Week of data collection	-0.073*	0.090	-0.070*	0.073
	(0.029)	(0.066)	(0.029)	(0.056)
Constant	-0.017	-0.146	-0.045	
	(0.109)	(0.261)	(0.109)	
N Individ./Countries	3,162 / 20	3,162 / 20	3,162 / 20	3,162 / 20
Overall R^2				0.0020
Country R^2				0.254
- 2 log likelihood				-16695.3

Standard errors in parentheses, *** p<0.001, ** p<0.01, * p<0.05, + p<0.1, see methods for variable definitions

Table A18. Robustness check with life satisfaction as ordinal variable – probit regression

	Adverse impact	Opportunity agility	Planning agility	Life satisfaction
Column	1	2	3	6
Severity of lockdown	0.135*	-0.047	0.059	0.073
	(0.059)	(0.132)	(0.057)	(0.075)
Adverse impact on business		-0.394***	0.211***	-0.152***
		(0.041)	(0.018)	(0.020)
Opportunity agility				0.226***
				(0.041)
Planning agility				-0.080***
				(0.024)
Planning agility* Opp. agility				0.076+
				(0.039)
Controls				
GDP ppp pc 2019	-0.131	0.363+	0.077	0.026
	(0.089)	(0.197)	(0.086)	(0.112)
Government support	0.014	-0.164	-0.005	0.044
	(0.079)	(0.177)	(0.077)	(0.100)
Firm age	0.039+	-0.164***	-0.024	-0.007
	(0.020)	(0.049)	(0.020)	(0.022)
Firm size (log employees)	-0.051**	0.315***	0.113***	0.068**
	(0.020)	(0.047)	(0.020)	(0.022)
Industry: retail and gastronomy	0.119*	-0.392**	0.011	0.029
	(0.053)	(0.126)	(0.054)	(0.057)
Industry: manufacturing	0.005	-0.554***	0.046	0.112+
	(0.054)	(0.126)	(0.054)	(0.058)
Industry: human oriented services	0.255***	-0.260+	0.016	0.083
	(0.057)	(0.134)	(0.058)	(0.062)
Industry: other	0.015	-0.570**	-0.118	-0.103
	(0.078)	(0.192)	(0.078)	(0.084)
Firm profit last year	-0.089*	-0.203*	-0.107**	0.102*
	(0.040)	(0.093)	(0.040)	(0.044)
Entrepreneur gender (1=women)	0.043	0.136	0.001	-0.076+
	(0.037)	(0.087)	(0.038)	(0.040)
Entrepreneur age	0.035+	-0.117*	-0.062**	0.049*
	(0.021)	(0.049)	(0.021)	(0.023)
Secondary education	-0.070	-0.132	0.048	-0.061
	(0.097)	(0.239)	(0.098)	(0.105)
University education	0.030	0.188+	0.062	0.106*
	(0.042)	(0.102)	(0.042)	(0.045)
Week of data collection	-0.073*	0.090	-0.070*	0.042
	(0.029)	(0.066)	(0.029)	(0.032)
Constant	-0.017	-0.146	-0.045	
	(0.109)	(0.261)	(0.109)	
N Individ./Countries	3,162 / 20	3,162 / 20	3,162 / 20	3,162 / 20
Overall R^2				0.005
Country R^2				0.200
- 2 log likelihood				-16708.559

Standard errors in parentheses, *** p<0.001, ** p<0.01, * p<0.05, + p<0.1, see methods for variable definitions

For Review Only

Table A19. Robustness test with squared value of Week included (testing for nonlinear effects)

	Models 1, 2 and 3			Model 1	Model 2	Model 3
	Adverse impact	Opportunity agility	Planning agility	Life satisfaction	Subjective vitality	Distress
Column	1	2	3	4	5	6
Severity of lockdown	0.138*	-0.049	0.059	0.163	0.131**	-0.070
	(0.061)	(0.131)	(0.058)	(0.130)	(0.044)	(0.049)
Adverse impact on business		-0.393***	0.211***	-0.256***	-0.085***	0.100***
		(0.041)	(0.018)	(0.036)	(0.015)	(0.013)
Opportunity agility				0.417***	0.237***	-0.120***
				(0.074)	(0.031)	(0.026)
Planning agility				-0.164***	-0.039*	0.071***
				(0.043)	(0.018)	(0.015)
Planning agility* Opp. agility				0.146*	0.069*	-0.038
				(0.070)	(0.030)	(0.025)
Controls						
GDP ppp pc 2019	-0.129	0.363+	0.077	0.068	-0.058	-0.032
	(0.090)	(0.196)	(0.086)	(0.194)	(0.069)	(0.076)
Government support	-0.000	-0.158	-0.006	0.093	-0.056	0.041
	(0.081)	(0.176)	(0.077)	(0.173)	(0.059)	(0.065)
Firm age	0.040*	-0.165***	-0.024	-0.021	-0.028+	0.010
	(0.020)	(0.049)	(0.020)	(0.039)	(0.016)	(0.013)
Firm size (log employees)	-0.052**	0.315***	0.113***	0.120**	0.063***	-0.049***
	(0.020)	(0.047)	(0.020)	(0.039)	(0.016)	(0.014)
Industry: retail and gastronomy	0.121*	-0.394**	0.011	0.015	-0.005	0.083*
	(0.053)	(0.126)	(0.054)	(0.103)	(0.043)	(0.036)
Industry: manufacturing	0.009	-0.557***	0.046	0.171	0.067	0.047
	(0.054)	(0.126)	(0.054)	(0.104)	(0.043)	(0.036)
Industry: human oriented services	0.255***	-0.260+	0.016	0.127	-0.016	0.019
	(0.057)	(0.134)	(0.058)	(0.111)	(0.050)	(0.040)
Industry: other	0.025	-0.576**	-0.117	-0.174	0.011	0.150**
	(0.078)	(0.193)	(0.079)	(0.151)	(0.062)	(0.052)
Firm profit last year	-0.095*	-0.200*	-0.108**	0.180*	-0.013	-0.115***
	(0.040)	(0.093)	(0.040)	(0.078)	(0.033)	(0.028)
Entrepreneur gender (1=women)	0.041	0.138	0.001	-0.140+	-0.126***	0.129***
	(0.037)	(0.087)	(0.038)	(0.072)	(0.031)	(0.025)
Entrepreneur age	0.034	-0.117*	-0.062**	0.092*	0.079***	-0.114***
	(0.021)	(0.049)	(0.021)	(0.041)	(0.017)	(0.014)
Secondary education	-0.065	-0.135	0.049	-0.098	-0.077	0.071
	(0.097)	(0.239)	(0.098)	(0.189)	(0.082)	(0.065)
University education	0.032	0.188+	0.062	0.178*	0.010	-0.015
	(0.042)	(0.102)	(0.042)	(0.081)	(0.035)	(0.028)
Week of data collection	-0.085**	0.097	-0.071*	0.083	-0.019	0.006
	(0.029)	(0.067)	(0.030)	(0.057)	(0.024)	(0.021)
Squared Week of data collection	0.059*	-0.027	0.003	0.069	0.026	-0.001
	(0.023)	(0.053)	(0.023)	(0.046)	(0.020)	(0.016)
Constant	-0.086	-0.113	-0.049	6.279***	3.369***	2.728***
	(0.112)	(0.268)	(0.113)	(0.227)	(0.093)	(0.080)
N Individ./Countries	3,162 / 20	3,162 / 20	3,162 / 20	3,162 / 20	2,767 / 18	2,974 / 19
Overall R^2				0.074	0.113	0.093
Indiv./Country R^2				0.053/0.329	0.068/0.619	0.095/0.059
- 2 log likelihood				-16980.936	-13616.811	-13400.474

Standard errors in parentheses, *** p<0.001, ** p<0.01, * p<0.05, + p<0.1, see methods for variable definitions

For Review Only

Table A20. Robustness test with the value of Week variable adjusted for the country-level start of lockdown

	Models 1, 2 and 3			Model 1	Model 2	Model 3
	Adverse impact	Opportunity agility	Planning agility	Life satisfaction	Subjective vitality	Distress
Column	1	2	3	4	5	6
Severity of lockdown	0.162**	-0.076	0.087	0.113	0.128**	-0.071
	(0.062)	(0.138)	(0.058)	(0.134)	(0.048)	(0.051)
Adverse impact on business		-0.393***	0.211***	-0.253***	-0.085***	0.100***
		(0.041)	(0.018)	(0.036)	(0.015)	(0.013)
Opportunity agility				0.416***	0.237***	-0.120***
				(0.074)	(0.031)	(0.026)
Planning agility				-0.163***	-0.038*	0.071***
				(0.043)	(0.018)	(0.015)
Planning agility* Opp. agility				0.146*	0.069*	-0.038
				(0.070)	(0.030)	(0.025)
Controls						
GDP ppp pc 2019	-0.120	0.347+	0.086	0.057	-0.067	-0.033
	(0.089)	(0.200)	(0.084)	(0.195)	(0.071)	(0.076)
Government support	0.011	-0.160	-0.009	0.114	-0.051	0.040
	(0.080)	(0.180)	(0.076)	(0.175)	(0.060)	(0.064)
Firm age	0.039+	-0.163***	-0.024	-0.022	-0.029+	0.010
	(0.020)	(0.049)	(0.020)	(0.039)	(0.016)	(0.013)
Firm size (log employees)	-0.051**	0.315***	0.113***	0.120**	0.063***	-0.049***
	(0.020)	(0.047)	(0.020)	(0.039)	(0.016)	(0.014)
Industry: retail and gastronomy	0.119*	-0.391**	0.011	0.012	-0.006	0.083*
	(0.053)	(0.126)	(0.054)	(0.103)	(0.043)	(0.036)
Industry: manufacturing	0.005	-0.554***	0.046	0.167	0.065	0.047
	(0.054)	(0.126)	(0.054)	(0.104)	(0.043)	(0.036)
Industry: human oriented services	0.256***	-0.261+	0.016	0.128	-0.016	0.019
	(0.057)	(0.134)	(0.058)	(0.111)	(0.050)	(0.040)
Industry: other	0.015	-0.568**	-0.118	-0.187	0.006	0.150**
	(0.078)	(0.192)	(0.078)	(0.151)	(0.062)	(0.052)
Firm profit last year	-0.088*	-0.204*	-0.107**	0.187*	-0.011	-0.115***
	(0.040)	(0.093)	(0.040)	(0.078)	(0.033)	(0.028)
Entrepreneur gender (1=women)	0.044	0.134	0.001	-0.137+	-0.123***	0.129***
	(0.037)	(0.087)	(0.038)	(0.072)	(0.031)	(0.025)
Entrepreneur age	0.035+	-0.118*	-0.062**	0.094*	0.079***	-0.114***
	(0.021)	(0.049)	(0.021)	(0.041)	(0.017)	(0.014)
Secondary education	-0.070	-0.130	0.049	-0.106	-0.080	0.071
	(0.097)	(0.239)	(0.098)	(0.189)	(0.082)	(0.065)
University education	0.030	0.188+	0.062	0.175*	0.009	-0.015
	(0.042)	(0.102)	(0.042)	(0.081)	(0.035)	(0.028)
Week of data collection	-0.085*	0.093	-0.088*	0.137*	-0.006	0.003
	(0.035)	(0.080)	(0.035)	(0.069)	(0.028)	(0.025)
Constant	-0.028	-0.136	-0.055	6.377***	3.391***	2.728***
	(0.109)	(0.262)	(0.109)	(0.221)	(0.092)	(0.078)
N Individ./Countries	3,162 / 20	3,162 / 20	3,162 / 20	3,162 / 20	2,767 / 18	2,974 / 19
Overall R^2				0.072	0.110	0.093
Indiv./Country R^2				0.053/0.307	0.068/0.584	0.095/0.060
- 2 log likelihood				-16985.218	-13621.387	-13404.148

Standard errors in parentheses, *** p<0.001, ** p<0.01, * p<0.05, + p<0.1, see methods for variable definitions

For Review Only

Table A21. Longitudinal models

	Model 1 Opportunity agility	Model 2 Opportunity agility	Model 3 Planning agility	Model 4 Planning agility
Column	1	2	3	4
Adverse impact	-0.389*	-0.405*	0.446+	0.514*
	(0.181)	(0.189)	(0.252)	(0.251)
Pre-covid life satisfaction	0.263		-0.413	
	(0.283)		(0.370)	
Pre-covid vitality		0.688+		0.707
		(0.378)		(0.456)
Controls				
GDP ppp pc 2019	0.000	0.000	0.000+	0.000
	(0.000)	(0.000)	(0.000)	(0.000)
Firm age	-0.044	-0.029	-0.053	-0.056
	(0.030)	(0.029)	(0.040)	(0.040)
Firm size (log employees)	0.150	0.100	0.608*	0.574*
	(0.194)	(0.200)	(0.266)	(0.264)
Industry: retail and gastronomy	-1.096+	-1.301*	-0.689	-0.604
	(0.568)	(0.582)	(0.712)	(0.705)
Industry: manufacturing	0.217	0.300	0.995	0.839
	(0.559)	(0.562)	(0.796)	(0.776)
Industry: human oriented services	-0.046	-0.191	-0.129	-0.407
	(0.521)	(0.537)	(0.731)	(0.733)
Industry: other	0.208	-0.427	3.197+	3.175+
	(1.237)	(1.179)	(1.673)	(1.674)
Firm profit last year	0.215	0.312	0.116	0.151
	(0.398)	(0.410)	(0.543)	(0.540)
Entrepreneur gender (1=women)	-0.531	-0.572	0.277	0.199
	(0.385)	(0.391)	(0.535)	(0.527)
Entrepreneur age	-0.013	-0.019	0.001	-0.006
	(0.022)	(0.022)	(0.030)	(0.030)
Secondary education	0.740	0.666	1.227	1.272
	(0.899)	(0.907)	(1.192)	(1.167)
University education	-0.239	-0.300	-0.350	-0.382
	(0.510)	(0.514)	(0.678)	(0.655)
Week of data collection	-0.042	-0.031	-0.190	-0.178
	(0.066)	(0.067)	(0.094)	(0.093)
Covid life satisfaction	0.207		-0.002	
	(0.126)		(0.159)	
Covid vitality		0.588		-0.627
		(0.358)		(0.459)
Constant	-1.837	-2.452	2.451	2.299
	(2.848)	(2.915)	(3.972)	(3.972)
N	161	162	160	161
R^2			0.065	0.073
Snell R	0.172	0.214		

Standard errors in parentheses, *** p<0.001, ** p<0.01, * p<0.05, + p<0.1, see methods for variable definitions
The regressions for opportunity agility are binary logistic regressions

For Review Only

Table A22. Summary of indirect effects for robustness checks (H1, H4)

Robustness check	Indirect effect:	Life satisfaction	Subjective vitality	Distress
Stringency of lockdown replaced with the measure with the average from 7 months (January to July)	H1 multi-level indirect effect: Lockdown → Adverse impact → Wellbeing	B = -.043 $p < .05$	B = -.015 $p < .05$	B = .017 $p < .05$
	H4 multi-level indirect effect: Lockdown → Adverse impact → Agility → Wellbeing	B = -.027 $p < .05$	B = -.014 $p < .05$	B = .008 $p < .05$
	H4 lower-level relationships only: Adverse impact → Agility → Well-Being	B = -.198 $p < .05$	B = -.102 $p < .05$	B = .062 $p < .05$
Stringency of lockdown replaced with the measure with the average from 9 months (January to September)	H1 multi-level indirect effect: Lockdown → Adverse impact → Wellbeing	B = -.037 $p = .051$	B = -.013 $p = .053$	B = .015 $p = .050$
	H4 multi-level indirect effect: Lockdown → Adverse impact → Agility → Wellbeing	B = -.024 $p = .060$	B = -.012 $p = .057$	B = .007 $p = .062$
	H4 lower-level relationships only: Adverse impact → Agility → Well-Being	B = -.198 $p < .05$	B = -.101 $p < .05$	B = .062 $p < .05$
With only GDP as a control variable at the country level	H1 multi-level indirect effect: Lockdown → Adverse impact → Wellbeing	B = -.043 $p < .05$	B = -.015 $p < .05$	B = .017 $p < .05$
	H4 multi-level indirect effect: Lockdown → Adverse impact → Agility → Wellbeing	B = -.027 $p < .05$	B = -.014 $p < .05$	B = .008 $p < .05$
	H4 lower-level relationships only: Adverse impact → Agility → Well-Being	B = -.198 $p < .05$	B = -.102 $p < .05$	B = .062 $p < .05$
Number of Covid-19 cases per million people as a control variable	H1 multi-level indirect effect: Lockdown → Adverse impact → Wellbeing	B = -.049 $p < .05$	B = -.017 $p < .05$	B = .019 $p < .05$
	H4 multi-level indirect effect: Lockdown → Adverse impact → Agility → Wellbeing	B = -.031 $p < .05$	B = -.016 $p < .05$	B = .010 $p < .05$
	H4 lower-level relationships only: Adverse impact → Agility → Well-Being	B = -.199 $p < .05$	B = -.101 $p < .05$	B = .062 $p < .05$
Number of Covid-19 deaths per million people as a control variable	H1 multi-level indirect effect: Lockdown → Adverse impact → Wellbeing	B = -.053 $p < .05$	B = -.018 $p < .05$	B = .021 $p < .05$
	H4 multi-level indirect effect: Lockdown → Adverse impact → Agility → Wellbeing	B = -.034 $p < .05$	B = -.017 $p < .05$	B = .011 $p < .05$
	H4 lower-level relationships only: Adverse impact → Agility → Well-Being	B = -.198 $p < .05$	B = -.101 $p < .05$	B = .062 $p < .05$
Application for government support by the entrepreneur as a control variable	H1 multi-level indirect effect: Lockdown → Adverse impact → Wellbeing	B = -.040 $p < .05$	B = -.014 $p < .05$	B = .015 $p < .05$
	H4 multi-level indirect effect: Lockdown → Adverse impact → Agility → Wellbeing	B = -.026 $p < .05$	B = -.015 $p < .05$	B = .008 $p < .05$
	H4 lower-level relationships only: Adverse impact → Agility → Well-Being	B = -.194 $p < .05$	B = -.108 $p < .05$	B = .059 $p < .05$
Measure of adverse impact on business which combines the change in trading and threat to existence as the mean from z-standardized measures	H1 multi-level indirect effect: Lockdown → Adverse impact → Wellbeing	B = -.046 $p < .05$	B = -.015 $p < .05$	B = .018 $p < .05$
	H4 multi-level indirect effect: Lockdown → Adverse impact → Agility → Wellbeing	B = -.026 $p < .05$	B = -.013 $p < .05$	B = .008 $p < .05$
	H4 lower-level relationships only: Adverse impact → Agility → Well-Being	B = -.187 $p < .05$	B = -.096 $p < .05$	B = .059 $p < .05$
Measure of adverse impact on business which	H1 multi-level indirect effect: Lockdown → Adverse impact → Wellbeing	B = -.041 $p < .05$	B = -.014 $p < .05$	B = .016 $p < .05$

For Review Only

gives greater emphasis to the threat to the existence of the business	H4 multi-level indirect effect: Lockdown → Adverse impact → Agility → Wellbeing	B = -.023 p < .05	B = -.012 p < .05	B = .007 p < .05
	H4 lower-level relationships only: Adverse impact → Agility → Well-Being	B = -.177 p < .05	B = -.089 p < .05	B = .057 p < .05
Measure of adverse impact on business which gives the equal weight the change in trading and the threat to existence of the business	H1 multi-level indirect effect: Lockdown → Adverse impact → Wellbeing	B = -.044 p < .05	B = -.015 p < .05	B = .017 p < .05
	H4 multi-level indirect effect: Lockdown → Adverse impact → Agility → Wellbeing	B = -.026 p < .05	B = -.013 p < .05	B = .008 p < .05
	H4 lower-level relationships only: Adverse impact → Agility → Well-Being	B = -.193 p < .05	B = -.099 p < .05	B = .061 p < .05
Limited sample size to account for missing data for vitality and distress	H1 multi-level indirect effect: Lockdown → Adverse impact → Wellbeing		B = -.015 p < .05	B = .017 p < .05
	H4 multi-level indirect effect: Lockdown → Adverse impact → Agility → Wellbeing		B = -.014 p < .05	B = .009 p < .05
	H4 lower-level relationships only: Adverse impact → Agility → Well-Being		B = -.097 p < .05	B = .061 p < .05
Limited set of control variables	H1 multi-level indirect effect: Lockdown → Adverse impact → Wellbeing	B = -.043 p < .05	B = -.015 p < .05	B = .017 p < .05
	H4 multi-level indirect effect: Lockdown → Adverse impact → Agility → Wellbeing	B = -.027 p < .05	B = -.014 p < .05	B = .009 p < .05
	H4 lower-level relationships only: Adverse impact → Agility → Well-Being	B = -.201 p < .05	B = -.104 p < .05	B = .067 p < .05
Life satisfaction as an ordinal variable – logit regression	H1 multi-level indirect effect: Lockdown → Adverse impact → Wellbeing	B = -.044 p < .05		
	H4 multi-level indirect effect: Lockdown → Adverse impact → Agility → Wellbeing	B = -.027 p < .05		
	H4 lower-level relationships only: Adverse impact → Agility → Well-Being	B = -.198 p < .05		
Life satisfaction as an ordinal variable – probit regression	H1 multi-level indirect effect: Lockdown → Adverse impact → Wellbeing	B = -.025 p < .05		
	H4 multi-level indirect effect: Lockdown → Adverse impact → Agility → Wellbeing	B = -.014 p < .05		
	H4 lower-level relationships only: Adverse impact → Agility → Well-Being	B = -.106 p < .05		
Squared value of Week included	H1 multi-level indirect effect: Lockdown → Adverse impact → Wellbeing	B = -.044 p < .05	B = -.015 p < .05	B = .017 p < .05
	H4 multi-level indirect effect: Lockdown → Adverse impact → Agility → Wellbeing	B = -.027 p < .05	B = -.014 p < .05	B = .009 p < .05
	H4 lower-level relationships only: Adverse impact → Agility → Well-Being	B = -.198 p < .05	B = -.101 p < .05	B = .062 p < .05
The value of Week variable adjusted for the country-level start of lockdown	H1 multi-level indirect effect: Lockdown → Adverse impact → Wellbeing	B = -.051 p < .05	B = -.017 p < .05	B = .020 p < .05
	H4 multi-level indirect effect: Lockdown → Adverse impact → Agility → Wellbeing	B = -.032 p < .05	B = -.016 p < .05	B = .010 p < .05
	H4 lower-level relationships only: Adverse impact → Agility → Well-Being	B = -.198 p < .05	B = -.101 p < .05	B = .062 p < .05

For Review Only

Sample Comparisons & Representativeness against GEM entrepreneurs

As explained in the method section in the manuscript, to assess the representativeness of our sample we compared it to existing population-representative samples of individual entrepreneurs from the Global Entrepreneurship Monitor (GEM). To recap, our study focused on how the pandemic impacted entrepreneurs running operating businesses. To compare like-with-like, we limited the comparison sample of GEM entrepreneurs to new and established entrepreneurs who are running operating businesses (excluding nascent entrepreneurs who are the process of starting a business). Thus, we downloaded the GEM data files for the Adult Population Surveys to extract these samples. We did not use the GEM TEA rate. We pooled GEM data for 2011 to 2018 as participation of countries in GEM varies each year. Data for New Zealand was only available for 2005.

Tables A23 to 28 present country-by country comparisons of our sample and the GEM sample. For the overall sample comparisons across countries (row 'total' in Tables A23 to A28), we compute the mean of the country mean scores (thereby weighting each country equally). This accounts for the fact that the underlying country samples in GEM and in our study differ in sample size and thus avoids that the comparisons are biased by large country samples. For our sample this mean of country means corresponds to the means presented in Table 2 (whereas Table 1 provides means across individuals)[2].

Compared to GEM, entrepreneurs in our sample are slightly younger (43.51 years old as compared to 43.53 in GEM) and more often men (68.9% compared to 66.3% in GEM). Entrepreneurs in our sample are better educated (73.5% completed university education vs. 31.7% in GEM), and they lead larger (12.6 employees vs. 5.0 employees in GEM) and slightly younger (10.4 years vs. 11.1 years in GEM) businesses operating more frequently in service industries[3] (82.7% vs. 63.1% in GEM). Our survey targeted businesses and their owner-managers, whereas GEM obtains representative samples of individuals via households. The latter is more likely to identify self-employed which explains, for instance, the differences in business size between our and the GEM sample.

---

[2] The two values can diverge slightly, for example, the mean of the age of entrepreneurs across individuals in our sample is 43.48 (Table 1 in the manuscript), while the mean of country means is 43.51 (Table 2 in the manuscript and 'total' in Table A23). The mean age of the firm is 9.98 years across individual firms (Table 1 in the manuscript), while the mean of country means is 10.37 (Table 2 in the manuscript and 'total' in Table A24) etc.

[3] The industry categories used here in this comparison differ from the categories presented in the paper. This is due to the necessity to make industry categories comparable with the four categories used by GEM: extractive, transforming, business services and consumer-oriented services. Our categories include: manufacturing, retail and gastronomy, business services, human facing services, agriculture and extractive industries, and other. We recoded our category of retail and gastronomy into consumer-oriented services and agriculture and extractive industries into extractive industries. In the comparison of industry composition we do not take into consideration the entrepreneurs who indicated their industry sector as 'other' and did not provide any further details on the industry which would have enabled us to recode them into substantive categories.

For Review Only

Table A23. Age of the entrepreneur

	COVID-19				GEM			
	N	Mean	Std. Deviation	Std. Error Mean	N	Mean	Std. Deviation	Std. Error Mean
Australia	88	46.45	10.27	1.09	1381	47.78	13.71	0.37
Bangladesh	81	29.15***	7.65	0.850	364	36.70***	11.31	0.59
Bosnia-Herzegovina	89	45.28**	10.11	1.07	787	41.27**	11.47	0.41
Brazil	154	44.26***	10.54	0.849	11834	39.95***	11.76	0.11
Canada	276	48.31*	12.04	0.72	2063	46.42*	14.85	0.33
Chile	130	37.73***	7.95	0.70	8601	46.63***	14.34	0.15
China	469	33.38***	6.39	0.30	4809	40.54***	11.31	0.16
Colombia	84	39.82	10.59	1.15	3298	39.92	12.18	0.21
Denmark	134	53.60***	12.29	1.06	409	45.02***	11.12	0.55
France	111	50.71***	8.54	0.81	655	45.73***	12.05	0.47
Germany	211	46.62	9.75	0.67	2722	45.60	10.92	0.21
India	97	38.87	9.72	0.99	2174	37.54	11.34	0.24
Italy	135	41.02**	11.20	0.96	897	44.30**	10.57	0.35
Japan	71	46.21	14.85	1.76	907	48.63	10.64	0.35
New Zealand	118	49.81*	12.08	1.11	193	46.44*	9.52	0.69
Pakistan	93	36.04	8.99	0.93	258	35.50	12.24	0.76
Poland	85	40.93	9.57	1.04	3269	42.53	10.48	0.18
Spain	215	46.40	11.20	0.76	18788	45.63	10.30	0.08
UK	374	48.47	10.82	0.56	4768	47.14	12.89	0.19
USA	147	47.06	11.94	0.99	3637	47.75	14.06	0.23
**Total[1]**	**3162**	**43.51****	**12.09**	**0.22**	**72733**	**43.53****	**12.55**	**0.05**

* $p \leq .05$, ** $p \leq .01$, *** $p \leq .001$. [1]This is the mean of country means corresponding to Table 2 (but not Table 1) in the manuscript, please see detailed explanation on page 28 (second paragraph) of this online supplement.

For Review Only

Table A24. Age of the firm

	COVID-19				GEM			
	N	Mean	Std. Deviation	Std. Error Mean	N	Mean	Std. Deviation	Std. Error Mean
Australia	88	5.98***	4.25	0.45	1326	11.83***	13.59	0.37
Bangladesh	81	5.43**	7.94	0.88	364	7.97**	8.13	0.43
Bosnia-Herzegovina	89	13.64***	10.95	1.16	765	8.48***	8.40	0.30
Brazil	154	11.08***	12.29	0.99	11637	7.73***	8.71	0.08
Canada	276	12.68	12.65	0.76	1681	13.00	14.32	0.35
Chile	130	3.38***	7.68	0.67	8054	8.84***	12.32	0.14
China	469	5.79	4.50	0.21	4447	5.45	5.78	0.09
Colombia	84	7.07	7.21	0.79	3058	6.13	8.08	0.15
Denmark	134	11.66	9.29	0.80	386	10.37	11.08	0.56
France	111	17.88**	19.84	1.88	616	11.38**	11.89	0.48
Germany	211	12.25	7.84	0.54	2652	12.94	14.96	0.29
India	97	6.45***	9.46	0.96	2031	14.72***	32.28	0.72
Italy	135	6.73***	9.99	0.86	724	16.64***	14.64	0.54
Japan	71	28.39**	23.43	2.78	827	20.07**	22.11	0.77
New Zealand	118	11.41*	12.30	1.13	192	8.40*	10.64	0.77
Pakistan	93	8.40	9.22	0.96	208	7.75	7.79	0.54
Poland	85	9.67*	7.87	0.85	3063	11.89*	10.68	0.19
Spain	215	12.23*	11.86	0.81	17135	14.07*	14.05	0.11
UK	374	11.60	10.04	0.52	4276	11.83	14.65	0.22
USA	147	5.69***	3.51	0.29	3429	14.64***	17.08	0.29
**Total[1]**	**3162**	**10.37*****	**12.33**	**0.22**	**67350**	**11.13*****	**14.70**	**0.06**

* $p \leq .05$, ** $p \leq .01$, *** $p \leq .001$. [1]This is the mean of country means corresponding to Table 2 (but not Table 1) in the manuscript, please see detailed explanation on page 28 (second paragraph) of this online supplement.

Table A25. Size of the business

	COVID-19				GEM			
	N	Mean	Std. Deviation	Std. Error Mean	N	Mean	Std. Deviation	Std. Error Mean
Australia	88	9.32	18.15	1.93	1402	6.48	19.51	0.52
Bangladesh	81	24.59**	58.13	6.46	364	2.29**	6.88	0.36
Bosnia-Herzegovina	89	23.78***	47.32	5.02	623	5.18***	9.36	0.37
Brazil	154	6.05***	14.65	1.18	11696	0.94***	3.93	0.04
Canada	276	9.28	18.59	1.12	1879	8.58	25.21	0.58
Chile	130	7.51	15.59	1.37	8366	4.89	13.50	0.15
China	469	30.65***	46.82	2.16	4446	6.99***	16.34	0.25
Colombia	84	10.27*	21.13	2.31	3158	4.15*	9.40	0.17
Denmark	134	6.37	17.77	1.53	401	5.22	19.06	0.95
France	111	6.76	16.78	1.59	641	4.53	15.11	0.60
Germany	211	4.79	19.55	1.35	2669	6.71	17.65	0.34
India	97	21.36***	29.77	3.02	1882	2.55***	3.73	0.09
Italy	135	7.37	20.39	1.75	857	6.08	17.12	0.58
Japan	71	19.56	71.89	8.53	846	8.77	22.31	0.77
New Zealand	118	10.17**	14.14	1.30	189	5.14**	12.68	0.92
Pakistan	93	22.68***	46.42	4.81	239	3.67***	6.65	0.43
Poland	85	9.08*	20.24	2.20	3224	4.21*	11.80	0.21
Spain	215	2.58	5.86	0.40	18249	2.91	9.92	0.07
UK	374	10.60***	21.71	1.12	4776	5.40***	18.02	0.26
USA	147	9.61	27.66	2.28	3633	7.00	20.89	0.35
**Total[1]**	**3162**	**12.62*** **	**33.08**	**0.59**	**69522**	**5.07*** **	**15.33**	**0.06**

* $p \leq .05$, ** $p \leq .01$, *** $p \leq .001$. [1] This is the mean of country means please see detailed explanation on page 28 (second paragraph) of this online supplement. This mean does not correspond to Table 2 in the paper, as Table 2 reports the logged value.

For Review Only

Table A26. Gender

		COVID-19			GEM		
		Count	%	Standardized Residual	Count	%	Standardized Residual
Australia Chi-square = 2.64, df = 1, p = .104	Male	63	71.6%	1.0	913	63.0%	-0.2
	Female	25	28.4%	-1.3	536	37.0%	0.3
	Total	88	100.0%		1449	100.0%	
Bangladesh Chi-square = 1.01, df = 1, p = .315	Male	74	91.4%	0.3	318	87.4%	-0.1
	Female	7	8.6%	-0.9	46	12.6%	0.4
	Total	81	100.0%		364	100.0%	
Bosnia-Herzegovina Chi-square = 12.80, df = 1, p < .001	Male	74*	83.1%	2.0	506	64.2%	-0.7
	Female	15**	16.9%	-2.8	282	35.8%	0.9
	Total	89	100.0%		788	100.0%	
Brazil Chi-square = 13.09, df = 1, p < .001	Male	58*	37.7%	-2.5	6205	52.3%	0.3
	Female	96**	62.3%	2.6	5655	47.7%	-0.3
	Total	154	100.0%		11860	100.0%	
Canada Chi-square = 0.35, df = 1, p = .554	Male	169	61.2%	0.4	1248	59.4%	-0.1
	Female	107	38.8%	-0.4	854	40.6%	0.2
	Total	276	100.0%		2102	100.0%	
Chile Chi-square = 8.83, df = 1, p = .003	Male	95	73.1%	1.9	5250	60.2%	-0.2
	Female	35*	26.9%	-2.3	3465	39.8%	0.3
	Total	130	100.0%		8715	100.0%	
China Chi-square = 7.40, df = 1, p = .007	Male	299	63.8%	1.7	2773	57.3%	-0.5
	Female	170*	36.2%	-2.0	2070	42.7%	0.6
	Total	469	100.0%		4843	100.0%	
Colombia Chi-square = 4.46, df = 1, p = .035	Male	60	71.4%	1.3	1954	60.0%	-0.2
	Female	24	28.6%	-1.6	1302	40.0%	0.3
	Total	84	100.0%		3256	100.0%	
Denmark Chi-square = 0.490, df = 1, p = .484	Male	100	74.6%	0.3	286	71.5%	-0.2
	Female	34	25.4%	-0.5	114	28.5%	0.3
	Total	134	100.0%		400	100.0%	
France Chi-square = 3.73, df = 1, p = .053	Male	64	57.7%	-1.0	434	67.1%	0.4
	Female	47	42.3%	1.4	213	32.9%	-0.6
	Total	111	100.0%		647	100.0%	
Germany Chi-square = 12.01, df = 1, p = .001	Male	113*	53.6%	-2.0	2292	65.3%	0.5
	Female	98**	46.4%	2.7	1218	34.7%	-0.7
	Total	211	100.0%		3510	100.0%	
India Chi-square = 16.34, df = 1, p < .001	Male	84*	86.6%	2.2	1458	67.0%	-0.5
	Female	13***	13.4%	-3.3	718	33.0%	0.7
	Total	97	100.0%		2176	100.0%	
Italy Chi-square = 0.28, df = 1, p = .597	Male	99	73.3%	0.3	648	71.1%	-0.1
	Female	36	26.7%	-0.4	263	28.9%	0.2
	Total	135	100.0%		911	100.0%	
Japan Chi-square = 0.71, df = 1, p = .401	Male	48	67.6%	-0.4	659	72.3%	0.1
	Female	23	32.4%	0.7	253	27.7%	-0.2
	Total	71	100.0%		912	100.0%	
New Zealand Chi-square = 0.70, df = 1, p = .402	Male	81	68.6%	0.4	106	63.9%	-0.3
	Female	37	31.4%	-0.5	60	36.1%	0.4
	Total	118	100.0%		166	100.0%	
Pakistan Chi-square = 2.36,	Male	80	86.0%	-0.4	229	91.6%	0.3
	Female	13	14.0%	1.2	21	8.4%	-0.8

For Review Only

df = 1, p = .124	Total	93	100.0%		250	100.0%	
Poland<br>Chi-square = 2.60,<br>df = 1, p = .107	Male	47	55.3%	-1.0	2204	63.8%	0.2
	Female	38	44.7%	1.3	1250	36.2%	-0.2
	Total	85	100.0%		3454	100.0%	
Spain<br>Chi-square = 1.65,<br>df = 1, p = .199	Male	120	55.8%	-0.8	11597	60.1%	0.1
	Female	95	44.2%	1.0	7690	39.9%	-0.1
	Total	215	100.0%		19287	100.0%	
UK<br>Chi-square = 5.79,<br>df = 1, p = .016	Male	275	73.5%	1.3	3014	67.5%	-0.4
	Female	99	26.5%	-1.9	1452	32.5%	0.6
	Total	374	100.0%		4466	100.0%	
USA<br>Chi-square = 7.74,<br>df = 1, p = .005	Male	106	72.1%	1.7	2273	60.7%	-0.3
	Female	41*	27.9%	-2.1	1471	39.3%	0.4
	Total	147	100.0%		3744	100.0%	
**Total[1]**<br>**Chi-square = 9.38,**<br>**df = 1, p = .002**	**Male**	**2179**	**68.9%**	**1.7**	**48587**	**66.3%**	**-0.4**
	**Female**	**983***	**31.1%**	**-2.4**	**24713**	**33.7%**	**0.5**
	**Total**	**3162**	**100.0%**		**76462**	**100.0%**	

* *p* ≤ .05, standardized residual ≥ 2.0; ** *p* ≤ .01, standardized residual ≥ 2.6; *** *p* ≤ .001, standardized residual ≥ 3.3.
[1] This is the mean of country means corresponding to Table 2 (but not Table 1) in the manuscript, please see detailed explanation on page 28 (second paragraph) of this online supplement.

For Review Only

Table A27. Education

		COVID-19			GEM		
		Count	%	Standardized Residual	Count	%	Standardized Residual
Australia<br>Chi-square = 35.37<br>df = 3, p < 0.001	Primary, lower secondary	1***	1.1%	-3.3	225	15.6%	0.8
	Secondary	12	13.6%	-0.3	215	14.9%	0.1
	Post secondary	13*	14.8%	-2.4	416	28.9%	0.6
	Tertiary	62***	70.5%	4.1	583	40.5%	-1.0
	Total	88	100.0%		1439	100.0%	
Bangladesh<br>Chi-square = 124.40<br>df = 3, p < 0.001	Primary, lower secondary	4***	4.9%	-5.1	120**	55.8%	3.2
	Secondary	14	17.3%	-1.7	66	30.7%	1.0
	Post secondary	12***	14.8%	4.8	0**	0.0%	-3.0
	Tertiary	51***	63.0%	6.2	29***	13.5%	-3.8
	Total	81	100.0%		215	100.0%	
Bosnia-Herzegovina<br>Chi-square = 163.53<br>df = 3, p < 0.001	Primary, lower secondary	0**	0.0%	-3.1	93	12.0%	1.0
	Secondary	20***	22.5%	-4.9	536	68.9%	1.7
	Post secondary	10	11.2%	1.7	48	6.2%	-0.6
	Tertiary	59***	66.3%	10.5	101***	13.0%	-3.6
	Total	89	100.0%		778	100.0%	
Brazil<br>Chi-square = 977.73<br>df = 2, p < 0.001	Primary, lower secondary	3***	1.9%	-8.2	5154	47.6%	1.0
	Secondary	14***	9.1%	-6.3	4606	42.6%	0.8
	Tertiary	137***	89.0%	29.3	1063***	9.8%	-3.5
	Total	154	100.0%		10823	100.0%	
Canada<br>Chi-square = 48.22<br>df = 3, p < 0.001	Primary, lower secondary	2**	0.7%	-2.9	100	4.9%	1.1
	Secondary	21**	7.6%	-3.0	311	15.3%	1.1
	Post secondary	36**	13.0%	-3.0	459	22.5%	1.1
	Tertiary	217***	78.6%	4.0	1168	57.3%	-1.5
	Total	276	100.0%		2038	100.0%	
Chile<br>Chi-square = 172.28<br>df = 3, p < 0.001	Primary, lower secondary	3***	2.3%	-3.4	1118	13.4%	0.4
	Secondary	0***	0.0%	-6.3	2554	30.7%	0.8
	Post secondary	6***	4.6%	-3.7	1579	19.0%	0.5
	Tertiary	121***	93.1%	10.2	3081	37.0%	-1.3
	Total	130	100.0%		8332	100.0%	
China<br>Chi-square = 1082.99<br>df = 3, p < 0.001	Primary, lower secondary	6***	1.3%	-11.4	1586***	33.1%	3.6
	Secondary	30**	6.4%	-9.9	1679**	35.0%	3.1
	Post secondary	112	23.9%	1.6	972	20.3%	-0.5
	Tertiary	321***	68.4%	27.5	555***	11.6%	-8.6
	Total	469	100.0%		4792	100.0%	
Colombia<br>Chi-square = 165.02<br>df = 3, p < 0.001	Primary, lower secondary	1***	1.2%	-4.0	686	21.8%	0.7
	Secondary	1***	1.2%	-4.8	950	30.2%	0.8
	Post secondary	4**	4.8%	-3.1	630	20.0%	0.5
	Tertiary	78***	92.9%	10.6	883	28.0%	-1.7
	Total	84	100.0%		3149	100.0%	
Denmark<br>Chi-square = 106.65<br>df = 3, p < 0.001	Primary, lower secondary	1	0.7%	1.4	0	0.0%	-0.9
	Secondary	5*	3.7%	-2.5	50	13.7%	1.5
	Post secondary	19***	14.2%	-5.3	204**	55.7%	3.2
	Tertiary	109***	81.3%	6.5	112***	30.6%	-3.9
	Total	134	100.0%		366	100.0%	
France<br>Chi-square = 16.74<br>df = 3, p = 0.001	Primary, lower secondary	4**	3.6%	-2.8	96	15.0%	1.2
	Secondary	27*	24.3%	2.5	86	13.5%	-1.0
	Post secondary	26	23.4%	-0.2	156	24.5%	0.1
	Tertiary	54	48.6%	0.2	300	47.0%	-0.1
	Total	111	100.0%		638	100.0%	

For Review Only

For Review Only

Germany	Primary, lower secondary	17***	8.1%	-3.9	681	20.9%	1.0
Chi-square = 136.98	Secondary	23***	10.9%	-5.5	1102	33.8%	1.4
df = 3, p < 0.001	Post secondary	40	19.0%	0.0	622	19.1%	0.0
	Tertiary	131***	62.1%	9.1	860*	26.3%	-2.3
	Total	211	100.0%		3265	100.0%	
India	Primary, lower secondary	2***	2.1%	-5.5	738	36.5%	1.2
Chi-square = 321.83	Secondary	2***	2.1%	-5.4	721	35.7%	1.2
df = 3, p < 0.001	Post secondary	2*	2.1%	-2.4	204	10.1%	0.5
	Tertiary	91***	93.8%	15.6	357***	17.7%	-3.4
	Total	97	100.0%		2020	100.0%	
Italy	Primary, lower secondary	1***	0.7%	-6.2	312*	34.4%	2.4
Chi-square = 236.37	Secondary	18***	13.3%	-4.9	400	44.2%	1.9
df = 3, p < 0.001	Post secondary	6**	4.4%	2.7	10	1.1%	-1.1
	Tertiary	110***	81.5%	11.6	184***	20.3%	-4.5
	Total	135	100.0%		906	100.0%	
Japan	Primary, lower secondary	6	8.5%	1.2	45	5.0%	-0.3
Chi-square = 9.40	Secondary	12*	16.9%	-2.3	303	33.9%	0.7
df = 3, p = 0.024	Post secondary	16	22.5%	0.4	179	20.0%	-0.1
	Tertiary	37	52.1%	1.3	367	41.1%	-0.4
	Total	71	100.0%		894	100.0%	
New Zealand	Primary, lower secondary	9***	7.6%	-3.3	52**	32.3%	2.8
Chi-square = 51.53	Secondary	8	6.8%	-0.6	15	9.3%	0.5
df = 3, p < 0.001	Post secondary	23***	19.5%	4.3	0***	0.0%	-3.6
	Tertiary	78	66.1%	0.6	94	58.4%	-0.5
	Total	118	100.0%		161	100.0%	
Pakistan	Primary, lower secondary	5***	5.4%	-5.1	94***	57.0%	3.9
Chi-square = 147.38	Secondary	9**	9.7%	-2.9	54*	32.7%	2.2
df = 3, p < 0.001	Post secondary	25***	26.9%	3.3	11*	6.7%	-2.5
	Tertiary	54***	58.1%	7.0	6***	3.6%	-5.2
	Total	93	100.0%		165	100.0%	
Poland	Primary, lower secondary	1*	1.2%	-2.3	269	8.6%	0.4
Chi-square = 106.18	Secondary	9***	10.6%	-5.2	1606	51.4%	0.9
df = 3, p < 0.001	Post secondary	5	5.9%	-1.2	310	9.9%	0.2
	Tertiary	70***	82.4%	8.4	939	30.1%	-1.4
	Total	85	100.0%		3124	100.0%	
Spain	Primary, lower secondary	23***	10.7%	-6.3	7014	36.9%	0.7
Chi-square = 120.05	Secondary	41	19.1%	0.4	3410	18.0%	0.0
df = 3, p < 0.001	Post secondary	21*	9.8%	-2.4	3124	16.5%	0.3
	Tertiary	130***	60.5%	8.6	5441	28.7%	-0.9
	Total	215	100.0%		18989	100.0%	
UK	Primary, lower secondary	22***	5.9%	-6.7	1034*	23.8%	2.0
Chi-square = 115.23	Secondary	40***	10.7%	-3.7	861	19.8%	1.1
df = 3, p < 0.001	Post secondary	40	10.7%	0.2	450	10.4%	-0.1
	Tertiary	272***	72.7%	6.8	2002*	46.1%	-2.0
	Total	374	100.0%		4347	100.0%	
USA	Primary, lower secondary	0**	0.0%	-2.6	178	4.9%	0.5
Chi-square = 66.43	Secondary	13*	8.8%	-2.5	642	17.8%	0.5
df = 3, p < 0.001	Post secondary	4***	2.7%	-4.9	796	22.1%	1.0
	Tertiary	130***	88.4%	5.2	1985	55.1%	-1.0
	Total	147	100.0%		3601	100.0%	
**Total[1]**	**Primary, lower secondary**	**107*****	**3.4%**	**-22.3**	**15521*****	**22.8%**	**4.8**
**Chi-square =**	**Secondary**	**339*****	**10.7%**	**-18.8**	**20044*****	**29.5%**	**4.1**
**2492.79**	**Post secondary**	**392*****	**12.4%**	**-4.9**	**10925**	**16.1%**	**1.1**
**df = 3, p < 0.001**	**Tertiary**	**2323*****	**73.5%**	**38.8**	**21534*****	**31.7%**	**-8.4**
	**Total**	**3162**	**100.0%**		**68024**	**100.0%**	

* $p \leq .05$, standardized residual ≥ 2.0; ** $p \leq .01$, standardized residual ≥ 2.6; *** $p \leq .001$, standardized residual ≥ 3.3.

[1] This is the mean of country means corresponding to Table 2 (but not Table 1) in the manuscript, please see detailed explanation on page 28 (second paragraph) of this online supplement.

Table A28. Industry

		COVID-19			GEM		
		Count	%	Standardized Residual	Count	%	Standardized Residual
Australia<br>Chi-square = 50.49<br>df = 3, p < 0.001	Extractive	0**	0.0%	-2.8	145	10.1%	0.7
	Transforming	5***	6.0%	-3.7	405	28.1%	0.9
	Business Services	56***	67.5%	5.1	469	32.6%	-1.2
	Consumer Oriented	22	26.5%	-0.4	420	29.2%	0.1
	Total	83	100.0%		1439	100.0%	
Bangladesh<br>Chi-square = 16.37<br>df = 3, p = 0.001	Extractive	16	24.2%	1.1	63	17.3%	-0.5
	Transforming	19*	28.8%	2.1	57	15.7%	-0.9
	Business Services	5	7.6%	1.8	10	2.7%	-0.8
	Consumer Oriented	26*	39.4%	-2.2	234	64.3%	0.9
	Total	66	100.0%		364	100.0%	
Bosnia-Herzegovina<br>Chi-square = 103.32<br>df = 3, p < 0.001	Extractive	1***	1.3%	-4.2	209	26.9%	1.3
	Transforming	23	28.8%	-0.1	229	29.5%	0.0
	Business Services	35***	43.8%	8.6	62**	8.0%	-2.8
	Consumer Oriented	21	26.3%	-1.3	277	35.6%	0.4
	Total	80	100.0%		777	100.0%	
Brazil<br>Chi-square = 167.46<br>df = 3, p < 0.001	Extractive	0	0.0%	-1.7	243	2.1%	0.2
	Transforming	16***	10.7%	-4.9	4033	34.3%	0.5
	Business Services	56***	37.6%	11.8	1000	8.5%	-1.3
	Consumer Oriented	77	51.7%	-0.6	6466	55.1%	0.1
	Total	149	100.0%		11742	100.0%	
Canada<br>Chi-square = 106.74<br>df = 3, p < 0.001	Extractive	10	3.8%	-1.8	127	7.1%	0.7
	Transforming	20***	7.5%	-4.1	356	19.9%	1.6
	Business Services	194***	72.9%	7.2	702**	39.3%	-2.8
	Consumer Oriented	42***	15.8%	-4.5	600	33.6%	1.7
	Total	266	100.0%		1785	100.0%	
Chile<br>Chi-square = 147.51<br>df = 3, p < 0.001	Extractive	7	5.4%	0.8	343	4.0%	-0.1
	Transforming	19**	14.6%	-3.0	2516	29.1%	0.4
	Business Services	78***	60.0%	10.7	1598	18.5%	-1.3
	Consumer Oriented	26***	20.0%	-4.6	4202	48.5%	0.6
	Total	130	100.0%		8659	100.0%	
China<br>Chi-square = 25.07<br>df = 3, p < 0.001	Extractive	11	2.6%	-1.2	181	3.8%	0.3
	Transforming	86	20.2%	-1.3	1132	23.5%	0.4
	Business Services	60***	14.1%	4.4	359	7.5%	-1.3
	Consumer Oriented	269	63.1%	-0.5	3138	65.2%	0.1
	Total	426	100.0%		4810	100.0%	
Colombia<br>Chi-square = 39.58<br>df = 3, p < 0.001	Extractive	4	4.8%	0.6	111	3.5%	-0.1
	Transforming	16*	19.3%	-2.3	1103	34.4%	0.4
	Business Services	31***	37.3%	5.6	437	13.6%	-0.9
	Consumer Oriented	32	38.6%	-1.3	1558	48.6%	0.2
	Total	83	100.0%		3209	100.0%	
Denmark<br>Chi-square = 46.15<br>df = 3, p < 0.001	Extractive	3**	2.4%	-2.6	48	12.1%	1.5
	Transforming	17	13.6%	-1.5	86	21.6%	0.9
	Business Services	84***	67.2%	4.4	134*	33.7%	-2.5
	Consumer Oriented	21*	16.8%	-2.5	130	32.7%	1.4
	Total	125	100.0%		398	100.0%	
France<br>Chi-square = 52.95	Extractive	4*	3.6%	-2.5	83	13.1%	1.0
	Transforming	14*	12.6%	-2.2	156	24.5%	0.9

For Review Only

df = 3, p < 0.001	Business Services	21*	18.9%	-2.3	209	32.9%	0.9
	Consumer Oriented	72***	64.9%	5.4	188*	29.6%	-2.2
	Total	111	100.0%		636	100.0%	
Germany	Extractive	0**	0.0%	-2.8	133	4.0%	0.7
Chi-square = 52.77	Transforming	11***	5.5%	-5.0	766	23.2%	1.2
df = 3, p < 0.001	Business Services	97***	48.3%	4.0	1035	31.3%	-1.0
	Consumer Oriented	93	46.3%	1.0	1370	41.5%	-0.2
	Total	201	100.0%		3304	100.0%	
India	Extractive	4	4.2%	-2.0	181	11.4%	0.5
Chi-square = 505.74	Transforming	14	14.7%	-0.7	287	18.0%	0.2
df = 3, p < 0.001	Business Services	66***	69.5%	20.8	73***	4.6%	-5.1
	Consumer Oriented	11***	11.6%	-6.3	1053	66.1%	1.5
	Total	95	100.0%		1594	100.0%	
Italy	Extractive	5*	4.1%	-2.1	94	10.8%	0.8
Chi-square = 75.19	Transforming	13**	10.6%	-3.2	234	26.9%	1.2
df = 3, p < 0.001	Business Services	84***	68.3%	6.6	251***	28.9%	-2.5
	Consumer Oriented	21**	17.1%	-2.8	290	33.4%	1.1
	Total	123	100.0%		869	100.0%	
Japan	Extractive	3	6.1%	0.5	41	4.6%	-0.1
Chi-square = 4.94	Transforming	8	16.3%	-1.7	263	29.8%	0.4
df = 3, p = 0.176	Business Services	19	38.8%	1.3	249	28.2%	-0.3
	Consumer Oriented	19	38.8%	0.1	331	37.4%	0.0
	Total	49	100.0%		884	100.0%	
New Zealand	Extractive	4**	3.4%	-3.1	35**	22.3%	2.7
Chi-square = 43.82	Transforming	14*	11.9%	-2.2	44	28.0%	1.9
df = 3, p < 0.001	Business Services	70**	59.3%	3.2	41**	26.1%	-2.8
	Consumer Oriented	30	25.4%	0.2	37	23.6%	-0.2
	Total	118	100.0%		157	100.0%	
Pakistan	Extractive	7*	9.5%	-2.4	63	26.7%	1.3
Chi-square = 45.30	Transforming	17	23.0%	-1.2	79	33.5%	0.7
df = 3, p < 0.001	Business Services	23***	31.1%	5.2	11**	4.7%	-2.9
	Consumer Oriented	27	36.5%	0.1	83	35.2%	-0.1
	Total	74	100.0%		236	100.0%	
Poland	Extractive	0**	0.0%	-2.6	291	8.5%	0.4
Chi-square = 58.86	Transforming	10**	12.0%	-3.2	1106	32.4%	0.5
df = 3, p < 0.001	Business Services	46***	55.4%	6.3	742	21.7%	-1.0
	Consumer Oriented	27	32.5%	-0.7	1273	37.3%	0.1
	Total	83	100.0%		3412	100.0%	
Spain	Extractive	8*	3.9%	-2.5	1744	9.1%	0.3
Chi-square = 43.66	Transforming	21***	10.2%	-4.0	4566	23.9%	0.4
df = 3, p < 0.001	Business Services	82***	39.8%	4.5	4595	24.1%	-0.5
	Consumer Oriented	95	46.1%	0.7	8187	42.9%	-0.1
	Total	206	100.0%		19092	100.0%	
UK	Extractive	6*	1.7%	-2.2	180	4.1%	0.6
Chi-square = 287.96	Transforming	29***	8.1%	-6.6	1181	27.2%	1.9
df = 3, p < 0.001	Business Services	294***	82.1%	12.6	1584***	36.5%	-3.6
	Consumer Oriented	29***	8.1%	-7.6	1400*	32.2%	2.2
	Total	358	100.0%		4345	100.0%	
USA	Extractive	0**	0.0%	-3.0	225	6.4%	0.6
Chi-square = 94.85	Transforming	4***	2.7%	-5.0	812	23.0%	1.0
df = 3, p < 0.001	Business Services	114***	77.6%	7.1	1371	38.8%	-1.5
	Consumer Oriented	29*	19.7%	-2.5	1130	31.9%	0.5
	Total	147	100.0%		3538	100.0%	
**Total**	**Extractive**	**112*** **	**3.8%**	**-10.8**	**7177***	**10.4%**	**2.2**
**Chi-square = 95.86**	**Transforming**	**399*** **	**13.5%**	**-13.2**	**18572** **	**26.5%**	**2.7**
**df = 3, p < 0.001**	**Business Services**	**1490*** **	**50.6%**	**30.7**	**15538*** **	**22.1%**	**-6.3**

For Review Only

	**Consumer Oriented**	**945*** **	**32.1%**	**-7.3**	**28803**	**41.0%**	**1.5**
	**Total**	**2946**	**100.0%**		**70190**	**100.0%**	

* $p \leq .05$, standardized residual ≥ 2.0; ** $p \leq .01$, standardized residual ≥ 2.6; *** $p \leq .001$, standardized residual ≥ 3.3

For Review Only

Convergent and Discriminant Validity of Agility Measures

Table A29. Convergent and discriminant validity among independent and dependent variables based on (Fornell & Larcker, 1981)

	F1	F2	F3	F4	F5
**F1: Life Satisfaction**	**1.000**				
**F2: Adverse Impact**	0.072	**0.505**			
**F3: Opportunity Agility**	0.020	0.060	**1.000**		
**F4: Planning Agility**	0.006	0.078	0.039	**1.000**	
**F5: Stringency**	0.000	0.000	0.007	0.015	**1.000**
	**F1**	**F2**	**F3**	**F4**	**F5**
**F1: Vitality**	**0.584**				
**F2: Adverse Impact**	0.038	**0.501**			
**F3: Opportunity Agility**	0.024	0.047	**1.000**		
**F4: Planning Agility**	0.000	0.080	0.042	**1.000**	
**F5: Stringency**	0.017	0.004	0.000	0.013	**1.000**
	**F1**	**F2**	**F3**	**F4**	**F5**
**F1: Distress**	**0.417**				
**F2: Adverse Impact**	0.106	**0.507**			
**F3: Opportunity Agility**	0.008	0.040	**1.000**		
**F4: Planning Agility**	0.024	0.083	0.042	**1.000**	
**F5: Stringency**	0.000	0.001	0.004	0.014	**1.000**

Note: AVE (average variance extracted) is shown in the bold diagonal of the matrix. The rest of the numbers show the SC (squared correlations) between factors. When AVE values are >= SC, as in all cases in our results, there is no problem with discriminant validity.

For Review Only

Table A30. Convergent and discriminant correlations of agility with creativity, effectuation and causation

	**Convergent correlations**			**Discriminant correlation**
	**Creativity**	**Effectuation – flexibility**	**Effectuation - experimentation**	**Causation**
Example item	*"Did you create new ideas for difficult issues?"*	*"I was flexible and took advantage of opportunities as they arose"*	*"I tried a number of different approaches until I found a business model that worked."*	*"I designed and planned business strategies."*
**Opportunity agility**	**.323*****	**.209***	.035	.109n.s.
**Planning agility**	**.214*****	.135	**.262****	.135n.s.
**N**	2754	98	98	97
**Sample**	18 countries (excluding Germany and USA)	India	India	India
**Source of creativity and effectuation causation scales**	Janssen (2000), and Weinberger et al. (2018), JBV)	Effectuation-causation scale: Laskovaia et al., (2017, 2019), which is an abbreviated version of the effectuation scale developed by Chandler et al., (2011) JBV		
